# Initial impressions of compatibility and mate value predict later dating and romantic interest

**DOI:** 10.1073/pnas.2206925119

**Published:** 2022-11-02

**Authors:** Alexander Baxter, Jessica A. Maxwell, Karen L. Bales, Eli J. Finkel, Emily A. Impett, Paul W. Eastwick

**Affiliations:** ^a^California National Primate Research Center, Neuroscience and Behavior Division, Davis, CA 95616;; ^b^Department of Psychology, University of California, Davis, CA 95616;; ^c^School of Psychology, University of Auckland, Auckland 1010, New Zealand;; ^d^Department of Neurobiology, Physiology, and Behavior, University of California, Davis, CA 95616;; ^e^Department of Psychology, Northwestern University, Evanston, IL 60208;; ^f^Kellogg School of Management, Northwestern University, Evanston, IL 60208;; ^g^Department of Psychology, University of Toronto Mississauga, Mississauga, ON M5S 1A4, Canada

**Keywords:** first impressions, initial attraction, social relations model, compatibility, pair bonding

## Abstract

Romantic first impressions seem to linger, but why? Few studies have investigated how romantic desire during initial interactions predicts later relational outcomes (e.g., later romantic interest, contact attempts) using a design that can tease apart different possible mechanisms (e.g., mate value, selectivity, compatibility). Across three speed-dating studies (*n* = 559) with longitudinal follow-ups (including college and community samples, and a sample of men who date men), we investigated whether different components of initial romantic impressions predicted later romantic outcomes and relationship initiation. Using the social relations model, we partitioned initial desire at speed dating (determined from 6,600+ total dates) into partner effects (a date’s consensual desirability, e.g., mate value), actor effects (a participant’s general desirousness, e.g., selectivity), and relationship effects (a participant’s unique liking for a date over and beyond partner and actor effects, e.g., compatibility) to predict later evaluations (romantic interest, physical attraction, and desire to know better) and behaviors (direct messaging and going on dates). Meta-analyses across the three studies showed that, across 6,100+ follow-up reports, partner and relationship effects were especially strong predictors of relationship initiation variables. Consistent with evolutionary models of human pair bonding, these findings suggest that both consensually desirable traits and unique impressions of compatibility have lingering effects on relationship development, even from the moment that two potential partners meet.

## Significance

Romantic first impressions seem to linger, but why? Thus far, few studies have distinguished between lingering impressions based on a potential partner’s consensually attractive qualities (popularity) vs. a person’s general desirousness (choosiness) vs. a person uniquely liking a potential partner over and beyond the partner’s popularity and the person’s choosiness (compatibility). In this study, we meta-analyzed three speed-dating studies with longitudinal follow-ups to investigate how these three factors contribute to the romantic initiation process. We found that relationship effects and partner effects were the strongest predictors of later romantic desire and dating. These findings suggest that both compatibility and mate value shape human mating decisions, even from a first impression.

“You don’t get a second chance at making a good first impression.” This oft-repeated statement implies that perceivers’ first impressions endure: Other people’s perceptions of us predict their subsequent thoughts, feelings, and behaviors toward us. Evidence for this idea pervades the person-perception literature: People exhibit consensus about other people’s desirable attributes (e.g., physical attractiveness) after very brief “thin slice” exposures, and these initial judgments predict distal outcomes like popularity and occupational success (1, 2). In the relationship initiation domain, potential suitors message attractive online daters (3), presumably anticipating that their initial positive online impression will subsequently translate to a positive offline impression. First impressions have particularly high stakes in romantic contexts; after all, many relationships begin in “open-field” settings (e.g., at a bar or on a dating app) where people can disengage from future interactions with a potential partner if first impressions go awry (4, 5). People want to convey positive first impressions—seemingly, for good reason.

## Using the Social Relations Model to Explain How First Impressions Endure

Why exactly do first impressions endure? The thin slices and attractiveness examples are premised on one mechanism: that perceivers can detect the target’s true qualities (e.g., competence, attractiveness), and these qualities remain impactful at later time points. For example, a potential partner’s natural charisma on a first date might make a good first impression (6), and this same charisma will likely persist and elicit further favorable evaluations as the relationship continues. Such partner effects are surely important factors in shaping romantic judgments and decisions over time, but they are not the only route by which romantic first impressions might linger.

According to the social relations model (SRM) (7–9), three routes could produce lingering first impressions—not just partner effects, but also actor and relationship effects. Actor effects reflect general and stable tendencies for a perceiver to give low vs. high ratings. For example, one person might think that everyone in their dating pool is unappealing, on average [e.g., those who perceive themselves as highly desirable (10)], whereas another might feel they have an appealing dating pool, on average [e.g., those who strongly crave or are “desperate” to be in a relationship (11)]. These perceivers’ first impressions would also linger, but it would be because they impose their own dispositional evaluative tendencies on potential partners.

First impressions might also endure through relationship effects, which reflect unique liking (i.e., above and beyond actor and partner effects). For example, imagine a woman, “Sally,” who generally finds her dating pool unappealing (an actor effect), and imagine a man, “Joe,” who is not consensually desirable (a partner effect). Sally could still be attracted to Joe, despite her own selective disposition and his lack of popularity (a relationship effect), perhaps due to a sparkling initial conversation or learning that they share a niche interest (e.g., a love of Akira Kurosawa films). In this case, first impressions could linger because she is especially compatible with him, and whatever dyadic factors inspire this sense of initial compatibility might operate similarly as a romantic relationship later develops.

## Theoretical Rationales Underlying the Predictive Power of Partner, Actor, and Relationship Effects

Romantic first impressions contain all three sources of variance in SRM designs [i.e., studies in which each participant rates each potential romantic partner, as in speed dating (9)]; initial attraction reliably features partner, actor, and relationship effects. But it remains unclear exactly what these three components separately predict during the relationship initiation process, and there are distinct rationales underlying the potential predictive power of each component. For example, partner effects are central to the concept of mate value (12). If a potential mate has stable traits that affect their romantic desirability, then perceivers should exhibit consensus about those traits, and the same potential mates who are initially desirable should also be desirable at subsequent time points. Actor effects reflect schemas and expectations that affect how perceivers generally view potential partners: For example, popular people might learn that they can afford to be selective in their romantic overtures (13), and they might apply their selectivity to initial and later impressions alike.

Unlike actor and partner effects, relationship effects are not tied to the classic trait-based conceptualization of mate value but rather to chemistry and compatibility (14, 15). A core theme in relationship science is that compatibility is essential—that most relationships succeed or fail because two people fit well or poorly together (16, 17). Compatibility is also important in an evolutionary context, and likely had adaptive value (18). Unlike most primates, human mating evolved in the context of monogamous pair-bonded relationships (19–21), as investment from both parents was likely needed for highly dependent offspring to survive (22, 23). Hence, in choosing a mate, early humans not only had to consider whether a potential mate had attractive attributes (e.g., traits that indicated fertility and good genes) but also whether that potential mate would make a suitable partner—someone they could form an enduring partnership with for long enough to ensure their offspring’s (as well as their own) survival. According to this perspective, as long as a potential mate was attractive enough to be a viable reproductive partner (i.e., healthy, fertile, and unrelated), dyadic compatibility was the primary force driving human pair bonding and attachment (24).

Just how much do romantic first impressions linger because of a potential partner’s consensually desirable traits (partner effects) vs. the perceiver’s dispositions (actor effects) vs. compatibility (relationship effects)? A longitudinal study design is needed to answer these questions, one in which perceivers rate targets at an initial time point and later report on romantic relationship development. Although relatively few such studies exist, there is some evidence that initial measures (i.e., assessed before or shortly after two potential partners meet) of perceived physical attractiveness (25), perceived similarity (26, 27), and self-reported long-term and short-term relationship goals (28) predict subsequent attempts at future interaction and/or romantic interest. However, none of these studies adequately differentiated partner, actor, and relationship effects, and so the mechanisms by which initial romantic impressions might linger remain empirically indistinguishable.

## The Current Research

**Fig. 1.**
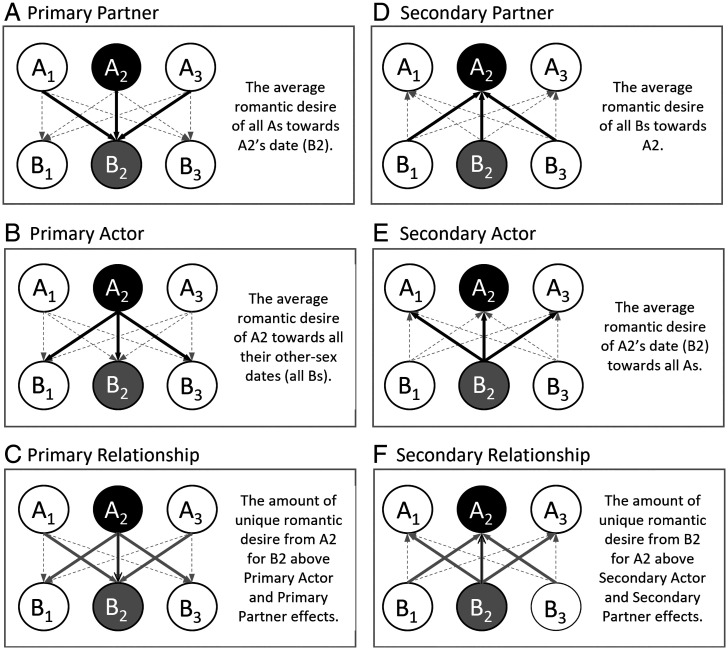
Schematic showing the SRM effects for initial desire. The figure demonstrates how the three primary SRM effects (*A–C*; i.e., the SRM calculations based on participants’ desire for their dates) and three secondary SRM effects (*D*–*F*; i.e., the SRM calculations based on participants’ dates’ desire for the participants) examined in this study were calculated for a hypothetical participant in the mixed-gender speed-dating events (A2, represented by the black circle) and one of their other-sex dates from the speed-dating event (B2, represented by the gray circle). A1 and A3 represent other participants of the same gender as A2, and B1 and B3 represent participants of the same gender as B2 (i.e., other-gender dates). In this example, each A went on a speed date with each B, but As and Bs did not go on speed dates with each other (as in the mixed-gender speed-dating events). Solid black arrows represent responses that are included in the SRM calculation for variance in A2’s romantic desire toward B2, and dashed gray arrows represent responses that are not involved in the SRM calculations. In the case of primary and secondary relationship effects, the solid gray arrows represent responses that are involved in partitioning variance (i.e., the respective partner and actor effects) but are not indicative of the overall meaning of the relationship effect (i.e., relationship effects reflect unique liking over and beyond partner and actor effects). The primary and secondary SRM effects were calculated similarly for the all-men speed-dating event, except with a slight correction for the round-robin design (see ref. 44).

To address this gap, we examined whether partner, actor, and relationship effects in romantic first impressions of potential partners at a speed-dating event predicted later evaluations and attempts to initiate a relationship. Speed-dating studies are well suited to the SRM method because asymmetric-block and round-robin rating designs allow researchers to simultaneously quantify partner, actor, and relationship effects (Fig. 1). Across three datasets spanning college and community samples and diverse sexual orientations (*SI Appendix*, Table S1), we investigated whether these three components of initial desire (measured immediately after each speed date) predicted future relationship initiation behaviors (initiating contact, interacting, and meeting up) and romantic evaluations (romantic desire, physical attraction, and desire to know a partner better), measured on various dichotomous and continuous scales (see *Materials and Methods*). Although two prior articles have reported SRM analyses using the data from studies 1 to 3 (29–31), no previous investigations from any of the present studies have used SRM components to predict later outcomes determined from the follow-up surveys (see *Materials and Methods*).

In addition to these “primary” research questions, we also explored whether participants’ later romantic interests and relationship initiation attempts were predicted by their potential romantic partner’s reports of initial desire, which we label “secondary” partner, actor, and relationship effects (Fig. 1). In other words, while the primary research questions pertained to whether a person’s later romantic interest was predicted by the SRM components of their own initial desire for their date, the secondary research questions pertained to whether a person’s later romantic interest was predicted by the SRM components of their date’s initial desire for them (hence, the secondary SRM components may be considered measures of “reflective initial desire”). For example, people may be especially motivated to pursue a romantic relationship with a potential partner that they believe uniquely likes them (i.e., a secondary relationship effect), and they may lose interest if they think that they left a positive first impression because of a secondary actor effect (e.g., “they only liked me because they like everyone”) or a secondary partner effect (e.g., “they only liked me because I am popular”). Indeed, being uniquely liked seems more likely to inspire positive reciprocity than unselective forms of liking (29).

Finally, we also explored whether perceived attraction from speed-dating matches (after the event) was associated with initial desire (and its underlying primary and secondary SRM components). This variable (i.e., perceived desire) contributes to relationship initiation through many pathways (32, 33) (for more information on the rationale for these analyses, see *SI Appendix*, Note 1). However, it is conceptually distinct from the other romantic outcome variables because it is based on interpreting another person’s behaviors and feelings, rather than reporting on one’s own behaviors and feelings. In these analyses, we tested 1) whether people projected their own initial desire onto perceptions of their match’s desire for them (i.e., the primary SRM variables predicting later perceived attraction from the match) and 2) whether people could accurately detect a match’s lingering attraction for them (i.e., the secondary SRM variables predicting later perceived attraction from the match).

## Results

To address these research questions, we performed exploratory multilevel regressions in one dataset (study 1), and then we preregistered our analysis plans for the subsequent two datasets (studies 2 and 3; https://osf.io/3zyn6/?view_only=6fddb1a6f1e94abf9a04fe68c99c1218). We focus our interpretation on effect sizes and 95% CIs after meta-analyzing across the three studies.

### Meta-analyses of Primary Partner, Actor, and Relationship Effects on Romantic Outcomes.

**Fig. 2.**
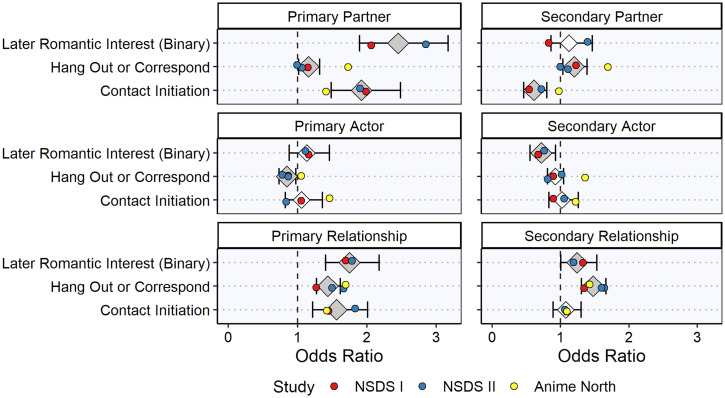
Meta-analysis of primary and secondary initial desire variables associated with dichotomous outcome variables. The figure shows the average effect sizes (diamonds) and 95% CIs (horizontal error bars) for the meta-analyzed associations between each primary and secondary component of initial desire (the predictor in each analysis) and each dichotomous outcome assessed. Individual effect sizes for each study are also shown (circles). The dark gray diamonds indicate meta-analyzed odds ratios that are significantly different from one (*P* < 0.05), and the light gray diamonds indicate meta-analyzed odds ratios that are not significantly different from one (*P* ≥ 0.05). For a summary of each analysis and the meta-analyzed effect sizes, see Table 1 (for more detailed information, see *SI Appendix*, Table S2).

**Fig. 3.**
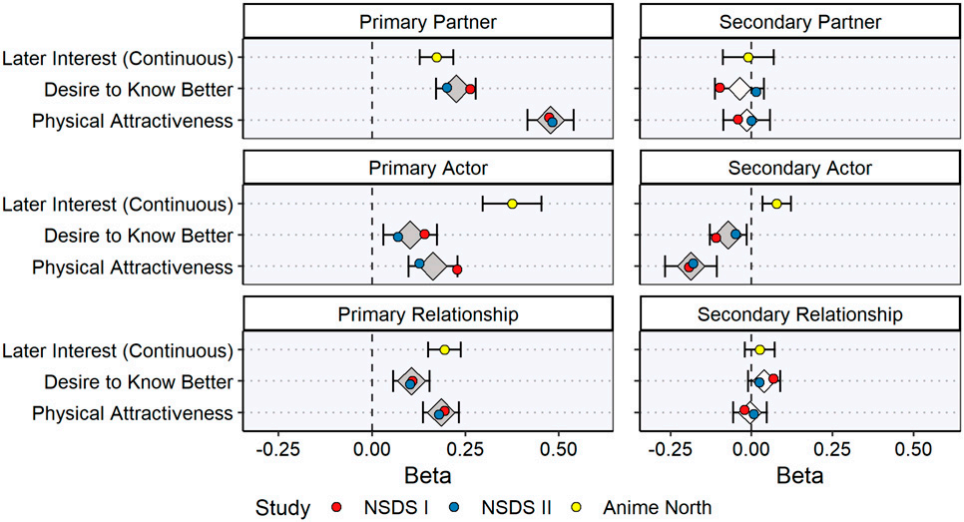
Meta-analysis of primary and secondary initial desire variables associated with continuous outcome variables. The figure shows the average effect sizes (diamonds) and 95% CIs (horizontal error bars) for the meta-analyzed associations between each primary and secondary component of initial desire (the predictor in each analysis) and each continuous outcome assessed. Individual effect sizes for each study are also shown (circles). The dark gray diamonds indicate meta-analyzed beta coefficients that are significantly different from zero (*P* < 0.05), and the light gray diamonds indicate meta-analyzed beta coefficients that are not significantly different from zero (*P* ≥ 0.05). For a summary of each analysis and the meta-analyzed effect sizes, see Table 2 (for more detailed information, see *SI Appendix*, Table S3).

A summary of the meta-analyzed results for the primary SRM components of initial desire is given in Tables 1 and 2 and Figs. 2 and 3 (see also *SI Appendix*, Tables S2 and S3). The primary partner effects (i.e., B’s average desirability across all As) robustly predicted both the dichotomous outcomes (i.e., initiating contact, hanging out/corresponding, and yes/no reports of romantic desire; meta-analytic odds ratios [ORs] ranged from 1.15 to 2.45) and the continuous outcomes (i.e., wanting to get to know the match better and ratings of the match’s physical attractiveness; meta-analytic βs ranged from 0.22 to 0.48). The primary actor effects (i.e., A’s average desire toward all Bs; Fig. 1) tended not to predict the dichotomous outcomes (meta-analytic ORs 0.84 to 1.13), but they did predict the continuous outcomes (meta-analytic βs 0.10 to 0.16). Finally, the primary relationship effects (i.e., A’s unique desire for B) also robustly predicted both the dichotomous (meta-analytic ORs 1.43 to 1.75) and continuous (meta-analytic βs 0.11 to 0.18) outcomes. Generally speaking, the partner effects were somewhat stronger predictors than the relationship effects, which were, in turn, stronger than the actor effects. In other words, over the days and weeks following the speed-dating events, participants initiated contact and experienced romantic desire to the extent that 1) their matches were consensually desirable (primary partner effects), 2) they uniquely desired the matches (primary relationship effects), and 3) they themselves were generally desirous people (primary actor effects).

**Table 1.** Results for **initial desire predicting dichotomous outcomes across the meta-analyses and individual studies**

IV and DV	Meta-analysis				Study 1	Study 2*	Study 3
	OR	95% CI	*z*	*p*	OR	OR	OR
Primary partner							
Contact initiation	1.92	[1.48, 2.48]	4.97	< 0.001	1.98	1.90	1.41
Hang out or correspond	1.15	[1.01, 1.32]	2.14	0.032	1.15	0.99, 1.06	1.73
Later romantic interest (binary)	2.45	[1.89, 3.17]	6.79	< 0.001	2.06	2.85	—
Primary actor							
Contact initiation	1.05	[0.82, 1.36]	0.41	0.683	1.05	0.83	1.46
Hang out or correspond	0.84	[0.73, 0.97]	2.35	0.019	0.86	0.86, 0.78	1.05
Later romantic interest (binary)	1.13	[0.88, 1.46]	0.94	0.347	1.16	1.11	—
Primary relationship							
Contact initiation	1.56	[1.21, 2.01]	3.47	0.001	1.44	1.83	1.42
Hang out or correspond	1.43	[1.27, 1.62]	5.91	<0.001	1.27	1.66, 1.49	1.69
Later romantic interest (binary)	1.75	[1.40, 2.17]	4.98	<0.001	1.69	1.78	—
Secondary partner							
Contact initiation	0.61	[0.46, 0.80]	3.56	<0.001	0.54	0.72	0.97
Hang out or correspond	1.20	[1.03, 1.39]	2.37	0.018	1.22	0.99, 1.11	1.69
Later romantic interest (binary)	1.12	[0.86, 1.46]	0.85	0.398	0.83	1.39	—
Secondary actor							
Contact initiation	1.02	[0.83, 1.26]	0.19	0.849	0.89	1.05	1.22
Hang out or correspond	0.92	[0.81, 1.05]	1.26	0.208	0.89	1.01, 0.81	1.35
Later romantic interest (binary)	0.72	[0.55, 0.93]	2.55	0.011	0.67	0.76	—
Secondary relationship							
Contact initiation	1.08	[0.89, 1.30]	0.76	0.448	1.08	1.06	1.09
Hang out or correspond	1.47	[1.31, 1.66]	6.31	<0.001	1.34	1.60, 1.64	1.42
Later romantic interest (binary)	1.24	[1.00, 1.53]	1.99	0.046	1.33	1.19	—

The table displays the results of the meta-analyses of the romantic dichotomous outcome variables for each IV (the main stub column entries) and each outcome DV, as well as a summary of the analyses across each individual study (for a detailed report of these analyses, see *SI Appendix*, Table S2). All numbers displayed in the table are rounded to two decimal places, except for the *p* values, which are rounded to three decimal places. Dashes (—) indicate no data.

^*^In study 2, individual estimates were obtained for hanging out (the first value, before the comma) and corresponding (the second value, after the comma).

**Table 2.** Results for **initial desire predicting continuous outcomes across the meta-analyses and individual studies**

IV and DV	Meta-analysis				Study 1	Study 2	Study 3
	Beta	95% CI	*z*	*p*	Beta	Beta	Beta
Primary partner							
Later interest (continuous)	—	—	—	—	—	—	0.17
Know better	0.22	[0.17, 0.28]	8.30	<0.001	0.26	0.20	—
Physical attractiveness	0.48	[0.42, 0.54]	15.23	<0.001	0.47	0.48	—
Primary actor							
Later interest (continuous)	—	—	—	—	—	—	0.37
Know better	0.10	[0.03, 0.17]	2.78	0.005	0.14	0.07	—
Physical attractiveness	0.16	[0.10, 0.23]	4.86	<0.001	0.23	0.12	—
Primary relationship							
Later interest (continuous)	—	—	—	—	—	—	0.19
Know better	0.11	[0.06, 0.15]	4.28	<0.001	0.11	0.10	—
Physical attractiveness	0.18	[0.14, 0.23]	7.59	<0.001	0.19	0.18	—
Secondary partner							
Later interest (continuous)	—	—	—	—	—	—	−0.01
Know better	−0.04	[−0.11, 0.04]	0.95	0.340	−0.10	0.01	—
Physical attractiveness	−0.01	[−0.09, 0.06]	0.40	0.691	−0.04	0.00	—
Secondary actor							
Later interest (continuous)	—	—	—	—	—	—	0.08
Know better	−0.07	[−0.13, −0.01]	2.45	0.014	−0.11	−0.05	—
Physical attractiveness	−0.19	[−0.27, −0.11]	4.58	<0.001	−0.19	−0.18	—
Secondary relationship							
Later interest (continuous)	—	—	—	—	—	—	0.03
Know better	0.04	[−0.01, 0.09]	1.54	0.124	0.07	0.02	—
Physical attractiveness	−0.01	[−0.06, 0.05]	0.21	0.838	−0.02	0.01	—

The table displays the results of the meta-analyses of the continuous romantic outcome variables for each IV (the main stub column entries) and each outcome DV, as well as a summary of the analyses across each individual study (for a detailed report of these analyses, see *SI Appendix*, Table S3). All numbers displayed in the table are rounded to two decimal places, except for the *p* values, which are rounded to three decimal places. Dashes (—) indicate no data.

### Meta-analyses of Secondary Partner, Actor, and Relationship Effects on Romantic Outcomes.

A summary of the meta-analyzed results for the secondary SRM components of initial desire is given in Tables 1 and 2 and Figs. 2 and 3 (see also *SI Appendix*, Tables S2 and S3). The secondary partner effects (i.e., A’s average desirability across all Bs) showed variable associations with the dichotomous outcomes (meta-analytic ORs 0.61 to 1.20), and they did not predict the continuous outcomes strongly (meta-analytic βs ranged from −0.04 to 0.01). The secondary actor effects (i.e., B’s average desire toward all As) tended not to predict the dichotomous outcomes (meta-analytic ORs ranged from 0.72 to 1.02), but they did (negatively) predict the continuous outcomes (meta-analytic βs ranged from −0.07 to −0.19). Finally, the secondary relationship effects (i.e., B’s unique desire for A) generally predicted the dichotomous outcomes (meta-analytic ORs 1.08 to 1.47) but did not predict the continuous outcomes (meta-analytic βs ranged from −0.01 to 0.04). The secondary effects tended to be weaker than the primary effects, but there was some evidence that participants 1) initiated contact with matches who uniquely desired them at the speed-dating event (secondary relationship effects) and 2) evaluated desirous people less positively (secondary actor effects). Participants’ own level of consensual desirability (secondary partner effects, i.e., the participant’s “popularity”) did not exhibit any consistent association with initiating contact or experiencing romantic desire.

### Meta-analyses of Perceived Attraction from Match.

**Fig. 4.**
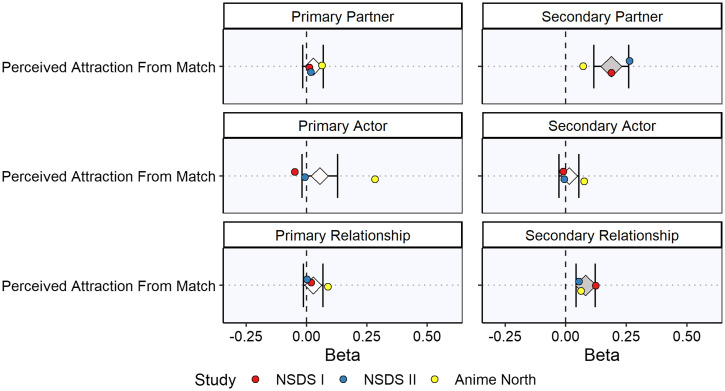
Meta-analysis of primary and secondary initial desire variables associated with perceived attraction from match. The figure shows the average effect sizes (diamonds) and 95% CIs (horizontal error bars) for the meta-analyzed associations between each primary and secondary component of initial desire (the predictor in each analysis) and each continuous outcome assessed. Individual effect sizes for each study are also shown (circles). The dark gray diamonds indicate that meta-analyzed beta coefficients that are significantly different from zero (*P* < 0.05), and the light gray diamonds indicate meta-analyzed beta coefficients that are not significantly different from zero (*P* ≥ 0.05). For a summary of each analysis and the meta-analyzed effect sizes, see Table 3 (for more detailed information, see *SI Appendix*, Table S4).

A summary of these meta-analyzed results is given in Table 3 and Fig. 4 (see also *SI Appendix*, Tables S4). The best predictors of perceived attraction from the match were the secondary partner effects (i.e., A’s average desirability across all Bs; meta-analytic β = 0.19, *P* < 0.001) and the secondary relationship effects (i.e., B’s unique desire for A; meta-analytic β = 0.08, *P* < 0.001). In other words, over the days and weeks following the speed-dating events, participants perceived that their matches had greater interest in them to the extent that 1) they themselves were a consensually desirable person at speed dating (secondary partner effects) and 2) their matches uniquely desired them at speed dating (secondary relationship effects). These results suggest that people 1) have some sense of their own desirability to others and 2) can accurately detect when specific people desire them. However, the effect sizes were moderate, and there were no associations with the primary SRM variables (Table 3). In other words, there was no evidence that participants projected their own lingering desire for a match onto the desire they perceived from that match. See *SI Appendix*, Note 2 for further commentary on these results.

**Table 3.** Results for initial desire predicting perceived attraction from match in the meta-analyses and individual studies

	Perceived attraction from match						
IV and DV	Meta-analysis				Study 1	Study 2	Study 3
	Beta	95% CI	*z*	*p*	Beta	Beta	Beta
Primary partner	0.03	[−0.02, 0.07]	1.25	0.210	0.01	0.02	0.06
Primary actor	0.05	[−0.02, 0.13]	1.45	0.147	−0.05	−0.01	0.28
Primary relationship	0.03	[−0.01, 0.07]	1.34	0.181	0.02	0.00	0.09
Secondary partner	0.19	[0.12, 0.26]	5.10	<0.001	0.19	0.26	0.07
Secondary actor	0.01	[−0.03, 0.05]	0.66	0.511	−0.01	−0.01	0.08
Secondary relationship	0.08	[0.04, 0.12]	4.04	<0.001	0.12	0.06	0.06

The table displays the results of the meta-analyses of the continuous romantic outcome variables for each IV (the stub column entries) and each outcome DV, as well as a summary of the analyses across each individual study (for a detailed report of these analyses, see *SI Appendix*, Table S4). All numbers displayed in the table are rounded to two decimal places, except for the *p* values, which are rounded to three decimal places.

### Meta-analyses of Gender Differences.

Sex and gender differences feature prominently in some evolutionary models of human mating (34), and so we preregistered that we would test for moderation by gender for each analysis. A summary of these analyses is given in *SI Appendix*, Tables S5 and S6. Only four out of 36 meta-analytic interactions between an SRM independent variable (IV) and gender were significant. Further, we note that, when we probed these interactions, there were no consistent patterns of gender differences for any particular SRM variable or outcome variable. For further commentary on these analyses, see *SI Appendix*, Note 3.

### Sensitivity Analyses.

We repeated each analysis to assess whether the original results would change if we 1) included random slopes for each SRM predictor (within participant ID and partner ID) in each model; 2) included all six of the original primary and secondary SRM variables as simultaneous predictors of each outcome in a series of maximal models to account for reciprocal processes influencing initial desire (i.e., associations between the three primary SRM components and the three secondary SRM components of initial desire; *SI Appendix*, Table S8); or 3) calculated the SRM effects separately for initial romantic liking and initial sexual desire, rather than using the average level of “initial desire” as in the original analyses (we also considered later romantic and sexual desire separately for the follow-up outcome variables, when possible). The results of the original analyses were largely unchanged in the random slopes models (*SI Appendix*, Fig. S2 and Tables S9 and S10) or the maximal models (*SI Appendix*, Fig. S3 and Tables S11 and S12 *A–D*). When we considered initial romantic liking and initial sexual desire separately, the effect sizes for the SRM components of initial sexual desire were slightly larger than those for initial romantic liking (*SI Appendix*, Fig. S4 and Tables S13 and S14); however, the overall pattern of results for each variable was still similar to the pattern of the original analyses. For further commentary on the results of these sensitivity analyses and their consistency with the original analyses, see *SI Appendix*, Note 4.

## Discussion

**Fig. 5.**
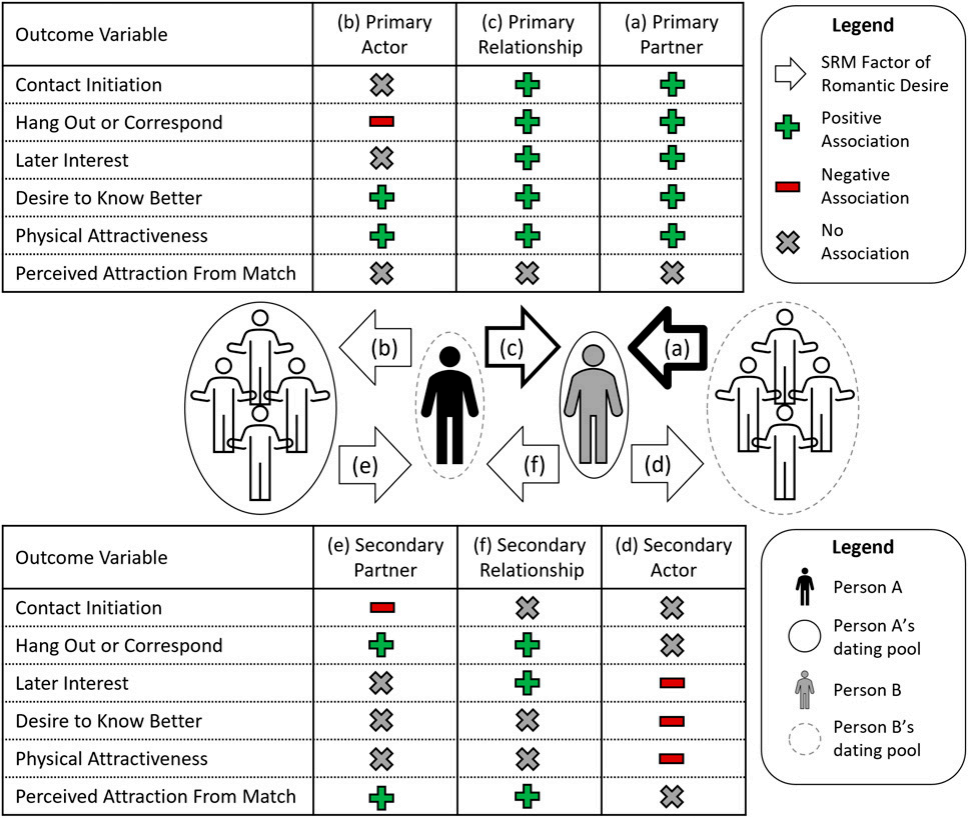
Graphic summary of study results. The figure shows a visual summary of the study results. The two tables depict the various meta-analyses that were conducted. Each row represents one cluster of variables that were meta-analyzed, and each column represents the IV used in the individual studies to predict the outcome. Each cell in the table represents the general result of one meta-analysis. Green plus signs indicate a significant positive association between the variable indicated in the column and the outcome indicated in the row; red minus signs indicate a significant negative association, and gray X symbols represent no significant association. The various human figures represent either a focal participant (“Person A”), a potential partner (“Person B”), or all the other people in Person A’s or Person B’s dating pool (see the figure legend). Note that, in this figure, we depicted Person A’s and Person B’s dating pools as mutually exclusive (as would be the case for a completely heterosexual man and woman), but, in some cases, Person A’s and Person B’s dating pools might overlap (for example, in the case of two homosexual, bisexual, or pansexual people). The arrows represent the six SRM components of initial desire (primary and secondary partner, actor, and relationship effects—the variables used as predictors of later romantic interest and relationship initiation behavior). For example, for component a—primary partner effects—the arrow represents the degree of consensual romantic desire that Person B receives from all people in their dating pool (including Person A and others) (for more explanation, see Fig. 1). The arrows with thicker lines (components a and c) represent the relatively stronger effect sizes found for these predictors (Tables 1 and 2).

In a large, combined dataset with over 550 participants and 6,600+ total speed dates, we investigated whether lingering first impressions (and the SRM components that underlie them) predicted later romantic judgments and relationship initiation attempts. We found that primary partner effects (average effect size *r* = 0.22 across *SI Appendix*, Tables S2*A* and S3*A*) and primary relationship effects (average effect size *r* = 0.15 across *SI Appendix*, Tables S2*C* and S3*C*; see *SI Appendix*, Note 5 for a description of these calculations) were the best predictors (Fig. 5). In other words, Joe would be most likely to pursue a relationship with Sally if 1) Sally was consensually desirable (primary partner effect) and 2) Joe uniquely desired Sally (primary relationship effect). These findings are consistent with existing perspectives that prioritize the evolutionary relevance of both partner and relationship effects (35). Because certain traits signal mate value (e.g., attractiveness, fertility, resource potential; see ref. 19), they can be quickly and consensually assessed in a first encounter and inspire a lingering first impression (36). However, compatibility also matters for human pair bonding (24), as early hominids needed enduring partnerships to successfully rear offspring (19). Our findings add to burgeoning research showing that both consensual and relational factors affect how humans evaluate and behave toward others (14, 37), even from the moment that two potential partners meet.

Beyond the strong influences of consensus and compatibility, primary actor effects and secondary relationship effects were more modest but generally positive predictors of later romantic outcomes, and secondary actor effects were generally negative predictors (Fig. 5). In other words, Joe would be somewhat likely to pursue a relationship with Sally and evaluate it positively if 1) Joe was a generally desirous person (primary actor effects), 2) Sally uniquely desired Joe (secondary relationship effects), and 3) Sally was not a generally desirous person (secondary actor effects). In the context of our findings concerning perceived desire (*SI Appendix*, Note 2), these results affirm that people notice and appreciate unique liking and partner selectivity (29, 38), even from the start of a potential relationship.

Our findings are robust across three independent studies, including a community-based sample (study 3) and one that included an all-men speed-dating event (study 2). Another strength is that our analyses were preregistered in studies 2 and 3. Further, our work employs the SRM to prospectively study how different facets of very first romantic impressions lead people to pursue (or not to pursue) relationships (based on behavioral measures of relationship initiation), and our findings add to substantial literature showing that even brief impressions (i.e., formed after 3- to 4-min speed dates) can be impressive predictors of later outcomes (see ref. 1). However, an important caveat is that we did not assess how impressions (and their underlying SRM factors) changed after speed dating: We presume that a positive first impression lingers, motivating desire to meet again and inspiring positive evaluations at subsequent meetings. Although this premise is empirically supported (e.g., ref. 39), first impressions are also bound to change, as reflected by the overall small-to-moderate effect sizes in the current study. For example, research suggests that relationship effects wax and partner effects wane as people get to know each other (14, 40). Future studies should incorporate how impressions change during relationship initiation. Another important question that remains is how first impressions drive relationship initiation when potential partners already have a platonic relationship. The effects of age, culture, and other diverse aspects of identity also merit further investigation. subsample of (presumably) gay or bisexual men

In sum, we found that impressions based on a potential mate’s consensual desirability and on unique compatibility were the strongest predictors of romantic outcomes. We also found contributions of people’s general desirousness and reciprocated liking. Taken together, these studies demonstrate that romantic first impressions—especially the popularity and compatibility components of those impressions—are an important factor in the early stages of relationship development.

## Materials and Methods

All procedures were reviewed and approved by the institutional review boards at Northwestern University (studies 1 and 2) and the University of Toronto (study 3). In each study, all participants gave informed consent in line with the requirements of ethical approval.

### Participants and Procedures.

For detailed information about the number of participants in each study and other demographic information, see *SI Appendix*, Table S1.

#### Studies 1 and 2: NSDS I and NSDS II.

Student’s attending Northwestern University participated in one of two experimental speed-dating studies: the Northwestern Speed-Dating Study I (NSDS I) [(47); see Finkel et al. (41)] or the NSDS II [(47); see Tidwell et al. (42)]. In study 1, all speed-dating events were mixed gender. In study 2, eight of the speed-dating events were mixed gender, and one of the events was same gender (only men). At each mixed-gender speed-dating event, participants went on 4-min speed dates with each member of the other sex; in the men-only event, 12 participants went on 4-min round-robin speed dates with each of the 11 other men. After each of their 9 to 13 speed dates, participants completed a brief survey assessing initial desire for each date. Within 24 h, participants indicated on a website which of their dates they wished to interact with further. Participants that “matched” (i.e., mutual “yeses”) could use an online messaging system to contact each other. Following the speed-dating event, participants that matched were sent follow-up surveys (study 1: 10 times over 1 mo; study 2: 12 times over 4 mo; *SI Appendix*, Table S1 and Fig. S1 *A* and *B*) and reported on their relationship with each match. Participants were compensated for attending the speed-dating event and for completing follow-up surveys (study 1: between $5 and $45; study 2: between $10 and $56; *SI Appendix*, Table S1). Although these datasets have been in previous publications (e.g., refs. 25, 29, 30, 40, 43), no prior articles used initial desire (measured at speed dating) to prospectively predict relationship initiation (measured during the follow-up period), as was done in the current study.

#### Study 3: Anime North.

People attending the Toronto 2015 Anime North comic book convention who had signed up to go to a mixed-gender speed-dating event were invited to participate in a research study (details were similar to ref. 11). Procedures were largely similar to studies 1 and 2, except for the following differences: Study 3 participants went on 3-min speed dates, and the brief postdate survey contained both the initial measure of romantic desire and the “yes/no” item to determine matching. Shortly after the event, participants that matched were given each other’s personal contact information, and they completed 12 weekly follow-up surveys (*SI Appendix*, Fig. S1*C*) and were compensated according to the number of surveys completed (between $5 and $25; *SI Appendix*, Table S1). This dataset (47) was used in one previous publication (31); however, this previous study did not use any of the measurements from the follow-up surveys that were administered as part of the current study.

### Measures.

#### Initial desire (at the event).

In studies 1 and 2, we determined initial desire by averaging participants’ responses to the following post speed date survey items: “I am likely to say ‘yes’ to my interaction partner,” “I really liked my interaction partner,” and “I was sexually attracted to my interaction partner” (NSDS I: Cronbach’s α = 0.88; NSDS II: Cronbach’s α = 0.87), all measured on a nine-point scale (1 = strongly disagree to 9 = strongly agree).

In study 3, we determined initial desire by averaging participants’ responses to the following post speed date survey items: “How interested are you in this date romantically?” and “How interested are you in this date sexually?” (*r* = 0.84, *P* < 0.001) both measured on a seven-point scale (1 = not at all to 7 = very much).

#### Romantic initiation and evaluations (after the event).

All outcome variables, except for contact initiation, were completed on each follow-up survey. See *SI Appendix*, Table S15 for a summary of the number of yes and no responses for each dichotomous item across the studies.

##### Contact initiation (dichotomous).

In studies 1 and 2, we determined whether the participant initiated contact using the time stamps of the messages that participants sent to each other (nearly always in the few days following the event) via the online messaging system. Participants were coded as 1 = yes, if they initiated the first contact with their match, and 0 = no, if 1) their match initiated the first contact or 2) neither they nor their match contacted each other.

In study 3, we determined contact initiation using participants’ responses to the following item on the first weekly follow-up survey (to parallel studies 1 and 2): “Who initiated the very first contact between you and [this match]?” (response options: “I did” vs. “They did”). Because participants were only asked this item if they reported contact with their match, to parallel studies 1 and 2, we recoded this variable as 1 = yes, if respondents reported that they initiated contact, and 0 = no, if 1) they reported that their match initiated contact or 2) they indicated they did not have contact with their match that week.

##### Hanging out or corresponding (dichotomous).

In study 1, we determined hanging out or corresponding from the item, “Have you hung out with [match name] in person OR corresponded with [match name] not in person (email, IM, phone, etc.)?” since the last time they completed a survey. In study 2, this variable was determined from two items (which produced two independent estimates): “Have you hung out with [match name] in person?” and “Have you had any sort of correspondence with [match name] (email, IM, phone, texting, etc.)?” In study 3, the item was, “Have you and [this match] had interactions of any kind (electronically or in person) this last week?”

##### Later romantic interest (dichotomous).

In studies 1 and 2, we determined later romantic interest from participants’ responses to the following item: “What is the current status of your relationship with [match name]?” We recoded this variable as 0 for no romantic interest if participants selected "no relationship", "acquaintances without romantic potential", or "friends without romantic potential", or as 1 for has romantic interest if participants selected "acquaintances with romantic potential", "friends with romantic potential", "dating casually", or "dating seriously". In study 3, later romantic interest was assessed on a continuous scale (detailed in the next section).

##### Later romantic interest (continuous).

The follow-up surveys in studies 1 and 2 did not contain a continuous measure of romantic interest that all participants were eligible to complete (but, as described above, all participants were eligible to complete the dichotomous measure). In study 3, later romantic interest was the average of participants’ responses to the following items: 1) “How interested are you in [this match] sexually?” and 2) “How interested are you in [this match] romantically?” (*r =* 0.91, *P* < 0.001), both measured on a seven-point scale (1 = not at all to 7 = very much).

##### Desire to know match better (continuous).

In studies 1 and 2, the item was, “I am eager to get to know [match name] better,” measured on a seven-point scale (1 = strongly disagree to 7 = strongly agree). We did not assess this measure in study 3.

##### Physical attractiveness (continuous).

In studies 1 and 2, perception of the match’s physical attractiveness was the average of the following items: “I think this person is physically attractive” and “I think this person is sexy/hot” (study 1: *r* = 0.87, *P* < 0.001; study 2: *r* = 0.85, *P* < 0.001), both measured on a nine-point scale (1 = not at all to 9 = extremely). We did not assess this measure in study 3.

##### Perceived attraction from match (continuous).

In studies 1 and 2, perceived attraction from the match was the average of the following items: “I think that [match name] is sexually attracted to me” and “I think that [match name] is romantically interested in me” (study: 1 *r* = 0.87, *P* < 0.001; study 2: *r* = 0.83, *P* < 0.001). In study 3, the items were, “How interested do you think [this match] is in you sexually?” and “How interested do you think [this match] is in you romantically?” (*r* = 0.88, *P* < 0.001). All items were answered on a seven-point scale (1 = not at all to 7 = very much).

### Data Analysis.

#### Partitioning of initial desire via the SRM.

In each study, we used the SRM (7, 44) to divide initial romantic desire into partner effects (how popular each participant’s date was—that is, how much romantic interest each participant’s date received from all other daters, minus the grand mean for the date’s speed-dating event), actor effects (how much the participant was romantically interested in all dates, minus the grand mean for the participant’s speed-dating event), and relationship effects (how much the participant was uniquely romantically interested in each date, above and beyond actor and partner effects, and minus the grand mean for the participant’s speed-dating event; Fig. 1). For the men-only event, we used the round-robin formula for calculating these effects, as described in Kenny (44). For each study, we calculated one “primary” set of partner, actor, and relationship effects based on the survey answers of respondents (who we designate as “As”; Fig. 1), and a “secondary” set of partner, actor, and relationship effects based on the survey answers of the respondents’ dates (who we designate as “Bs”). We performed these calculations across the responses of all speed daters in each study, including those who later matched and those who did not. We examined each of these six variables as individual, unique predictors in separate regression analyses. That is, we separately examined how each of the six IVs predicted each of the six dependent variables (DVs), one at a time.

#### Multilevel regression analyses.

We used multilevel regression to assess whether each IV predicted each outcome variable, one at a time for each follow-up outcome in each study. We used multilevel logistic regression analyses for dichotomous (yes/no) outcome variables, and used multilevel continuous regression analyses for continuous outcome variables. All multilevel models contained the following random effects: participant ID, partner ID, participant ID nested within partner ID, and partner ID nested within participant ID. These random effects were used to account for repeated measurements (i.e., each follow-up wave) across participants and partners, repeated participant responses across different partners, and repeated ratings of partners across different participants. The SRM IVs were standardized for both the logistic and continuous regression analyses. To obtain beta weights from the continuous regression analyses, the continuous outcome variables were also standardized. All data analysis was done in R programming (45), using the glmer function (for logistic regression) and the lmer function (for continuous regression) from the lme4 package (46).

#### Meta-analysis.

We assessed the average association between the six initial desire variables and each follow-up outcome by performing a meta-analysis of the comparable analyses across the different studies. Following Park et al. (43), we calculated each meta-analytic effect by weighting each logit (for logistic regressions) and each β (for continuous regression) by the inverse of its variance. We did this so that more-precise estimates would be more influential in determining the overall estimates. We calculated the meta-analytic SE for each effect by taking the square root of the reciprocal of the sum of the weights. We conducted hypothesis tests by dividing each meta-analytic estimate by the meta-analytic SE, yielding a z statistic. We performed all calculations for the logistic regression analyses on the logit scale, but, for presentation, we exponentiated the meta-analyzed estimates, SEs, and CIs to convert to OR*s*.

#### Moderation by gender.

We tested each analysis for moderation by gender (note that we assessed participants’ gender identity rather than biological sex). For more information on our approach to these analyses, see *SI Appendix*, Note 6.

#### Sensitivity analyses.

We repeated each of the original analyses 1) with random slopes for each SRM predictor included in each model, 2) with all six SRM predictors included as simultaneous predictors in each model, and 3) with separate analyses for initial romantic liking and initial sexual desire. For more information on our approach to these analyses, see *SI Appendix*, Note 7.

## Supplementary Material

Supplementary File

## Data Availability

Anonymized Microsoft Excel Files have been deposited in Open Science Framework (10.17605/OSF.IO/3ZYN6) (47).
