# Supplementary material for: Initial impressions of compatibility and mate value predict later dating and romantic interest
Source: Proc Natl Acad Sci U S A. 2022 Nov 2;119(45):e2206925119. doi: 10.1073/pnas.2206925119 (PMC9659375; doi:10.1073/pnas.2206925119)
Supplement: Supplementary File [file pnas.2206925119.sapp.pdf]

PNAS

Supplementary Information for
Initial Impressions of Compatibility and Mate Value Predict Later Dating and Romantic Interest

Alexander Baxter[1-2*], Jessica A. Maxwell[3], Karen L. Bales[1-2, 4], Eli J. Finkel[5], Emily A. Impett[6], & Paul W. Eastwick[2]

* Corresponding Author: Alexander Baxter
Email: axbaxter@ucdavis.edu

This PDF file includes:

Supplementary text (Supplementary Notes 1-7
Supplementary model equations (Equations 1-5)
Supplementary figures S1-S4
Supplementary Tables S1-S15
SI References

Other supplementary materials for this manuscript include the following:

The additional materials listed below are available on an OSF page::
https://osf.io/3zyn6/?view_only=6fddb1a6f1e94abf9a04fe68c99c1218

Pre-registrations of Analysis Plan for Study 2 and Study 3 (Word Documents)
Pre-registrations of Additional Sensitivity Analyses (Word Documents)
Code for Original Analyses (R Markdown File)
Code for Gender Moderation Analyses (R Markdown File)
Code for Sensitivity Analyses (R Markdown Files)
Data Files for Study 1, Study 2, and Study 3 (CSV files)
Example Calculations for Meta-Analyses (Excel File)

Supplementary Notes

Supplementary Note 1

Rationale for Analyses on Perceived Attraction from Match

In addition to analyzing relationship initiation behaviors and later romantic evaluations, we also explored whether peoples' perceptions of how much their speed-dating matches desired them (after the event) were associated with primary and secondary partner, actor, and relationship effects in initial desire. This variable is present throughout the process of important for relationship initiation because without a sense of reciprocated desire, people are less likely to open up to a potential partner for fear of rejection (see 1). Perceived desire can also spark desire for a potential partner, even if a person did not initially like a potential partner (2); in other words, people like to be liked. We highlight this variable separately from the other outcomes because it involves an extra degree of meta-cognition that the other variables did not. For example, in all the other outcome variables that we considered, people reported on what *they* did (e.g., hanging out, initiating contact) or what *they* felt (e.g., desire to know a match better, physical attraction for a match). In these cases, people's reports were determined by (a) what they actually did or actually felt and (b) their ability to accurately remember and/or identify those activities or feelings (and we presume that people are relatively accurate at identifying their own internal states). However, when reporting on perceived desire from a match, people reported on what *they* thought *their match* felt. In this case, their reports were based on (a) the actual desire that their match emitted (intentionally or not) and (b) their ability to accurately detect that desire (see 3). This point is critical, as another person's desire may not be easily detected, especially at the start of a relationship when people are more likely to guard their feelings (see 1) or when potential partners are meeting for the first time. Moreover, people's own desire can sometimes inflate the desire they perceive (4, 5). When this happens, it can create a self-fulfilling prophecy: Because a person desires a partner (and because they think their desire is reciprocated), they are especially likely to pursue a relationship with that partner. These behaviors and desires could also kindle desire in the partner, even if the partner did not initially desire that person (see 6). Hence, perceived desire can motivate people to pursue a relationship with a partner, regardless of if the perception is accurate or not, and may be an important variable in relationship initiation.

In the analyses of the primary SRM variables predicting later perceived desire from match, we tested for *projection*. In other words, we assessed whether perceived desire from matches (after speed-dating) was associated with participants' own levels of initial desire for those matches. We hypothesized that projection would occur, especially for primary relationship effects. In the analyses of the secondary SRM variables, we tested for *accuracy*; in other words, we assessed whether perceived desire from matches aligned with the initial desire that matches reported (which presumably lingered). We hypothesized that the secondary SRM effects would be positively correlated with perceived desire from match, especially for secondary relationship effects.

Supplementary Note 2

Commentary on Analyses for Perceived Desire from Match

To our knowledge, this study is the first to employ the SRM to investigate whether lingering romantic impressions predict perceived desire from potential partners. We found that perceived desire from matches was modestly associated with secondary partner and secondary relationships (see Table 4, and Figures 4A and 4B). In other words, “Harry” would be most likely to perceive that “Sally” desired him if (a) Harry was consensually desirable (secondary partner effects) and (b) Sally uniquely desired Harry (secondary relationship effects). These results suggest that people (a) have some sense of their own desirability to others and (b) can detect when specific people desire them. Congruent with other studies of perceived attraction during initial encounters (see, for example, 3, 7), the effect sizes were modest. This may suggest that participants were only moderately aware of a match’s true desire for them (or lack thereof). However, we note that the secondary SRM analyses only assessed match’s initial desire for a participant *at* speed-dating and the participant’s perceived desire from that match *after* speed-dating. Hence, if a match’s initial desire for a potential partner changed after speed-dating, this could also explain why perceived desire (measured *after* speed-dating) was only modestly associated by the match’s *initial* desire (measured *during* speed-dating). Future studies should explore how initial and concurrent desire are perceived by potential partners over time.

Although we expected that people might project their own romantic desire for matches onto the desire they perceived from those matches (8), we found no associations between any of the primary SRM effects and perceived desire from match. In other words, the desire that Harry perceived from Sally was not associated with (a) Sally’s consensual desirability, (b) Harry’s general desirousness, or (c) Harry’s unique liking for Sally. These null findings could suggest that the “projection of desire” phenomenon does not occur during the initial phases of relationship initiation. For example, during initial interactions with a potential partner, people potential partner’s interest before they invest in pursuing a relationship. However, as people get to know each other and the risk of rejection decreases, their own desire for a partner (or potential partner) may become increasingly intertwined with their perception of that partner’s desire for them (see, for example, 6, 8, 9).

Supplementary Note 3

Results of Analyses testing for Moderation by Gender

A summary of each analysis and the meta-analyzed effect sizes is presented in Supplementary Tables S5A-S5F and S6A-S6F. Across the meta-analyses performed, only four associations were moderated by gender, as indicated by a significant interaction with gender (see Supplementary Table S7). These included: (1) primary partner effects predicting hanging out or corresponding (Interaction OR = 0.79, $p < .001$); (2) primary partner effects predicting romantic interest (on a binary scale) (Interaction OR = 0.77, $p = .036$); (3) primary actor effects predicting contact initiation (Interaction OR = 0.27, $p = .014$); and (4) secondary partner effects predicting hanging out or corresponding (Interaction OR = 1.22, $p = .011$).

Simple effect analyses showed that, for men, the primary partner effect (how consensually desirable their match was at speed-dating) was a stronger predictor of hanging out or corresponding (meta-analytic OR = 1.52, $p < .001$) and romantic interest on a binary scale (OR = 2.87, p < .001) than it was for women (hanging out and corresponding: OR = 0.94, $p = .47$; binary romantic interest: OR = 2.12, $p < .001$). For women, the primary actor effect (how generally desirous a participant was at speed-dating) was a stronger predictor of contact initiation (OR = 1.78, $p = .093$) than it was for men (OR = 0.88, $p = .35$), although we note that the associations did not achieve significance for either group. The secondary partner effect (how consensually desirable a participant was at speed-dating) was also a better predictor of hanging out or corresponding for women (OR = 1.46, $p < .001$) than it was for men (OR = 0.99, $p = .95$).

The meta-analyses also showed gender differences in many of the romantic outcomes considered. Compared to men (coded as 0), women (coded as 1) were less likely to initiate contact with their matches (meta-analytic OR 0.34-0.38), report romantic interest (on a binary scale; meta-analytic OR 0.58-0.61), desire to know a match better (meta-analytic β from -0.12 to -0.14), rate a match as physically attractive (meta-analytic β ranged from -0.23 to -0.25), and to perceive attraction from a match (meta-analytic β ranged from -0.23 to -0.24). In other words, on average, men performed more relationship initiation behaviors than women and rated their matches higher on romantic evaluations. There were no meta-analytic gender differences in hanging out or corresponding.

Supplementary Note 4

Summary and Commentary on the Results of the Sensitivity Analyses

Sensitivity Analysis 1: Random Slopes

The results of the first set of sensitivity analyses in which random slopes for the SRM variable were added to each model (within participant ID and partner ID) were similar to the results of the original analyses, although in some cases the 95% confidence intervals were slightly increased for some of the analyses (see Supplementary Figures S2A-S2C, Supplementary Tables S9A-S9F and S10A-S10F). At the meta-analytic level, there were only two results that were significant in the original analyses but that were not significant in this set of sensitivity analyses, including secondary actor effects predicting later romantic interest on a binary scale (OR = 0.78, p = .121; Original Model: OR = 0.72, p = .011; see Supplementary Table S9E) and secondary relationship effects predicting later romantic interest on a binary scale (OR= 1.34, p = .059; Original Model: OR = 1.24, p = .046; see Supplementary Table S9F).

Sensitivity Analysis 2: Maximal Models

The results of the second set of sensitivity analyses in which all six SRM variables were added as simultaneous predictors of each relationship initiation outcome were also similar to the results of the original analyses (see Supplementary Figures S3A-S3C, Supplementary Tables S11A-S11C and S12A-S12D), although in some cases, the effect sizes for the primary SRM effects were slightly larger and the effect sizes for the secondary SRM effects were slightly smaller compared to the original analyses. At the meta-analytic level, there were four effect sizes that were significant in the original analyses that were not significant in this set of sensitivity analyses, including primary actor effects predicting hanging out or corresponding (OR = 0.95, p = .492; Original Model: OR = 0.84, p = .019; see Supplementary Table S11B), secondary actor effects predicting later romantic interest on a binary scale (OR = 0.93, p = .559; Original Model: OR = 0.72, p = .011; see Supplementary Table S11C) and desiring to know a match better (β = -0.01, p = .703; Original Model: β = -0.07, p = .014; see Supplementary Table S12B), and secondary relationship effects predicting later romantic interest on a binary scale (OR = 1.18, p = .119; Original Model: OR = 1.24, p = .046; see Supplementary Table S11C). There were also two meta-analytic effects that were not significant in the original analyses but that were significant in these sensitivity analyses, including primary actor effects predicting later romantic interest on a binary scale (OR = 1.63, p < .001; Original Model: OR = 1.13, p = .347) and perceived attraction from match (β = 0.11, p = .004; Original Model: β = 0.05, p = .147).

Sensitivity Analysis 3: Separate Analyses for Romantic and Sexual Desire

The results of the third set of sensitivity analyses showed that when romantic liking and sexual desire were considered separately, the effect sizes for the SRM components of initial sexual desire were slightly stronger than the effect sizes for initial romantic liking (see Supplementary Figures S4A-S4C, Supplementary Tables S13A-S13F and S14A-S14F). For initial sexual desire, all meta-analytic effect sizes that were significant in the original analyses were also significant when initial sexual desire was considered separately. There were also two meta-analytic effect sizes that were not significant in the original analyses but that were significant when initial sexual desire was considered separately, including primary actor effects predicting later romantic interest on a binary scale (OR = 1.35, p = .017; Original Model: OR = 1.13, p = .347; see Supplementary Table S13B) and primary partner effects predicting perceived

sexual attraction from match (β = 0.05, *p* = .026; Original Model: β = 0.03, *p* = .210; see Supplementary Table S14A).

For initial romantic liking, there were eight effect sizes that were significant in the original analyses but that were not significant when initial romantic liking was considered separately, including primary partner effects predicting hanging out or corresponding (OR = 1.09, *p* = .223; Original Model: OR = 1.15, *p* = .032; see Supplementary Table S13A), primary actor effects predicting hanging out or corresponding (OR = 0.88, *p* = .084; Original Model: OR = 0.84, *p* = .019; see Supplementary Table S13B), secondary partner effects predicting contact initiation (OR = 0.79, *p* = .056; Original Model: OR = 0.61, *p* < .001) and hanging out or corresponding (OR = 1.12, *p* = .125; Original Model: OR = 1.20, *p* = .018; see Supplementary Table S13D), secondary actor effects predicting desiring to know a match better (β = -0.01, *p* = .803; Original Model: β = -0.07, *p* = .014; see Supplementary Table S14E), and secondary relationship effects predicting later romantic interest on a binary scale (OR = 1.06, *p* = .610; Original Model: OR = 1.24, *p* = .046; see Supplementary Table S13F), perceived *romantic* attraction from match (β = 0.02, *p* = .308; Original Model: β = 0.08, *p* < .001), or perceived *sexual* attraction from match (β = 0.02, *p* = .323; Original Model: β = 0.08, *p* < .001; see Supplementary Table S14F). There were also two meta-analytic effect sizes that were not originally significant but that were significant when initial romantic liking was considered separately, including secondary relationship effects predicting desiring to know a match better (β = 0.06, *p* = .016; Original Model: β = 0.04, *p* = .124; see Supplementary Table S14F) and primary actor effects predicting perceived sexual attraction from match (β = 0.08, *p* = .030; Original Model = 0.05, *p* = .147; see Supplementary Table S14B).

We note that one interesting result of this sensitivity analysis was that although secondary *partner* effects in both initial romantic liking and initial sexual desire were positively associated with perceived attraction from the match (both romantic and sexual attraction; β ranged from 0.11-0.22, p < .002; see Supplementary Table S14D), for secondary *relationship* effects, only secondary relationship effects in initial *sexual* desire predicted later perceived attraction from match (both romantic and sexual attraction; β ranged from 0.10-0.13, *p* < .001; see Supplementary Table S14F); secondary relationship effects in initial *romantic* liking did not predict either measure of later perceived attraction (β was 0.02 in both cases, *p* > .30; see Supplementary Table S14F, Supplementary Figure S4C). These results suggest that while people have some sense of how romantically and sexually appealing they are on a general level (secondary partner effects) and how *sexually* appealing they are to specific potential partners (secondary relationship effects in sexual desire), *romantic* liking from specific potential partners may be harder to discern (potentially because first impressions change and update as people continue to interact). However, an important caveat to these interpretations is that the survey items in these studies were not necessarily designed to test differences between romantic and sexual desire, and further research would be needed to explore this interpretation. Further, we note that the effect sizes for perceived attraction from the math (either romantic or sexual) were relatively small (the highest value was β = 0.13), suggesting that peoples' ability to detect either type of attraction from potential partners is relatively modest.

Overall Commentary and Consistency of Original Analyses

Overall, the meta-analytic effect sizes in each sensitivity analysis were generally in the same direction as the original analyses and were approximately the same strength. We conclude

that the results of the original analyses were relatively robust across all three sensitivity analyses. Sensitivity analysis 1 showed that there were minimal effects of individual-level variation in how each SRM pathway affected (or did not affect) relationship initiation. Sensitivity analysis 2 showed that although the primary SRM effects were slightly stronger than the secondary SRM effects when considered at the same time, the overall pattern of results did not change, suggesting that a person's initial desire for a potential partner and the potential partner's initial desire for that person both play important roles in the relationship initiation process (but with different contributions from the SRM components that underlie each variable). Likewise, sensitivity analysis 3 showed that although initial sexual desire was a somewhat stronger predictor of relationship initiation compared to initial romantic liking, the overall pattern of results did not change, suggesting that both factors play an important role in relationship initiation (with similar contributions from the SRM components that underlie each variable).

Supplementary Note 5

Calculations for Tabulating Average Effect Sizes on Pearson r Scale

The current study involved both multilevel logistic regression analyses and multilevel continuous analyses. For the purposes of hypothesis testing, all meta-analyses were performed separately for the dichotomous outcomes (with effect sizes on the odds-ratio scale) and continuous outcomes (with effect sizes on a beta-weight scale) using inverse variance weights, as described in (10) (see also the Data Analysis section of the main text). This method was appropriate for determining averaged effect sizes and conducting null hypothesis tests to determine if the average effect size for each relationship initiation variable differed from 0 (for the continuous analyses) or 1 (for the dichotomous outcome variables that were on the OR scale) and yielded seven effect sizes (for each outcome variable) for each of the six primary and secondary SRM independent variables. For descriptive purposes, we also tabulated the average effect size for primary partner and primary relationship effects across studies and relationship initiation outcome variables.

To do this, we first determined the unweighted average effect size (on the logit scale for the dichotomous outcomes and on the beta-weight scale for the continuous outcomes) across studies for each of the six relationship initiation outcome variables (separately for primary partner and primary relationship effects; we did not include later perceived attraction from match in these calculations). We then converted each average logit to the Pearson *r* scale using the *logoddsratio_to_r* function from the *effectsize* R package (11). We then averaged the six effect sizes related to relationship initiation (i.e., the three effect sizes that were converted from OR to Pearson *r* and the three effect sizes that were on the beta-weight scale) to obtain a single average effect size on the Pearson *r* scale for primary partner effects and primary relationship effects.

Supplementary Note 6

Data Analysis Methods for Analyses Testing for Moderation by Gender

To test for moderation by gender, we repeated each multilevel logistic and multilevel continuous regression with the following variables included as predictors in the model: an SRM independent variable (primary or secondary actor, partner, or relationship effects; only one of these variables was included in the model at a time), gender (0 = men, 1 = women) and the interaction between gender and the SRM independent variable. The independent and dependent variables were standardized in all analyses, except for the dichotomous dependent variables in the logistic regressions. As in the main analyses (see Supplementary Equation 1), all multilevel models contained the following random effects: participant ID, partner ID, participant ID nested within partner ID, and partner ID nested within participant ID (for more details, see the "Data Analysis" section of the primary manuscript). See Supplementary Equation 2 for a summary of these models in formula notation.

In some cases (see Supplementary Tables S5B and S5D), when all four of these random effects were included, the models produced implausibly low standard error estimates (in turn producing implausibly large *Z* test statistics, ranging from 300-1,000 for each coefficient). When this happened, we repeated the analyses without the nested random effects (i.e., participant ID nested within partner ID and partner ID nested within participant ID). If needed, Target ID was also removed as a random factor. The results of the original analyses with all four random effects included are available upon request.

For each grouping of outcome variables, we determined the average association between each predictor (the six SRM variables, gender, and the interaction between gender and the SRM variable) and the outcomes tested by performing meta-analyses. Following Park, Young, Eastwick, Troisi and Streamer (10), we calculated each meta-analytic effect by weighting each logit (for logistic regressions) and each beta (for continuous regression) by the inverse of its variance. We did this so that more precise estimates would be more influential in determining the overall estimates. We calculated the meta-analytic standard error for each effect by taking the square root of the reciprocal of the sum of the weights. We conducted hypothesis tests by dividing each meta-analytic estimate by the meta-analytic standard error, yielding a z-statistic.

This approach generated 36 meta-analytic effect sizes for the SRM independent variable, 36 effect sizes for gender, and 36 effect sizes for the interaction between gender and the SRM independent variable. For the interaction coefficients that were significant in the meta-analysis, we performed follow-up simple effect analyses by assessing the relationship between the SRM variable and the outcome variable separately for men and women. In these analyses, the SRM independent variable was used as the sole predictor. The same four random effects used in the full model (see above) were also included in the model, and when needed we used the same procedure for removing the nested effects as described above. We then meta-analyzed the individual effect sizes for men and women separately, using the same meta-analytic approach (see above).

Supplementary Note 7

Data Analysis Methods for Sensitivity Analyses

We performed three sets of follow up sensitivity analyses to assess whether and how the results of the original analyses would change when accounting for (1) individual-level differences in how each SRM effect predicted relationship initiation, (2) interdependence between the primary and secondary SRM effects, or (3) possible differences in romantic and sexual desire. We did not repeat any of the analyses that tested for gender moderation.

Sensitivity Analysis 1: Random Slope Models

In the original analyses, we accounted for repeated measurements from repeated participants across repeated partners (speed-dating matches) in the follow up surveys by including random intercepts for participant ID, partner ID, participant ID nested in partner ID, and partner ID nested within participant ID. However, these original analyses did not account for the possibility of initial desire (and its various SRM components) having different effects across different participants and/or potential romantic partners. In this set of sensitivity analyses, we assessed how the original results would change when random slopes were used to account for this possibility.

To do this, we repeated all multilevel model analyses with two random slopes included for the SRM predictor in each model: One within participant ID (to account for different associations between the SRM predictor and the outcome variable across participants), and one within partner ID (to account for different associations between the SRM predictor and the outcome variable across different potential romantic partners). As in the original model, nested random intercepts were also included in the model for Participant ID within Partner ID and for Partner ID within Participant ID (random slopes were not included within these nested random effects). See Supplementary Equation 3 for a summary of these models in formula notation.

For these analyses, we calculated DF as n-1 (for the SRM variable used as the predictor)-4 (one for each random intercept)-2 (one for each random slope)-2 (one for each correlation between the random slope and its intercept)-1. As in the original analyses, we based n off the number of unique participant ID's in each primary SRM analysis and the number of unique partner ID's in each secondary SRM analysis.

In some cases (see Supplementary Tables S9A and S9F), when the four random effects and the two random slopes were included, the models produced implausibly high slope estimates and/or implausibly low standard error estimates (in turn producing implausibly large Z test statistics and/or extreme 95% confidence interval ranges). When this happened, we first repeated the analyses without the nested random effects (i.e., participant ID nested within partner ID and partner ID nested within participant ID). If needed, the random slope for the SRM variable within Target ID was also removed as a random slope (this was necessary for one analysis involving Study 2 Contact Initiation). If the model still produced implausible estimates, we removed the random slope estimates all together and used the four original random intercepts in the model (this was necessary for four of the analyses for Study 3 Contact Initiation). The results of the initial analyses with all four random effects and the two random slopes included are available upon request.

Sensitivity Analysis 2: Maximal Models

In the original analyses, we tested each primary and secondary SRM effect as an individual predictor of each outcome variable. These analyses did not account for the interdependence between the primary and secondary SRM components of initial desire. For example, across each speed-dating sample, primary and secondary relationship effects were positively correlated (Pearson *r* ranged from 0.08-0.15, $p < .001$; see Supplementary Table S8), indicating that unique initial desire tended to be reciprocated. Further, primary actor effects were negatively correlated with secondary partner effects, and secondary partner effects were negatively correlated with secondary actor effects (*r* ranged from 0.10-0.28, p < .001; see Supplementary Table S8), indicating that people who were generally desirous (unselective) were less generally desirable at speed-dating. Note, within each study, the correlation between primary actor effects and secondary partner effects was the exact same as the correlation between secondary actor effects and primary partner effects, as these two pairs of variables were reciprocals of each other because the data were analyzed in long format.

In this set of sensitivity analyses we accounted for these patterns of dependence between the primary and secondary SRM variables by including all six SRM variables as simultaneous predictors in each model (with separate analyses for each relationship initiation dependent variable). As in the original analyses, we included random intercepts in each model for participant ID, partner ID, participant ID within partner ID, and partner ID within participant ID (we did not include any random slopes in these models). It was not necessary to remove any random effects from any maximal model. See Supplementary Equation 4 for a summary of these models in formula notation.

For these analyses, we calculated DF as *n*-6 (one for each SRM variable predictor)-4 (one for each random intercept)-1. Because there were both primary and secondary SRM components in each model, we based *n* off the total number of unique participant ID’s in each analysis (in the original analyses, we based n off the number of unique participant ID’s in each primary SRM analysis and the number of unique partner ID’s in each secondary SRM analysis).

Sensitivity Analysis 3: Separate Analyses for Romantic and Sexual Desire

In the original analyses, initial desire was an average of participants’ responses to survey items (given at the speed-dating event) related to their romantic interest in their date (Studies 1-3), their sexual interest in their date (Studies 1-3), and how likely they were to say “yes” to having further contact with their date after the event if they “matched” (Studies 1-2). There were also two follow up measures that were originally an average of participants’ responses to items related to both romantic and sexual desire, including continuous later interest (in Study 3) and perceived attraction from match (in Studies 1-3). The purpose of these sensitivity analyses was to assess whether the overall pattern of results would change if initial romantic interest and initial sexual interest (and the underlying SRM components of each variable) were considered separately. We also considered romantic and sexual interest as separate outcome variables for the two relevant dependent variables.

To do this, we first determined the SRM components of initial romantic liking and initial sexual desire by separately applying the SRM calculations to each measure of initial desire. This yielded 12 separate variables (i.e., three primary and three secondary SRM components of initial

romantic liking, and three primary and three secondary components of initial sexual desire). We then repeated each original analysis using each of the 12 SRM components of initial romantic liking and initial sexual desire to predict each relationship initiation outcome assessed in the follow up surveys (with separate analyses for each SRM variable and each follow up variable). For Study 3, separate analyses were performed for the continuous measures of later romantic interest and later sexual interest (these measures were averaged in the original analyses). For Studies 1-3, separate analyses were performed for perceived romantic attraction from match and perceived sexual attraction from match (these measures were averaged in the original analyses). As in the original analyses, participant ID, partner ID, participant ID within partner ID, and partner ID within participant ID were included in each model as random effects. See Supplementary Equation 5 for a summary of these models in formula notation.

We calculated the DF for each model as *n*-1 (for the SRM variable used as the predictor)-4 (one for each random intercept)-1. As in the original analyses, we based *n* off the number of unique participant ID's in each primary SRM analysis and the number of unique partner ID's in each secondary SRM analysis.

In some cases (see Supplementary Tables S13A and S13F), when all four random effects were included, the models produced implausibly high slope estimates and/or implausibly low standard error estimates (in turn producing implausibly large Z test statistics and/or extreme 95% confidence interval ranges). When this happened, we first repeated the analyses without the nested random effects (i.e., participant ID nested within partner ID and partner ID nested within participant ID were removed). If needed, partner ID (for the primary SRM analyses) or participant ID (for the secondary SRM analyses) was also removed as a random intercept (this was necessary for all six analyses for the Study 3 Contact Initiation involving the SRM components of initial *sexual* desire; see Supplementary Tables S13A-S13F). The results of the initial analyses with all four random effects are available upon request.

We note that for Study 2 and Study 3, there were slightly fewer participants used in each sensitivity analysis compared to the original analyses because some participants were missing data for either initial romantic liking or initial sexual desire (i.e., these participants reported on either their initial romantic liking or their initial sexual desire for their speed-date partner but did not report on both), and in order to correctly perform the SRM calculations, it was necessary to exclude these participants. E.g., if participants did not report on initial sexual desire for at least one of their speed-dating partners, we excluded all of that participant's reports of sexual desire towards all their dates and the reports of sexual desire that the participant received from all their dates when calculating the SRM effects in initial sexual desire (the same procedure was applied for participants who were missing at least one report of initial romantic liking for at least one speed-dating partner). See Supplementary Table S1 for a summary of how many participants were used to calculate the SRM effects in initial romantic and initial sexual desire in each study.

There were also some responses that were dropped from the Study 3 analyses of later romantic interest (n = 3 responses) and later sexual interest (n = 221 responses) and from the analyses of perceived romantic attraction from match (Study 1: n = 0 responses; Study 2: n = 1 response; Study 3: n = 1 response) and perceived sexual attraction from match (Study 1: n = 0 responses; Study 2: n = 1 response; Study 3: n = 226 responses) due to participants completing only one of the follow up survey items related to later romantic or sexual interest/later perceived

romantic or sexual attraction. We note that the reason these participants were dropped in these follow up analyses but were not dropped from the original analyses was because in the original analyses we handled cases of partially-missing data (i.e., participants who completed one item related to romantic or sexual interest but not both) by averaging the responses that were available (e.g., if a participant did not report on later sexual interest in their date, we used their response for later romantic interest to determine “continuous later interest”; if both responses were available, we used the average). We also used this approach to handle missing data for initial desire in the original analyses (e.g., if a participant did not report on initial sexual desire, in Studies 1-2 we used the average of initial romantic liking and likelihood to saying “yes” to matching to determine “average initial desire”, and in Study 3 we used initial romantic liking to determine “average initial desire”). Hence, in the original analyses, participants were only dropped from analyses if they were missing responses to both the items related to romantic and sexual desire for the various measures of initial or later desire.

Meta-Analyses

As in the original analyses, for each set of sensitivity analyses, we used a meta-analytic method based on Park, Young, Eastwick, Troisi and Streamer (10) to determine the average effect of each SRM variable on each outcome variable across studies. For more information, see the Data Analysis section of the main text (this same procedure was used for the gender moderation analyses and is also described in Supplementary Note 6).

Supplementary Model Equations

This section describes the different models used for the original analyses (Supplementary Equation 1), the gender moderation analyses (Supplementary Equation 2), and the three sensitivity analyses performed (Supplementary Equations 3-5). Each equation is written using a notation style similar to the one used for analyzing multi-level models in the R package *lme4* (13). See each equation caption for a full description of each model equation.

Supplementary Equation 1

Models for Original Analyses

Outcome $_j$ ~
SRM Variable $_k$ +
(1 | Participant ID) +
(1 | Partner ID) +
(1 | Participant ID : Partner ID) +
(1 | Partner ID : Participant ID)

Supplementary Equation 1. Outcome *j* was one of the three dichotomous relationship initiation variables (i.e., Contact Initiation, Hanging Out or Corresponding, Binary Later Romantic Interest) or one of the four continuous relationship initiation variables (i.e., Later Interest, Desire to Know Better, Physical Attractiveness, or Perceived Attraction from Match) assessed in the follow up study and was used as the dependent variable. Multi-level logistic regression models were used for the dichotomous variables and multi-level continuous regression models were used for the continuous variables (continuous outcome variables were z-scored for analyses). SRM Variable *k* was one of the three primary or three secondary SRM components of initial desire and was used as the independent variable and was z-scored for analyses. Separate analyses were performed for each of the 7 outcome variables, each of the 6 SRM independent variables, and each study. In each model, the variables indicated in parentheses were included as random intercepts (variables joined by colons indicate nested random intercepts). There were no random slopes included in the models.

Supplementary Equation 2

Models for Gender Moderation Analyses

Outcome $_j$ ~
SRM Variable $_k$ +
Gender +
SRM Variable $_k$ * Gender +
(1 | Participant ID) +
(1 | Partner ID) +
(1 | Participant ID : Partner ID) +
(1 | Partner ID : Participant ID)

Supplementary Equation 1. Outcome *j* was one of the three dichotomous relationship initiation variables (i.e., Contact Initiation, Hanging Out or Corresponding, Binary Later Romantic Interest) or one of the four continuous relationship initiation variables (i.e., Later Interest, Desire to Know Better, Physical Attractiveness, or Perceived Attraction from Match) assessed in the follow up study and was used as the dependent variable. Multi-level logistic regression models were used for the dichotomous variables and multi-level continuous regression models were used for the continuous variables (continuous outcome variables were z-scored for analyses). SRM Variable *k* was one of the three primary or three secondary SRM components of initial desire and was used as the independent variable and was z-scored for analyses. Gender was coded as 0 = female and 1 = male (this variable was z-scored for analyses). The interaction between SRM Variable *k* and Gender was also included in the model. Separate analyses were performed for each of the 7 outcome variables, each of the 6 SRM independent variables (with gender and the interaction between the SRM variable and gender included in each model), and each study. In each model, the variables indicated in parentheses were included as random intercepts (variables joined by colons indicate nested random intercepts). There were no random slopes included in the models.

Supplementary Equation 3

Models with Random Slopes Included (Sensitivity Analysis 1).

Outcome $_j$ ~
SRM Variable $_k$ +
(1 + SRM Variable $_k$ | Participant ID) +
(1 + SRM Variable $_k$ | Partner ID) +
(1 | Participant ID : Partner ID) +
(1 | Partner ID : Participant ID)

Supplementary Equation 3. Outcome *j* was one of the three dichotomous relationship initiation variables (i.e., Contact Initiation, Hanging Out or Corresponding, Binary Later Romantic Interest) or one of the four continuous relationship initiation variables (i.e., Later Interest, Desire to Know Better, Physical Attractiveness, or Perceived Attraction from Match) assessed in the follow up study and was used as the dependent variable. Multi-level logistic regression models were used for the dichotomous variables and multi-level continuous regression models were used for the continuous variables (continuous outcome variables were z-scored for analyses). SRM Variable *k* was one of the three primary or three secondary SRM components of initial desire and was used as the independent variable and was z-scored for analyses. Separate analyses were performed for each of the 7 outcome variables, each of the 6 SRM independent variables, and each study. In each model, the variables indicated in parentheses were included as random effects. Variables on the left side of the bar represent random slopes and variables on the right side of the bar represent random intercepts (variables joined by colons indicate nested random intercepts).

Supplementary Equation 4

Maximal Multilevel Models with All Six SRM Variables Included as Simultaneous Predictors (Sensitivity Analysis 2)

Outcome $_j$ ~
Primary Partner Effects +
Primary Actor Effects +
Primary Relationship Effects +
Secondary Partner Effects +
Secondary Actor Effects +
Secondary Relationship Effects +
(1 | Participant ID) +
(1 | Partner ID) +
(1 | Participant ID : Partner ID) +
(1 | Partner ID : Participant ID)

Supplementary Equation 4. Outcome *j* was one of the three dichotomous relationship initiation variables (i.e., Contact Initiation, Hanging Out or Corresponding, Binary Later Romantic Interest) or one of the four continuous relationship initiation variables (i.e., Later Interest, Desire to Know Better, Physical Attractiveness, or Perceived Attraction from Match) assessed in the follow up study and was used as the dependent variable. Multi-level logistic regression models were used for the dichotomous variables and multi-level continuous regression models were used for the continuous variables (continuous outcome variables were z-scored for analyses). Each of the six primary and secondary SRM variable was included as an independent variable in each model and were z-scored for analyses. Separate analyses were performed for each of the 7 outcome variables, each of the 6 SRM independent variables, and each study. In each model, the variables indicated in parentheses were included as random effects. Variables on the right side of the bar represent random intercepts (variables joined by colons indicate nested random intercepts). There were no random slopes included in the models.

Supplementary Equation 5

Multilevel Models for Romantic and Sexual Desire Separately (Sensitivity Analysis 3)

Outcome $_{\text{R or S}}$ ~
SRM Variable $_{\text{R or S}}$ +
(1 | Participant ID) +
(1 | Partner ID) +
(1 | Participant ID : Partner ID) +
(1 | Partner ID : Participant ID)

Supplementary Equation 5. Outcome *R or S* was one of the three dichotomous relationship initiation variables (i.e., Contact Initiation, Hanging Out or Corresponding, Binary Later Romantic Interest) or one of the five continuous relationship initiation variables (Later *Romantic* Interest, Later *Sexual* Desire, Desire to Know Better, Physical Attractiveness, Perceived *Romantic* Attraction from Match, Perceived *Sexual* Attraction from Match) assessed in the follow up study and was used as the dependent variable (note, in all other analyses there were only seven relationship initiation variables assessed, but in this sensitivity analysis we considered romantic and sexual desire separately for two outcome variables). Multi-level logistic regression models were used for the dichotomous variables and multi-level continuous regression models were used for the continuous variables (continuous outcome variables were z-scored for analyses). SRM Variable *R or S* was one of the primary or secondary SRM components of initial romantic liking or initial sexual desire and was used as the independent variable and was z-scored for analyses. Separate analyses were performed for each of the 9 outcome variables, each of the 12 SRM independent variables, and each study. In each model, the variables indicated in parentheses were included as random effects. Variables on the right side of the bar represent random intercepts (variables joined by colons indicate nested random intercepts). There were no random slopes included in the models.

Supplementary Figures

Supplementary Figure S1A

Participation and Attrition in the Study 1 Follow Up.

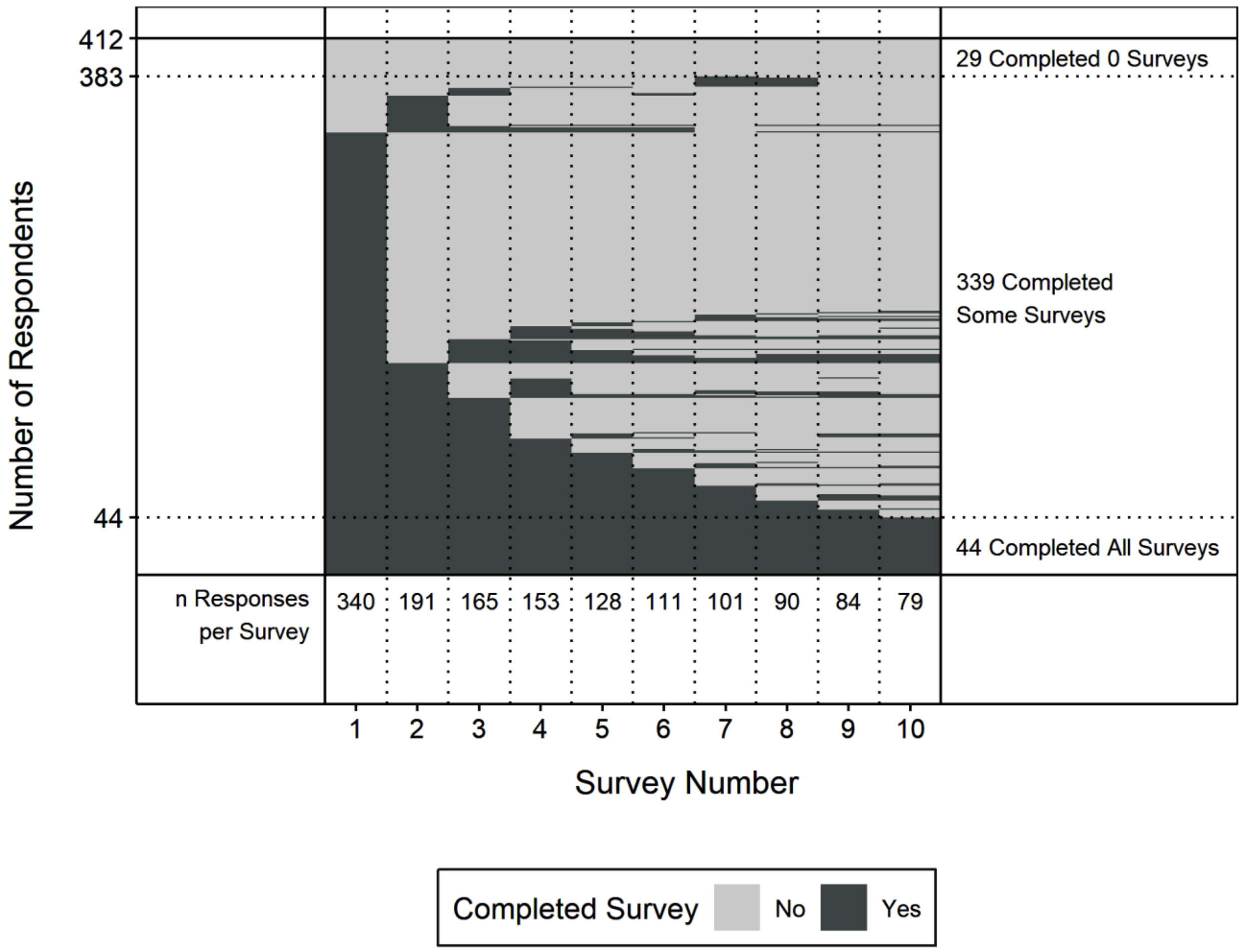

See below for combined figure caption for Supplementary Figures S1A-S1C.

Supplementary Figure S1B

Participation and Attrition in the Study 2 Follow Up.

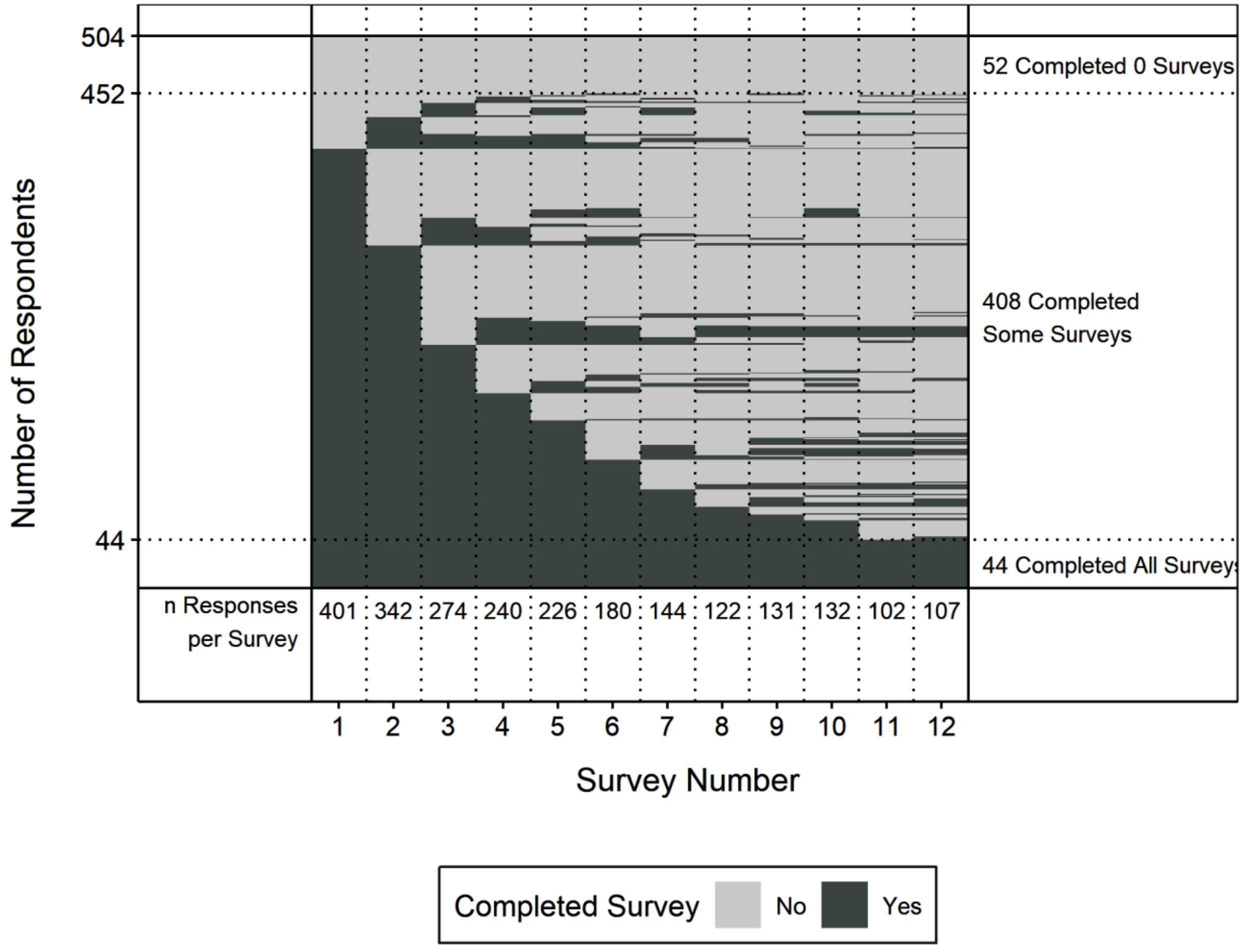

See below for combined figure caption for Supplementary Figures S1A-S1C.

Supplementary Figure S1C

Participation and Attrition in the Study 3 Follow Up.

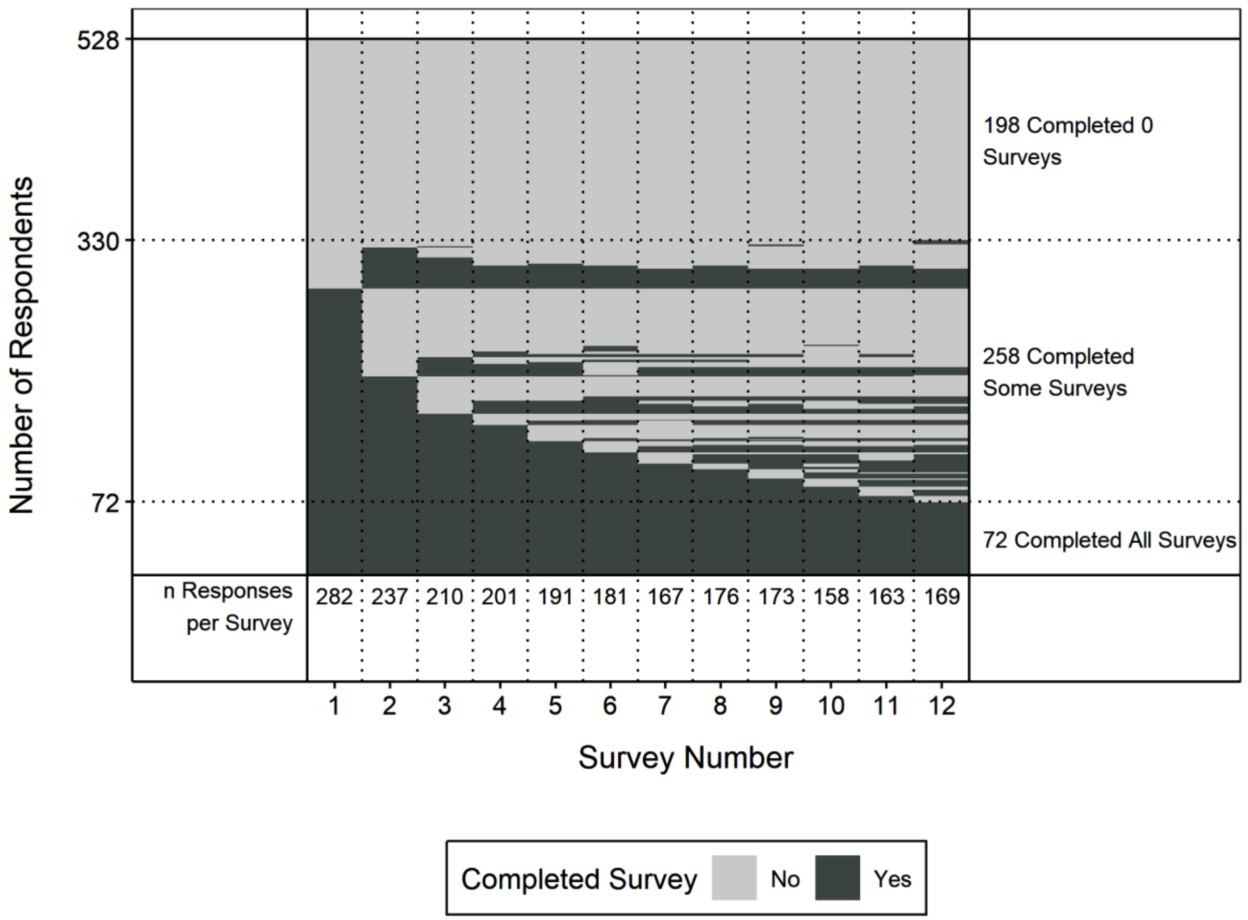

Supplementary Figure S1A-S1C. The figures show how many follow-up surveys (represented by dark grey tiles) that each participant completed for each of their speed-dating matches (represented on the y axis) completed across each follow-up survey (represented by columns along the x axis) Study 1 (Figure S1A), Study 2 (Figure S1B), and Study 3 (Figure S1C). Each dark grey tile represents a participant that completed a survey regarding their match at the given time point and light grey tiles represent participants that did not complete the survey. Respondents are organized from most to least surveys completed along the y axis. The total number of surveys completed at each time point (n Responses per Survey) is indicated underneath each column for each survey time point. The numbers on the right side of the figure label the total number of participants who completed all 10-12 surveys for a match, the number of participants who completed at least one but not all surveys for a match, and the number of participants who did not complete any surveys for a match.

We note that although it is possible that there may be survivorship bias present in the follow up survey responses (i.e., people are more likely to completely the follow up surveys for matches they are interested in / developing a relationship with), participants were surveyed concerning *all*

their matches at each follow-up time point, not just the ones in which they were still romantically interested in (participants were asked more detailed follow-up questions about matches they reported continued romantic desire towards, but we did not analyze any of these items in this study). In other words, participants still had the opportunity to report on matches even if they had low initial desire for them or low current romantic desire for them. For this reason, we assumed that data were missing (relatively) at random, and that participants' level of initial or current romantic desire towards their matches did not influence whether they chose to complete the follow up survey. Nonetheless, we ultimately do not know why people chose not to complete the follow up surveys, and future studies should consider ways to assess whether romantic interest in a potential partner is correlated with participation or attrition in the follow up data collection process.

Supplementary Figure S2A

Results of Sensitivity Analyses with Random Slopes Included for Logistic Regression Models

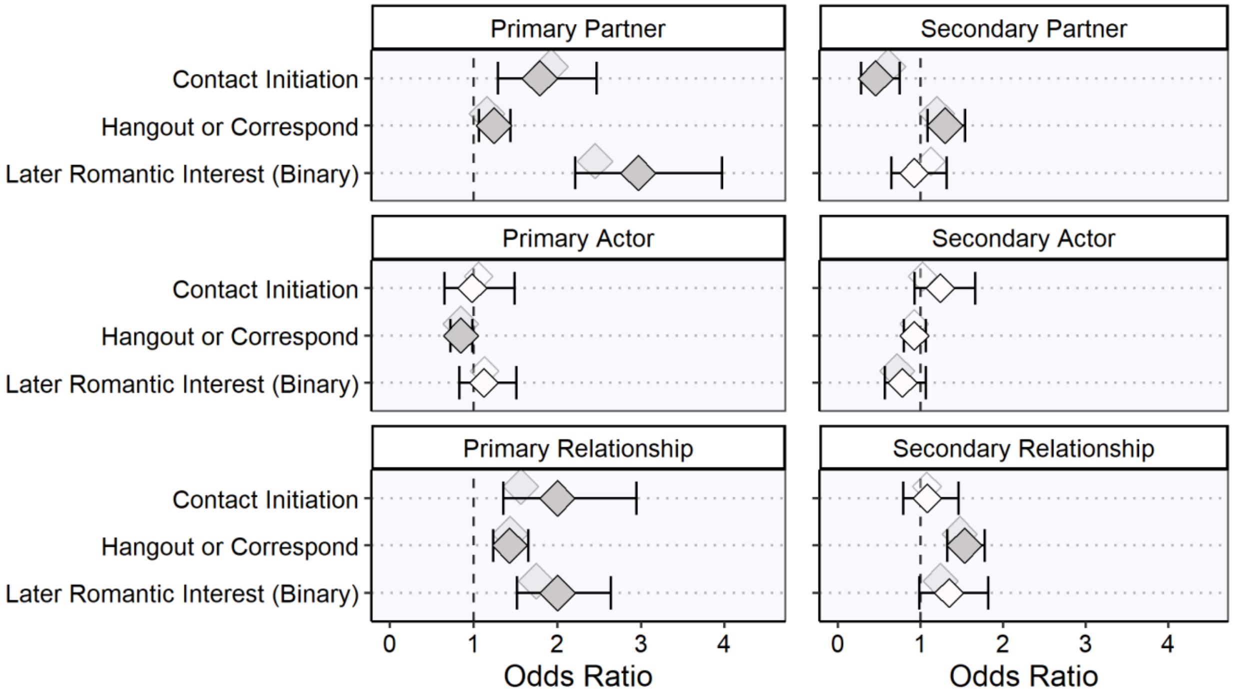

See below for a combined figure caption for Supplementary Figures S2A-S2C.

Supplementary Figure S2B

Results of Sensitivity Analyses with Random Slopes Included for Continuous Regression Models

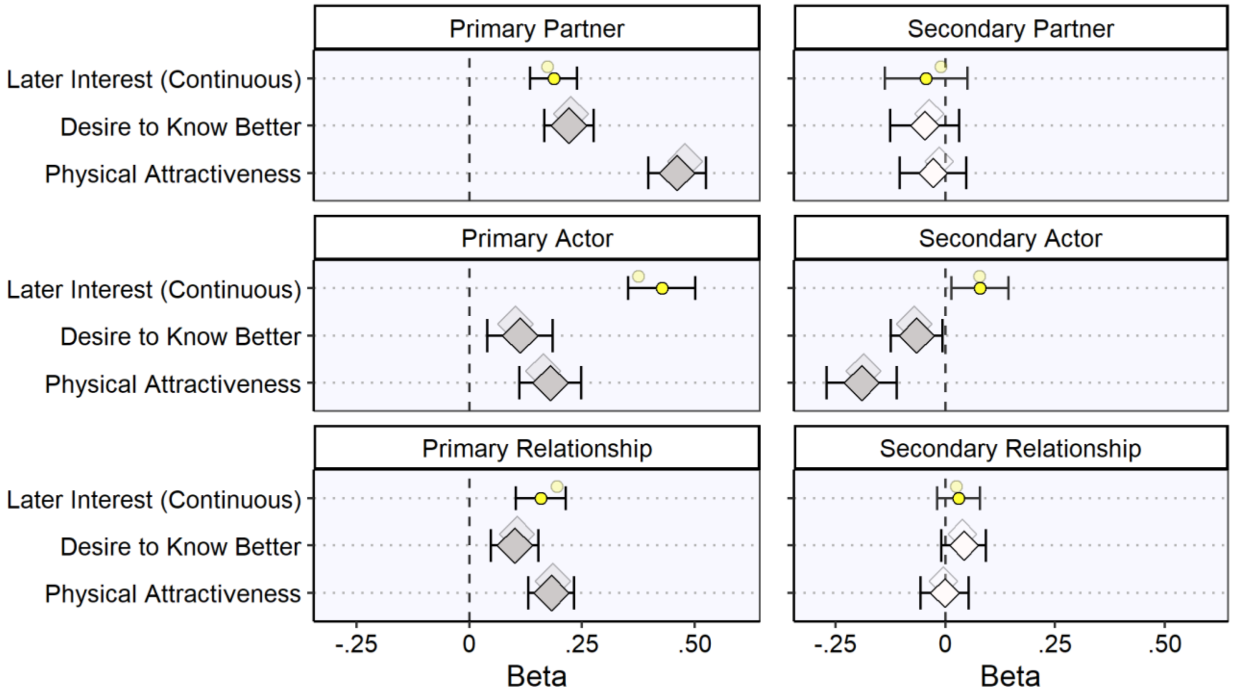

See below for a combined figure caption for Supplementary Figures S2A-S2C.

Supplementary Figure S2C

Results of Sensitivity Analyses with Random Slopes Included for Perceived Attraction from Match

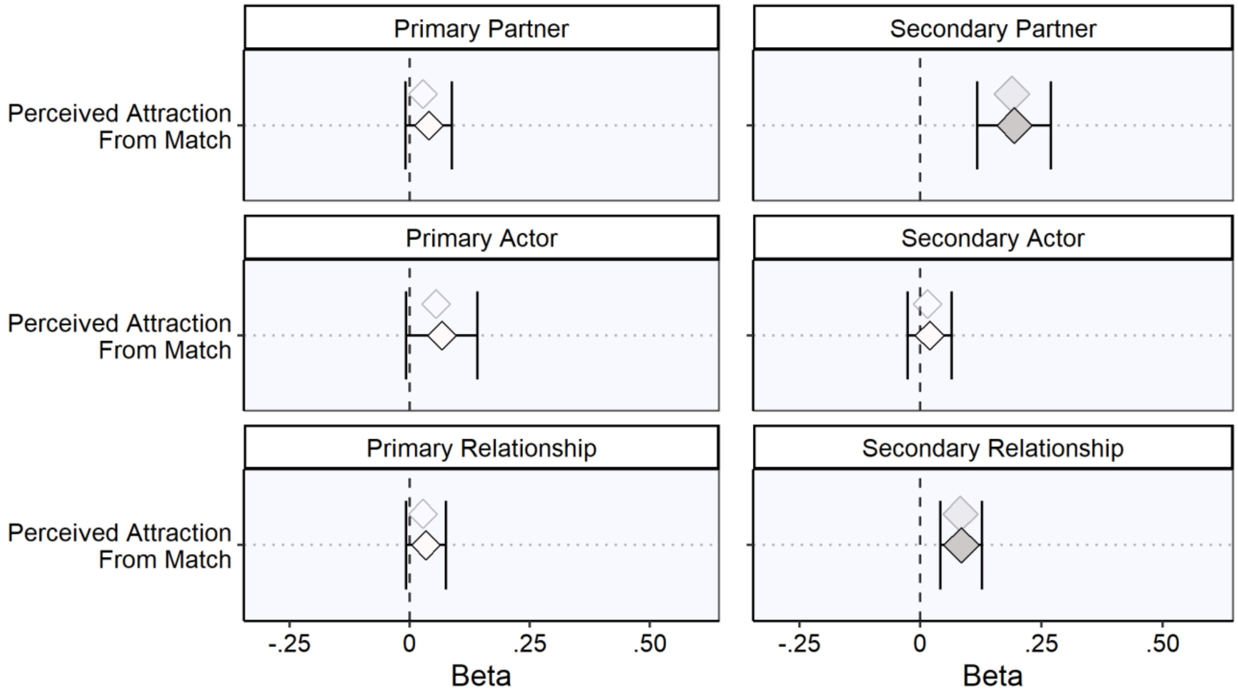

Supplementary Figures S2A-S2C. The figures show the average effect sizes and 95% confidence intervals (horizontal error bars) for the meta-analyzed associations between each primary and secondary component of initial desire (the predictor in each analysis) and each dichotomous outcome (Figure S2A), each continuous outcome (Figure S2B), and perceived attraction from match (Figure S2C) when random slopes for the SRM variable were included in the model. Individual effect sizes for each study are not shown (see Supplementary Table S9A-S9F and S10A-S10F). The dark grey diamonds indicate meta-analyzed odds ratios that were significantly different from 1 or meta-analyzed beta-weights that were significantly different from 0 ($p < .05$), and the light grey diamonds indicate meta-analyzed effect sizes that were not significant ($p \geq .05$). To demonstrate how the meta-analyzed effect size for each model changed (or did not change) in the sensitivity analyses, the effect sizes for the original analyses (i.e., the analyses that did not include random slopes for the SRM variable in each model) are indicated by the faint offset diamonds.

Supplementary Figure S3A

Results of Maximal Model Sensitivity Analyses for Logistic Regression Models

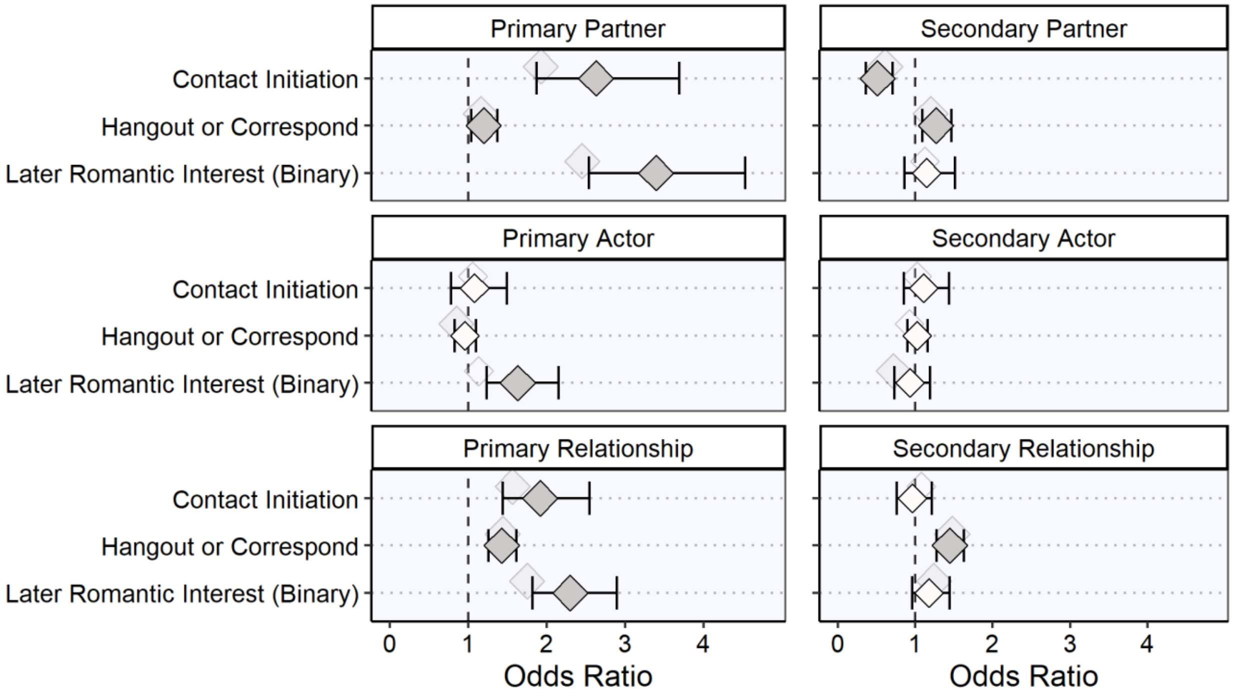

See below for a combined figure caption for Supplementary Figures S3A-S3C.

Supplementary Figure S3B

Results of Maximal Model Sensitivity Analyses for Continuous Regression Models

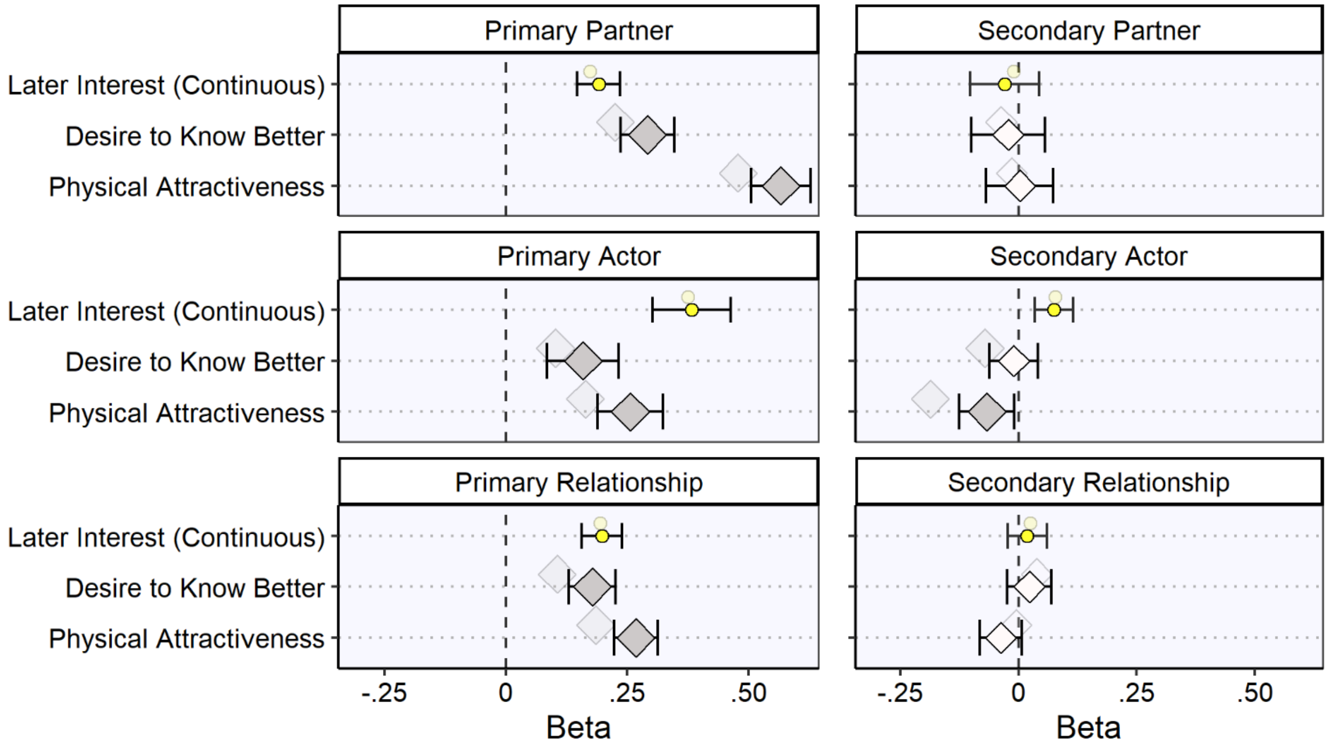

See below for a combined figure caption for Supplementary Figures S3A-S3C.

Supplementary Figure S3C

Results of Maximal Model Sensitivity Analyses for Perceived Attraction from Match

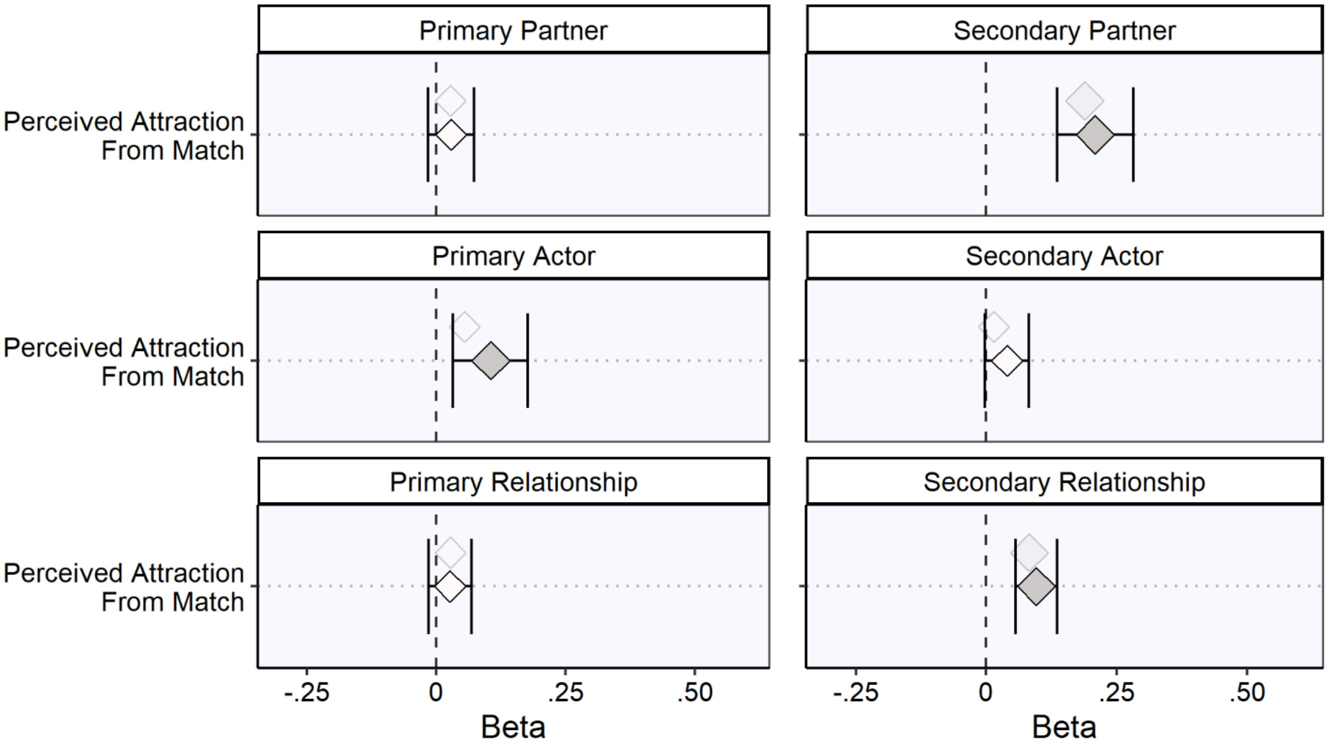

Supplementary Figures S3A-S3C. The figures show the average effect sizes and 95% confidence intervals (horizontal error bars) for the meta-analyzed associations between each primary and secondary component of initial desire (the predictor in each analysis) and each dichotomous outcome (Figure S3A), each continuous outcome (Figure S 3B), and perceived attraction from match (Figure S 3C) when each model was repeated as a maximal model with all six Primary and Secondary SRM Variables included as simultaneous predictors of each outcome in each study. Individual effect sizes for each study are not shown (see Supplementary Table S11A-S11C and S12A-S12D). The dark grey diamonds indicate meta-analyzed odds ratios that were significantly different from 1 or meta-analyzed beta-weights that were significantly different from 0 ($p < .05$), and the light grey diamonds indicate meta-analyzed effect sizes that were not significant ($p \geq .05$). To demonstrate how the meta-analyzed effect size for each model changed (or did not change) in the sensitivity analyses, the effect sizes for the original analyses (i.e., the analyses that only included one SRM variable per analysis) are indicated by the faint offset diamonds.

Supplementary Figure S4A

Results of Sensitivity Analyses for Romantic vs. Sexual Desire for Logistic Regression Models

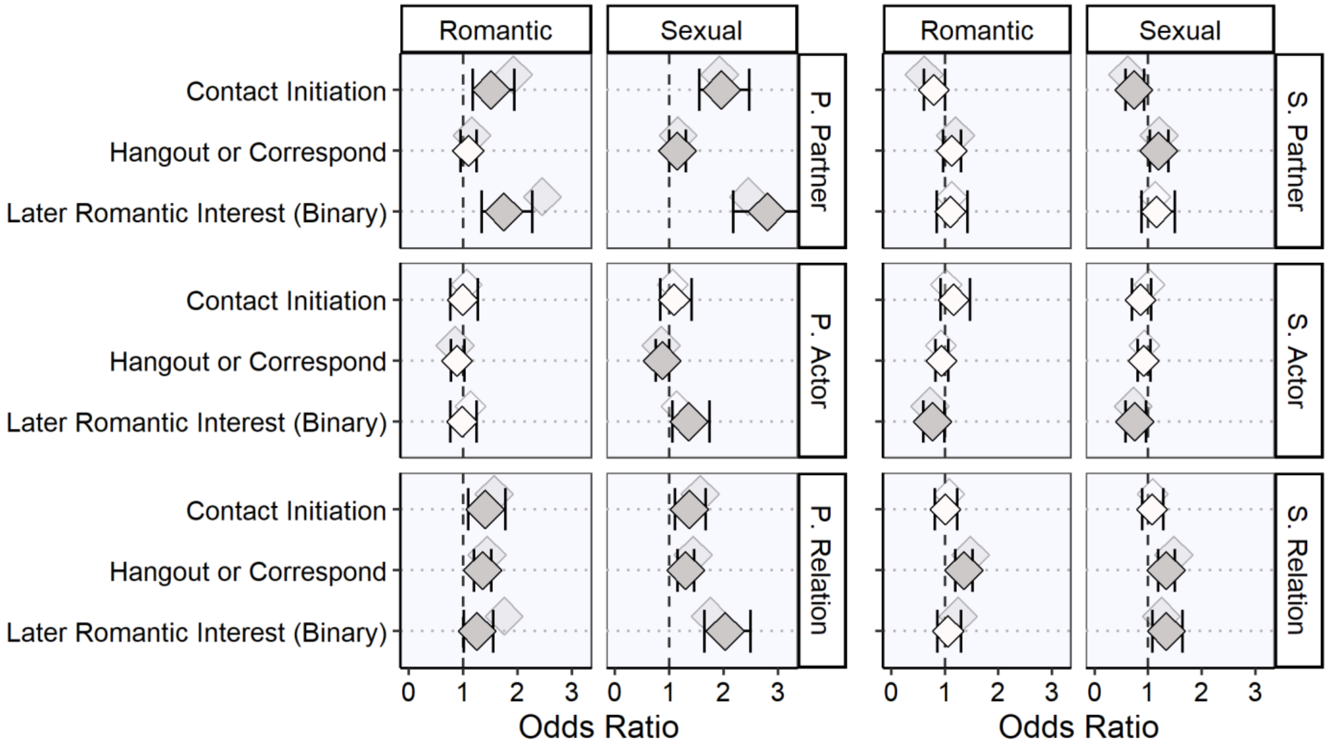

See below for a combined figure caption for Supplementary Figures S4A-S4C.

Supplementary Figure S4B

Results of Sensitivity Analyses for Romantic vs. Sexual Desire Continuous Regression Models

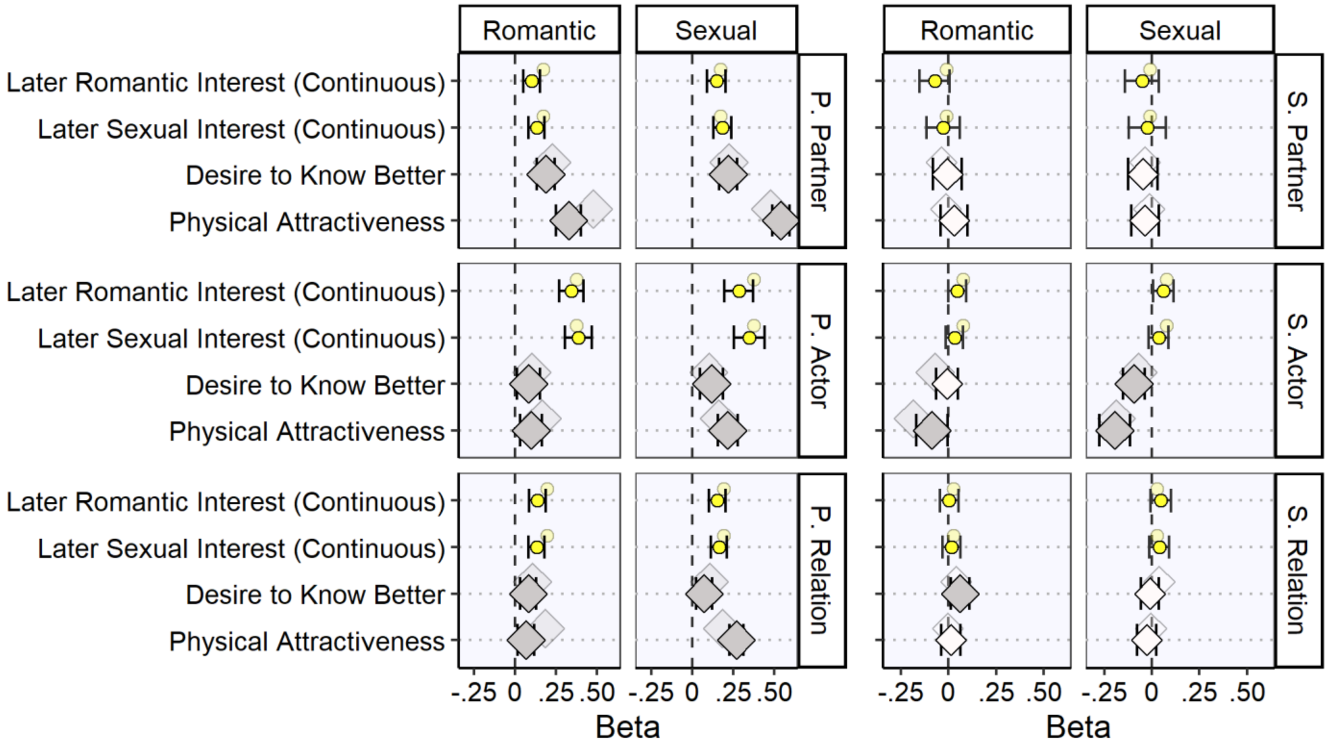

See below for a combined figure caption for Supplementary Figures S4A-S4C.

Supplementary Figure S4C

Results of Sensitivity Analyses for Romantic vs. Sexual Desire for Perceived Attraction from Match

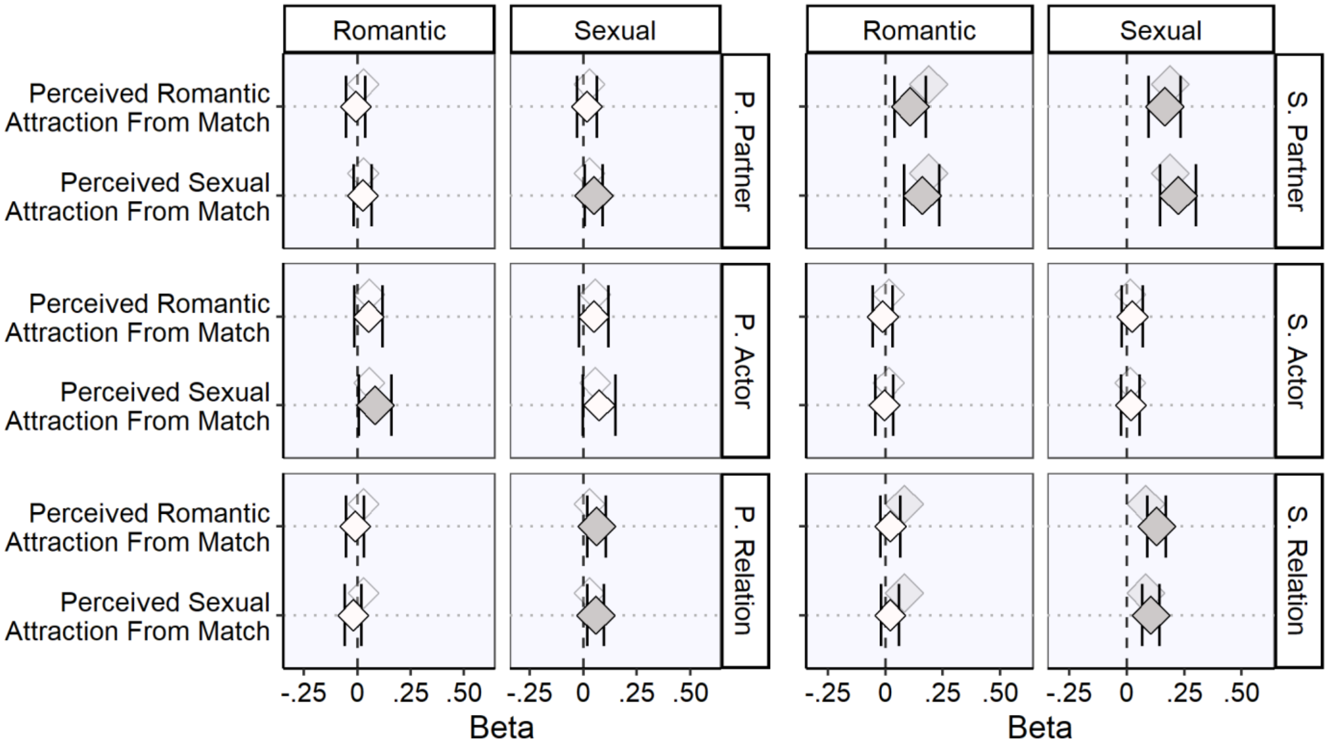

Supplementary Figures S4A-S4C. The figures show the average effect sizes and 95% confidence intervals (horizontal error bars) for the meta-analyzed associations between each primary and secondary component of initial romantic liking / initial sexual desire (the predictor in each analysis) and each dichotomous outcome (Figure S4A), each continuous outcome (Figure S4B), and perceived attraction from match (Figure S4C). These analyses also considered later romantic desire and later sexual desire separately for the continuous measure of later interest in Study 3 (see 4B) and considered later perceived romantic and sexual attraction from match separately for all three studies (see 4C). Individual effect sizes for each study are not shown (see Supplementary Table S13A-S13F and S14A– S14F). The dark grey diamonds indicate meta-analyzed odds ratios that were significantly different from 1 or meta-analyzed beta-weights that were significantly different from 0 ($p < .05$), and the light grey diamonds indicate meta-analyzed effect sizes that were not significant ($p \geq .05$). To demonstrate how the meta-analyzed effect size for each model changed (or did not change) in the sensitivity analyses, the effect sizes for the original analyses (i.e., the analyses that used SRM effects in the average of initial romantic and initial sexual desire) are indicated by the faint offset diamonds.

Abbreviations: “P.” indicates Primary, “S.” indicates “Secondary”.

Supplementary Tables

Supplementary Table S1

Sample Characteristics for Speed-Dating Studies Analyzed

Measure	Study 1<br>NSDS I	Study 2<br>NSDS II †	Study 3<br>Anime North
**Study Characteristics**			
Year of Study	2005	2007	2015
Total Speed Dating Events	7	9	8
Participants per Event	18-26	Mixed-gender: 22-24<br>All-men: 12	21-26
**Participant Characteristics**			
Total Participants in Study	163	199	210
Total Participants After Exclusion ††	163	198	198
By Gender			
Men	82	105	101
Women	81	93	97
By Race/Ethnicity			
Caucasian/White	120	136	94
Asian (South East/East Asian)	17	31	49
Latinx	4	5	5
Black	3	3	14
Arabian/West Asian	2	1	2
Indian/South Asian	9	7	11
Multi-racial	5	13	14
Other	-	-	6
Not Identified	3	2	3
Av. Age (in Years) of Participants (SD)	19.6 (1.0)	19.6 (1.2)	21.7 (3.2)
Av. Homosexual (1)-Heterosexual (7) Orientation (SD)	6.1 (1.5)	Mixed-gender: 6.5 (1.1)<br>All-men: 2.4 (2.6)	-
**Total Participants used For Sensitivity Analysis 3 †††**			
For *Romantic* Desire SRM	163	198	190
For *Sexual* Desire SRM	163	197	163
**Speed-dating Outcomes**			
Number of Matches (Total Possible)	206 (958)	252 (1,146)	264 (1,222)
Number of Participants with at Least One Match	138	172	193
Men	73	88	98
Women	65	84	95

**Follow Up Characteristics**			
Follow Up Period After Speed Dating	One Month	Four Months	Three Months
Interval	Every 3 days	Every 3 days (first five surveys)<br><br>Every 2 weeks (remaining seven surveys)	Weekly
Total Follow Up Responses	1,442	2,403	2,308
Average Number of Responses [Range]	3.5 [0-10]	4.8 [0-12]	1.9 [0-12]
**Participant Compensation**			
Attending speed-dating event	$5	$10	$0
Completing follow-up Surveys	$3 per survey<br><br>$10 bonus for 9/10 surveys	$3 per Survey<br><br>$10 bonus for 10/12 surveys	1-4 surveys: $5<br>5-7 surveys: $10<br>8-10 surveys: $15<br>11-12 surveys: $25

Supplementary Table S1. The table describes the study and sample characteristics for the three speed-dating studies analyzed. Abbreviations: Av. indicates average; SD indicates standard deviation. Cells filled with only a dash (-) indicate no data.

† In Study 2, one speed-dating event was an all-men speed-dating event. Where pertinent, we present summary data for the eight mixed-gender speed-dating events separately from the one all-men speed-dating event.

†† Participants were excluded from the SRM calculations and from further analyses if they provided incomplete responses on the measures needed to calculate initial desire (i.e., if they were missing responses to all survey items that were averaged to determine initial desire; if participants responded to at least one of the items used to determine initial desire, we averaged all available data to determine initial desire). In Study 2, there was one man excluded. In Study 3, there were five men and seven women excluded.

††† In Sensitivity Analysis 3, we assessed whether initial *romantic* liking and initial *sexual* desire (and each variable's underlying SRM components) predicted relationship initiation separately (these variables were averaged to form an overall "initial desire" variable in the original analyses; see the footnote above). Participants were excluded from the SRM calculations and from further analyses on a variable-by-variable basis if they were missing responses for either initial romantic liking or initial sexual desire. In Study 2, there was one participant who was not included in the SRM calculation for initial sexual desire (there were no cases of missing responses for initial romantic liking in Study 2). In Study 3, there were nine participants who were not included in the SRM calculations for initial romantic liking and 35 participants who were not included in the SRM calculations for initial sexual desire. See Supplementary Note S5 for more information.

Supplementary Tables S2A-S2F

These tables show the multilevel logistic regressions for each primary SRM variable and each secondary SRM variable predicting the binary outcome measures (Contact Initiation, Hangout or Correspond, Romantic Interest (Binary)). Each table gives the results for a specific SRM variable predictor. The meta-analyzed effect sizes are reported in the main text (see Table 1). See below for a combined table caption.

Supplementary Table S2A

Multilevel Logistic Regressions for Primary Partner Effects as the Independent Variable

Grouping and Study	DV	Logit	SE	OR	95% CI	*Z*	*p*	DF	*n*	Rows
**Contact Initiation**										
Study 1	Contact Initiation	0.68	0.19	1.98	[1.36, 2.86]	3.62	< .001	132	138	412
Study 2	Contact Initiation	0.64	0.19	1.90	[1.31, 2.75]	3.41	< .001	166	172	504
Study 3	Contact Initiation	0.34	0.74	1.41	[0.33, 5.99]	0.47	0.640	95	101	282
**Hangout or Correspond**										
Study 1	Hangout or Correspond	0.14	0.11	1.15	[0.93, 1.43]	1.28	0.202	124	130	1,442
Study 2	Correspond	0.06	0.11	1.06	[0.86, 1.32]	0.53	0.596	147	153	2,403
Study 2	Hangout	-0.01	0.20	0.99	[0.67, 1.47]	-0.06	0.952	147	153	2,397
Study 3	Interaction with Match	0.55	0.19	1.73	[1.19, 2.52]	2.90	0.004	108	114	2,308
**Later Romantic Interest (Binary)**										
Study 1	Later Romantic Interest	0.72	0.19	2.06	[1.42, 2.98]	3.73	< .001	124	130	1,442
Study 2	Later Romantic Interest	1.05	0.18	2.85	[2.01, 4.07]	5.81	< .001	147	153	2,403

Supplementary Table S2B

Multilevel Logistic Regressions for Primary Actor Effects as the Independent Variable

Grouping and Study	DV	Logit	SE	OR	95% CI	*Z*	*p*	DF	*n*	Rows
**Contact Initiation**										

Study 1	Contact Initiation	0.05	0.18	1.05	[0.74, 1.50]	0.27	0.788	132	138	412
Study 2	Contact Initiation	-0.18	0.24	0.83	[0.52, 1.34]	-0.77	0.442	166	172	504
Study 3	Contact Initiation	0.38	0.28	1.46	[0.84, 2.53]	1.36	0.178	95	101	282
**Hangout or Correspond**										
Study 1	Hangout or Correspond	-0.15	0.13	0.86	[0.67, 1.11]	-1.13	0.260	124	130	1,442
Study 2	Correspond	-0.25	0.11	0.78	[0.63, 0.97]	-2.29	0.024	147	153	2,403
Study 2	Hangout	-0.15	0.19	0.86	[0.59, 1.25]	-0.80	0.426	147	153	2,397
Study 3	Interaction with Match	0.05	0.21	1.05	[0.70, 1.59]	0.23	0.818	108	114	2,308
**Later Romantic Interest (Binary)**										
Study 1	Later Romantic Interest	0.14	0.20	1.16	[0.78, 1.70]	0.72	0.472	124	130	1,442
Study 2	Later Romantic Interest	0.11	0.17	1.11	[0.80, 1.56]	0.62	0.536	147	153	2,403

Supplementary Table S2C

Multilevel Logistic Regressions for Primary Relationship Effects as the Independent Variable

Grouping and Study	DV	Logit	SE	OR	95% CI	*Z*	*p*	DF	*n*	Rows
**Contact Initiation**										
Study 1	Contact Initiation	0.37	0.16	1.44	[1.06, 1.98]	2.27	0.024	132	138	412
Study 2	Contact Initiation	0.60	0.22	1.83	[1.18, 2.80]	2.73	0.008	166	172	504
Study 3	Contact Initiation	0.35	0.77	1.42	[0.31, 6.42]	0.46	0.646	95	101	282
**Hangout or Correspond**										
Study 1	Hangout or Correspond	0.24	0.10	1.27	[1.04, 1.55]	2.49	0.014	124	130	1,442
Study 2	Correspond	0.40	0.10	1.49	[1.23, 1.81]	3.95	< .001	147	153	2,403
Study 2	Hangout	0.51	0.19	1.66	[1.15, 2.42]	2.61	0.010	147	153	2,397
Study 3	Interaction with Match	0.53	0.17	1.69	[1.22, 2.37]	3.12	0.002	108	114	2,308
**Later Romantic Interest (Binary)**										
Study 1	Later Romantic Interest	0.52	0.18	1.69	[1.18, 2.39]	2.91	0.004	124	130	1,442
Study 2	Later Romantic Interest	0.58	0.14	1.78	[1.36, 2.35]	4.04	< .001	147	153	2,403

Supplementary Table S2D

Multilevel Logistic Regressions for Secondary Partner Effects as the Independent Variable

Grouping and Study	DV	Logit	SE	OR	95% CI	*Z*	*p*	DF	*n*	Rows
**Contact Initiation**										
Study 1	Contact Initiation	-0.62	0.18	0.54	[0.38, 0.77]	-3.46	< .001	132	138	412
Study 2	Contact Initiation	-0.33	0.23	0.72	[0.46, 1.13]	-1.43	0.154	166	172	504
Study 3	Contact Initiation	-0.03	0.84	0.97	[0.19, 5.03]	-0.03	0.976	134	140	282
**Hangout or Correspond**										
Study 1	Hangout or Correspond	0.20	0.14	1.22	[0.93, 1.61]	1.43	0.156	131	137	1,442
Study 2	Correspond	0.10	0.12	1.11	[0.87, 1.40]	0.85	0.396	158	164	2,403
Study 2	Hangout	-0.01	0.20	0.99	[0.67, 1.47]	-0.03	0.976	158	164	2,397
Study 3	Interaction with Match	0.52	0.19	1.69	[1.16, 2.44]	2.72	0.008	145	151	2,308
**Later Romantic Interest (Binary)**										
Study 1	Later Romantic Interest	-0.19	0.21	0.83	[0.55, 1.25]	-0.91	0.364	131	137	1,442
Study 2	Later Romantic Interest	0.33	0.18	1.39	[0.98, 1.98]	1.87	0.064	158	164	2,403

Supplementary Table S2E

Multilevel Logistic Regressions for Secondary Actor Effects as the Independent Variable

Grouping and Study	DV	Logit	SE	OR	95% CI	*Z*	*p*	DF	*n*	Rows
**Contact Initiation**										
Study 1	Contact Initiation	-0.12	0.17	0.89	[0.64, 1.24]	-0.67	0.504	132	138	412
Study 2	Contact Initiation	0.05	0.17	1.05	[0.75, 1.47]	0.28	0.780	166	172	504
Study 3	Contact Initiation	0.20	0.22	1.22	[0.79, 1.88]	0.89	0.376	134	140	282
**Hangout or Correspond**										
Study 1	Hangout or Correspond	-0.12	0.10	0.89	[0.73, 1.08]	-1.13	0.26	131	137	1,442
Study 2	Correspond	-0.21	0.11	0.81	[0.65, 1.01]	-1.97	0.050	158	164	2,403
Study 2	Hangout	0.01	0.20	1.01	[0.68, 1.49]	0.07	0.944	158	164	2,397

Study 3	Interaction with Match	0.30	0.18	1.35	[0.95, 1.92]	1.69	0.094	145	151	2,308
**Later Romantic Interest (Binary)**										
Study 1	Later Romantic Interest	-0.40	0.19	0.67	[0.46, 0.97]	-2.08	0.040	131	137	1,442
Study 2	Later Romantic Interest	-0.28	0.18	0.76	[0.53, 1.08]	-1.56	0.12	158	164	2,403

Supplementary Table S2F

Multilevel Logistic Regressions for Secondary Relationship Effects as the Independent Variable

Grouping and Study	DV	Logit	SE	OR	95% CI	*Z*	*p*	DF	*n*	Rows
**Contact Initiation**										
Study 1	Contact Initiation	0.08	0.15	1.08	[0.81, 1.45]	0.54	0.590	132	138	412
Study 2	Contact Initiation	0.06	0.16	1.06	[0.78, 1.45]	0.36	0.720	166	172	504
Study 3	Contact Initiation	0.09	0.21	1.09	[0.72, 1.65]	0.41	0.682	134	140	282
**Hangout or Correspond**										
Study 1	Hangout or Correspond	0.29	0.09	1.34	[1.12, 1.59]	3.10	0.002	131	137	1,442
Study 2	Correspond	0.49	0.10	1.64	[1.34, 1.99]	4.78	< .001	158	164	2,403
Study 2	Hangout	0.47	0.20	1.60	[1.08, 2.37]	2.37	0.018	158	164	2,397
Study 3	Interaction with Match	0.35	0.18	1.42	[1.00, 2.02]	1.99	0.048	145	151	2,308
**Later Romantic Interest (Binary)**										
Study 1	Later Romantic Interest	0.28	0.17	1.33	[0.95, 1.85]	1.64	0.104	131	137	1,442
Study 2	Later Romantic Interest	0.17	0.14	1.19	[0.90, 1.56]	1.24	0.216	158	164	2,403

Supplementary Tables S2A-S2F. The tables show a summary of each multilevel logistic regression analysis performed. The Grouping and Study column shows the which variables were grouped together when meta-analyzed (in bold), and indicate which study the coefficients come from. Study 1 indicates the NSDS I (college sample), Study 2 indicates NSDS II (college sample), and Study 3 indicates the Anime North Study (community sample). The IV in each analysis is indicated in the title of each table, and the dependent variable in each analysis is indicated in the DV column. Logit indicates the slope of the predictor, and SE indicates the standard error of prediction (in logit units). OR indicates the slope in Odds Ratio. 95% CI indicates the upper and lower 95% confidence intervals (in odds ratio units). *Z* indicates the test statistic. *n* indicates how many unique respondents were available for each analysis. DF indicates the degrees of freedom used to calculate the *p* value for each analysis and was calculated as *n* minus one

for each predictor (i.e., the one SRM independent variable) minus one for each of the four random effects (i.e., Participant ID, Partner ID, Participant ID nested within Partner ID, and Partner ID nested within Participant ID; see the Data Analysis section in the main manuscript) minus one. Rows indicates how many rows of data were used in the analysis. The *p* values listed are not corrected for multiple comparisons because the primary purpose of each analysis was to combine them using meta-analysis, and we focus our interpretation on the meta-analyzed effect sizes and confidence intervals. However, if any individual result is interpreted, the appropriate Bonferroni correction to the alpha criterion for significance is $p < .008$ (calculated as .05 * 6 analyses per dependent variable per study).

Supplementary Tables S3A-S3F

These tables show the multilevel continuous regressions for each primary SRM variable and each secondary SRM variable predicting the continuous outcome measures (Romantic Interest (Continuous), Desire to Know Better, Physical Attractiveness). Each table gives the results for a specific SRM variable predictor. The meta-analyzed effect sizes are reported in the main text (see Table 2). See below for a combined table caption.

Supplementary Table S3A

Multilevel Continuous Regression Analyses for Primary Partner Effects as the Independent Variable

Grouping and Study	DV	b	SE	95% CI	*t*	*p*	DF	*n*	Rows
**Later Interest (Continuous)**									
Study 3	Later Interest	0.17	0.05	[0.08, 0.26]	3.82	< .001	98	104	2,282
**Know Better**									
Study 1	Desire to Know Better	0.26	0.04	[0.18, 0.35]	6.13	< .001	124	130	1,439
Study 2	Desire to Know Better	0.20	0.03	[0.13, 0.27]	5.71	< .001	147	153	2,402
**Physical Attractiveness**									
Study 1	Physically Attractive Rating	0.47	0.04	[0.39, 0.56]	11.17	< .001	124	130	1,438
Study 2	Physically Attractive Rating	0.48	0.05	[0.39, 0.58]	10.34	< .001	147	153	2,400

Supplementary Table S3B

Multilevel Continuous Regression Analyses for Primary Actor Effects as the Independent Variable

Grouping and Study	DV	b	SE	95% CI	*t*	*p*	DF	*n*	Rows
**Later Interest (Continuous)**									
Study 3	Later Interest	0.37	0.08	[0.22, 0.53]	4.79	< .001	98	104	2,282
**Know Better**									
Study 1	Desire to Know Better	0.14	0.05	[0.03, 0.25]	2.58	.011	124	130	1,439
Study 2	Desire to Know Better	0.07	0.05	[-0.03, 0.17]	1.42	.158	147	153	2,402

**Physical Attractiveness**									
Study 1	Physically Attractive Rating	0.23	0.05	[0.12, 0.34]	4.15	< .001	124	130	1,438
Study 2	Physically Attractive Rating	0.12	0.04	[0.04, 0.21]	2.93	.004	147	153	2,400

Supplementary Table S3C

Multilevel Continuous Regression Analyses for Primary Relationship Effects as the Independent Variable

Grouping and Study	DV	b	SE	95% CI	*t*	*p*	DF	*n*	Rows
**Later Interest (Continuous)**									
Study 3	Later Interest	0.19	0.04	[0.11, 0.28]	4.42	< .001	98	104	2,282
**Know Better**									
Study 1	Desire to Know Better	0.11	0.04	[0.03, 0.19]	2.73	.007	124	130	1,439
Study 2	Desire to Know Better	0.10	0.03	[0.04, 0.16]	3.30	.001	147	153	2,402
**Physical Attractiveness**									
Study 1	Physically Attractive Rating	0.19	0.04	[0.12, 0.26]	5.27	< .001	124	130	1,438
Study 2	Physically Attractive Rating	0.18	0.03	[0.11, 0.24]	5.47	< .001	147	153	2,400

Supplementary Table S3D

Multilevel Continuous Regression Analyses for Secondary Partner Effects as the Independent Variable

Grouping and Study	DV	b	SE	95% CI	*t*	*p*	DF	*n*	Rows
**Later Interest (Continuous)**									
Study 3	Later Interest	-0.01	0.08	[-0.17, 0.15]	-0.13	.900	145	151	2,282
**Know Better**									
Study 1	Desire to Know Better	-0.10	0.06	[-0.22, 0.02]	-1.70	.092	131	137	1,439
Study 2	Desire to Know Better	0.01	0.05	[-0.09, 0.11]	0.23	.822	158	164	2,402
**Physical Attractiveness**									
Study 1	Physically Attractive Rating	-0.04	0.06	[-0.16, 0.08]	-0.71	.481	131	137	1,438
Study 2	Physically Attractive Rating	0.00	0.05	[-0.09, 0.09]	0.03	.972	158	164	2,400

Supplementary Table S3E

Multilevel Continuous Regression Analyses for Secondary Actor Effects as the Independent Variable

Grouping and Study	DV	b	SE	95% CI	*t*	*p*	DF	*n*	Rows
**Later Interest (Continuous)**									
Study 3	Later Interest	0.08	0.04	[-0.01, 0.16]	1.76	.080	145	151	2,282
**Know Better**									
Study 1	Desire to Know Better	-0.11	0.05	[-0.21, -0.02]	-2.34	.021	131	137	1,439
Study 2	Desire to Know Better	-0.05	0.04	[-0.12, 0.03]	-1.29	.198	158	164	2,402
**Physical Attractiveness**									
Study 1	Physically Attractive Rating	-0.19	0.06	[-0.31, -0.08]	-3.33	.001	131	137	1,438
Study 2	Physically Attractive Rating	-0.18	0.06	[-0.30, -0.07]	-3.15	.002	158	164	2,400

Supplementary Table S3F

Multilevel Continuous Regression Analyses for Secondary Relationship Effects as the Independent Variable

Grouping and Study	DV	b	SE	95% CI	*t*	*p*	DF	*n*	Rows
**Later Interest (Continuous)**									
Study 3	Later Interest	0.03	0.05	[-0.06, 0.12]	0.56	.578	145	151	2,282
**Know Better**									
Study 1	Desire to Know Better	0.07	0.04	[-0.02, 0.15]	1.60	.112	131	137	1,439
Study 2	Desire to Know Better	0.02	0.03	[-0.04, 0.09]	0.71	.481	158	164	2,402
**Physical Attractiveness**									
Study 1	Physically Attractive Rating	-0.02	0.04	[-0.10, 0.06]	-0.54	.590	131	137	1,438
Study 2	Physically Attractive Rating	0.01	0.04	[-0.06, 0.08]	0.20	.839	158	164	2,400

Supplementary Tables S3A-S3F. The tables show a summary of each multilevel continuous regression analysis performed. The Grouping and Study column shows the which variables were grouped together when meta-analyzed (in bold), and indicate which

study the coefficients come from. Study 1 indicates the NSDS I (college sample), Study 2 indicates NSDS II (college sample), and Study 3 indicates the Anime North Study (community sample). The IV in each analysis is indicated in the title of each table, and the dependent variable in each analysis is indicated in the DV column. β indicates the slope of the predictor (the standardized beta-weight), and SE indicates the standard error of prediction. 95% CI indicates the upper and lower 95% confidence intervals (on the beta scale). *t* indicates the test statistic. *n* indicates how many unique respondents were available for each analysis. DF indicates the degrees of freedom used to calculate the *p* value for each analysis and was calculated as *n* minus one for each predictor (i.e., the one SRM independent variable) and each random effect (i.e., Participant ID, Partner ID, Participant ID nested within Partner ID, and Partner ID nested within Participant ID; see the Data Analysis section in the main manuscript). Rows indicates how many rows of data were used in the analysis. The *p* values listed are not corrected for multiple comparisons because the primary purpose of each analysis was to combine them using meta-analysis, and we focus our interpretation on the meta-analyzed effect sizes and confidence intervals. However, if any individual result is interpreted, the appropriate Bonferroni correction to the alpha criterion for significance is $p < .008$ (calculated as .05 * 6 analyses per dependent variable per study).

Supplementary Tables S4A-S4F

These tables show the multilevel continuous regressions for perceived attraction from match with separate tables for each of the six SRM independent variables, with separate tables for each SRM variable predictor. The meta-analyzed effect sizes are reported in the main text (see Table 3). See below for a combined table caption.

Supplementary Table S4A

Multilevel Continuous Regression Analyses for Perceived Attraction from Match with Primary Partner Effects as the Independent Variable

Grouping and Study	DV	b	SE	95% CI	*t*	*p*	DF	*n*	rows
**Perceived Attraction from Match**									
Study 1	Perceived Attraction from Match	0.01	0.04	[-0.06, 0.09]	0.30	.767	124	130	1,439
Study 2	Perceived Attraction from Match	0.02	0.03	[-0.05, 0.08]	0.54	.593	147	153	2,402
Study 3	Perceived Attraction from Match	0.06	0.04	[-0.02, 0.15]	1.47	.145	98	104	2,281

Supplementary Table S4B

Multilevel Continuous Regression Analyses for Perceived Attraction from Match with Primary Actor Effects as the Independent Variable

Grouping and Study	DV	b	SE	95% CI	*t*	*p*	DF	*n*	rows
**Perceived Attraction from Match**									
Study 1	Perceived Attraction from Match	-0.05	0.07	[-0.18, 0.08]	-0.74	.463	124	130	1,439
Study 2	Perceived Attraction from Match	-0.01	0.06	[-0.12, 0.11]	-0.12	.903	147	153	2,402
Study 3	Perceived Attraction from Match	0.28	0.07	[0.14, 0.43]	3.83	< .001	98	104	2,281

Supplementary Table S4C

Multilevel Continuous Regression Analyses for Perceived Attraction from Match with Primary Relationship Effects as the Independent Variable

Grouping and Study	DV	b	SE	95% CI	*t*	*p*	DF	*n*	rows
**Perceived Attraction from Match**									
Study 1	Perceived Attraction from Match	0.02	0.03	[-0.05, 0.09]	0.54	.589	124	130	1,439
Study 2	Perceived Attraction from Match	0.00	0.03	[-0.06, 0.06]	0.05	.961	147	153	2,402
Study 3	Perceived Attraction from Match	0.09	0.04	[0.00, 0.17]	2.06	.042	98	104	2,281

Supplementary Table S4D

Multilevel Continuous Regression Analyses for Perceived Attraction from Match with Secondary Partner Effects as the Independent Variable

Grouping and Study	DV	b	SE	95% CI	*t*	*p*	DF	*n*	rows
**Perceived Attraction from Match**									
Study 1	Perceived Attraction from Match	0.19	0.07	[0.05, 0.32]	2.78	.006	131	137	1,439
Study 2	Perceived Attraction from Match	0.26	0.06	[0.15, 0.37]	4.68	< .001	158	164	2,402
Study 3	Perceived Attraction from Match	0.07	0.07	[-0.07, 0.21]	0.98	.331	145	151	2,281

Supplementary Table S4E

Multilevel Continuous Regression Analyses for Perceived Attraction from Match with Secondary Actor Effects as the Independent Variable

Grouping and Study	DV	b	SE	95% CI	*t*	*p*	DF	*n*	rows
**Perceived Attraction from Match**									
Study 1	Perceived Attraction from Match	-0.01	0.04	[-0.08, 0.06]	-0.29	.772	131	137	1,439
Study 2	Perceived Attraction from Match	-0.01	0.03	[-0.07, 0.06]	-0.20	.838	158	164	2,402

Study 3	Perceived Attraction from Match	0.08	0.04	[0.00, 0.16]	1.86	.064	145	151	2,281

Supplementary Table S4F

Multilevel Continuous Regression Analyses for Perceived Attraction from Match with Secondary Relationship Effects as the Independent Variable

Grouping and Study	DV	b	SE	95% CI	*t*	*p*	DF	*n*	rows
**Perceived Attraction from Match**									
Study 1	Perceived Attraction from Match	0.12	0.03	[0.06, 0.19]	3.67	< .001	131	137	1,439
Study 2	Perceived Attraction from Match	0.06	0.03	[-0.01, 0.12]	1.76	.080	158	164	2,402
Study 3	Perceived Attraction from Match	0.06	0.04	[-0.02, 0.15]	1.48	.142	145	151	2,281

Supplementary Tables S4A-S4F. The tables show a summary of each multilevel continuous regression analysis performed for Perceived Attraction from Match as the dependent variable. The Grouping and Study column shows the which variables were grouped together when meta-analyzed (in bold), and indicate which study the coefficients come from. Study 1 indicates the NSDS I (college sample), Study 2 indicates NSDS II (college sample), and Study 3 indicates the Anime North Study (community sample). The IV in each analysis is indicated in the title of each table, and the dependent variable in each analysis is indicated in the DV column. β indicates the slope of the predictor (the standardized beta-weight), and SE indicates the standard error of prediction. 95% CI indicates the upper and lower 95% confidence intervals (on the beta scale). *n* indicates how many unique respondents were available for each analysis. DF indicates the degrees of freedom used to calculate the *p* value for each analysis and was calculated as *n* minus one for each predictor (i.e., the one SRM independent variable) and each random effect (i.e., Participant ID, Partner ID, Participant ID nested within Partner ID, and Partner ID nested within Participant ID). Rows indicates how many rows of data were used in the analysis. The *p* values listed are not corrected for multiple comparisons because the primary purpose of each analysis was to combine them using meta-analysis, and we focus our interpretation on the meta-analyzed effect sizes and confidence intervals. However, if any individual result is interpreted, the appropriate Bonferroni correction to the alpha criterion for significance is $p < .008$ (calculated as .05 * 6 analyses per dependent variable per study).

Supplementary Tables S5A-S5F

These tables show the multilevel logistic regressions testing each association between outcome variables and SRM independent variables for moderation by gender (men = 0, women = 1). The tables also report the meta-analyzed coefficients for each grouping of variables. See below for a combined table caption.

Supplementary Table S5A

Multilevel Logistic Regressions and Meta-Analyses for Primary Partner Effects with Moderation by Gender

Grouping and Study	Model	Logit	SE	OR	95% CI	*Z*	*p*	DF	*n*	Rows
**Contact Initiation**										
Study 1	DV: Contact Initiation									
	Primary Partner	0.55	0.17	1.73	[1.25, 2.40]	3.27	0.002	130	138	412
	Gender (Women = 1)	-1.08	0.20	0.34	[0.23, 0.51]	-5.28	< .001	130	138	412
	Int. with Gender	-0.28	0.16	0.76	[0.56, 1.03]	-1.80	0.074	130	138	412
Study 2	DV: Contact Initiation†									
	Primary Partner	0.69	0.18	1.99	[1.39, 2.85]	3.74	< .001	166	172	504
	Gender (Women = 1)	-0.74	0.25	0.47	[0.29, 0.78]	-2.95	0.004	166	172	504
	Int. with Gender	-0.34	0.17	0.72	[0.52, 0.99]	-2.01	0.046	166	172	504
Study 3	DV: Contact Initiation									
	Primary Partner	0.87	0.33	2.40	[1.25, 4.59]	2.64	0.010	93	101	282
	Gender (Women = 1)	-1.45	0.33	0.23	[0.12, 0.45]	-4.41	< .001	93	101	282
	Int. with Gender	0.59	0.32	1.81	[0.96, 3.40]	1.85	0.068	93	101	282
Meta	DV: Contact Initiation									
	Primary Partner	0.64	0.12	1.90	[1.52, 2.39]	5.54	< .001	-	-	-
	Gender (Women = 1)	-1.04	0.14	0.35	[0.27, 0.47]	7.29	< .001	-	-	-
	Int. with Gender	-0.21	0.11	0.81	[0.66, 1.00]	1.92	0.055	-	-	-
**Hangout or Correspond**										
Study 1	DV: Hangout or Correspond									
	Primary Partner	0.12	0.10	1.12	[0.92, 1.37]	1.14	0.256	122	130	1,442

	Gender (Women = 1)	0.07	0.14	1.08	[0.81, 1.43]	0.51	0.610	122	130	1,442
	Int. with Gender	-0.30	0.10	0.74	[0.60, 0.90]	-2.97	0.004	122	130	1,442
Study 2	DV: Correspond									
	Primary Partner	0.12	0.11	1.13	[0.90, 1.41]	1.05	0.296	145	153	2,403
	Gender (Women = 1)	-0.07	0.12	0.94	[0.73, 1.19]	-0.54	0.590	145	153	2,403
	Int. with Gender	-0.29	0.12	0.75	[0.60, 0.94]	-2.45	0.016	145	153	2,403
Study 2	DV: Hangout									
	Primary Partner	0.08	0.20	1.08	[0.73, 1.60]	0.38	0.704	145	153	2,397
	Gender (Women = 1)	-0.03	0.19	0.97	[0.67, 1.40]	-0.18	0.858	145	153	2,397
	Int. with Gender	-0.45	0.20	0.64	[0.43, 0.95]	-2.21	0.028	145	153	2,397
Study 3	DV: Interaction with Match									
	Primary Partner	0.71	0.21	2.03	[1.35, 3.05]	3.42	< .001	106	114	2,308
	Gender (Women = 1)	-0.29	0.21	0.75	[0.50, 1.13]	-1.39	0.168	106	114	2,308
	Int. with Gender	0.34	0.19	1.40	[0.97, 2.03]	1.81	0.074	106	114	2,308
Meta	DV: Hangout or Correspond									
	Primary Partner	0.18	0.07	1.19	[1.04, 1.36]	2.61	0.009	-	-	-
	Gender (Women = 1)	-0.05	0.08	0.95	[0.81, 1.11]	0.66	0.508	-	-	-
	Int. with Gender	-0.23	0.07	0.79	[0.70, 0.91]	3.44	< .001	-	-	-
**Later Romantic Interest (Binary)**										
Study 1	DV: Later Romantic Interest									
	Primary Partner	0.68	0.19	1.98	[1.37, 2.86]	3.62	< .001	122	130	1,442
	Gender (Women = 1)	-0.54	0.20	0.58	[0.39, 0.87]	-2.65	0.010	122	130	1,442
	Int. with Gender	-0.28	0.18	0.76	[0.53, 1.09]	-1.50	0.136	122	130	1,442
Study 2	DV: Later Romantic Interest									
	Primary Partner	1.07	0.17	2.91	[2.07, 4.07]	6.20	< .001	145	153	2,403
	Gender (Women = 1)	-0.54	0.18	0.59	[0.41, 0.84]	-2.90	0.004	145	153	2,403
	Int. with Gender	-0.24	0.17	0.78	[0.57, 1.08]	-1.47	0.144	145	153	2,403
Meta	DV: Later Romantic Interest									
	Primary Partner	0.89	0.13	2.44	[1.90, 3.13]	7.02	< .001	-	-	-
	Gender (Women = 1)	-0.54	0.14	0.58	[0.45, 0.76]	3.93	< .001	-	-	-
	Int. with Gender	-0.26	0.12	0.77	[0.61, 0.98]	2.10	0.036	-	-	-

Supplementary Table S5B

Multilevel Logistic Regressions and Meta-Analyses for Primary Actor Effects with Moderation by Gender

Grouping and Study	Model	Logit	SE	OR	95% CI	*Z*	*p*	DF	*n*	Rows
**Contact Initiation**										
Study 1	DV: Contact Initiation									
	Primary Actor	0.11	0.16	1.11	[0.81, 1.52]	0.67	0.504	130	138	412
	Gender (Women = 1)	-1.04	0.19	0.35	[0.24, 0.51]	-5.44	< .001	130	138	412
	Int. with Gender	0.27	0.16	1.31	[0.96, 1.78]	1.67	0.098	130	138	412
Study 2	DV: Contact Initiation††									
	Primary Actor	-0.16	0.21	0.85	[0.56, 1.29]	-0.75	0.454	167	172	504
	Gender (Women = 1)	-0.67	0.22	0.51	[0.33, 0.79]	-3.06	0.002	167	172	504
	Int. with Gender	0.34	0.21	1.41	[0.92, 2.14]	1.58	0.116	167	172	504
Study 3	DV: Contact Initiation									
	Primary Actor	0.45	0.23	1.57	[1.00, 2.46]	1.98	0.050	93	101	282
	Gender (Women = 1)	-1.28	0.28	0.28	[0.16, 0.49]	-4.51	< .001	93	101	282
	Int. with Gender	0.22	0.23	1.24	[0.79, 1.94]	0.94	0.350	93	101	282
Meta	DV: Contact Initiation									
	Primary Actor	0.12	0.11	1.12	[0.90, 1.40]	1.04	0.297	-	-	-
	Gender (Women = 1)	-0.96	0.13	0.38	[0.30, 0.49]	7.49	< .001	-	-	-
	Int. with Gender	0.27	0.11	1.32	[1.06, 1.64]	2.46	0.014	-	-	-
**Hangout or Correspond**										
Study 1	DV: Hangout or Correspond									
	Primary Actor	-0.17	0.13	0.84	[0.65, 1.09]	-1.28	0.202	122	130	1,442
	Gender (Women = 1)	0.12	0.15	1.13	[0.84, 1.51]	0.81	0.420	122	130	1,442
	Int. with Gender	0.13	0.13	1.14	[0.87, 1.48]	0.97	0.334	122	130	1,442
Study 2	DV: Correspond									
	Primary Actor	-0.24	0.11	0.78	[0.63, 0.97]	-2.22	0.028	145	153	2,403
	Gender (Women = 1)	-0.05	0.12	0.95	[0.75, 1.21]	-0.39	0.698	145	153	2,403
	Int. with Gender	0.11	0.11	1.12	[0.90, 1.39]	1.00	0.318	145	153	2,403
Study 2	DV: Hangout									

	Primary Actor	-0.14	0.19	0.87	[0.60, 1.26]	-0.73	0.466	145	153	2,397
	Gender (Women = 1)	-0.05	0.19	0.96	[0.65, 1.40]	-0.24	0.810	145	153	2,397
	Int. with Gender	0.08	0.19	1.08	[0.75, 1.56]	0.42	0.676	145	153	2,397
Study 3	DV: Interaction with Match									
	Primary Actor	0.05	0.21	1.06	[0.70, 1.60]	0.26	0.796	106	114	2,308
	Gender (Women = 1)	-0.26	0.22	0.77	[0.50, 1.18]	-1.20	0.232	106	114	2,308
	Int. with Gender	0.11	0.21	1.12	[0.74, 1.69]	0.53	0.598	106	114	2,308
Meta	DV: Contact Initiation									
	Primary Actor	-0.17	0.07	0.84	[0.73, 0.97]	2.36	0.018	-	-	-
	Gender (Women = 1)	-0.03	0.08	0.97	[0.83, 1.14]	0.36	0.722	-	-	-
	Int. with Gender	0.11	0.07	1.12	[0.97, 1.29]	1.53	0.126	-	-	-
**Later Romantic Interest (Binary)**										
Study 1	DV: Later Romantic Interest									
	Primary Partner	0.13	0.2	1.14	[0.78, 1.69]	0.68	0.498	122	130	1,442
	Gender (Women = 1)	-0.53	0.22	0.59	[0.38, 0.91]	-2.41	0.018	122	130	1,442
	Int. with Gender	0.06	0.20	1.07	[0.72, 1.57]	0.32	0.750	122	130	1,442
Study 2	DV: Later Romantic Interest									
	Primary Partner	0.10	0.17	1.1	[0.79, 1.53]	0.57	0.570	145	153	2,403
	Gender (Women = 1)	-0.50	0.20	0.61	[0.41, 0.91]	-2.45	0.016	145	153	2,403
	Int. with Gender	0.08	0.17	1.08	[0.78, 1.50]	0.47	0.640	145	153	2,403
Meta	DV: Later Romantic Interest									
	Primary Partner	0.11	0.13	1.12	[0.87, 1.44]	0.87	0.382	-	-	-
	Gender (Women = 1)	-0.51	0.15	0.60	[0.45, 0.80]	3.43	< .001	-	-	-
	Int. with Gender	0.07	0.13	1.07	[0.84, 1.38]	0.56	0.572	-	-	-

Supplementary Table S5C

Multilevel Logistic Regressions and Meta-Analyses for Primary Relationship Effects with Moderation by Gender

Grouping and Study	Model	Logit	SE	OR	95% CI	*Z*	*p*	DF	*n*	Rows
**Contact Initiation**										

Study 1	DV: Contact Initiation										
		Primary Relationship	0.36	0.15	1.43	[1.07, 1.91]	2.41	0.018	130	138	412
		Gender (Women = 1)	-1.06	0.19	0.35	[0.24, 0.51]	-5.43	< .001	130	138	412
		Int. with Gender	0.08	0.15	1.08	[0.82, 1.44]	0.56	0.576	130	138	412
Study 2	DV: Contact Initiation										
		Primary Relationship	0.59	0.23	1.8	[1.15, 2.83]	2.56	0.012	164	172	504
		Gender (Women = 1)	-0.83	0.33	0.44	[0.23, 0.83]	-2.54	0.012	164	172	504
		Int. with Gender	-0.32	0.2	0.73	[0.49, 1.08]	-1.59	0.114	164	172	504
Study 3	DV: Contact Initiation										
		Primary Relationship	0.45	0.23	1.57	[1.01, 2.45]	2.00	0.048	93	101	282
		Gender (Women = 1)	-1.38	0.32	0.25	[0.13, 0.47]	-4.35	< .001	93	101	282
		Int. with Gender	0.23	0.22	1.26	[0.82, 1.92]	1.06	0.292	93	101	282
Meta	DV: Contact Initiation										
		Primary Relationship	0.43	0.11	1.54	[1.24, 1.91]	3.96	< .001	-	-	-
		Gender (Women = 1)	-1.08	0.15	0.34	[0.25, 0.45]	7.30	< .001	-	-	-
		Int. with Gender	0.01	0.10	1.01	[0.82, 1.24]	0.09	0.929	-	-	-
**Hangout or Correspond**											
Study 1	DV: Hangout or Correspond										
		Primary Relationship	0.23	0.10	1.26	[1.05, 1.53]	2.42	0.016	122	130	1,442
		Gender (Women = 1)	0.08	0.15	1.08	[0.81, 1.45]	0.52	0.604	122	130	1,442
		Int. with Gender	0.00	0.10	1.00	[0.82, 1.21]	-0.04	0.968	122	130	1,442
Study 2	DV: Correspond										
		Primary Relationship	0.44	0.10	1.55	[1.26, 1.89]	4.24	< .001	145	153	2,403
		Gender (Women = 1)	0.03	0.13	1.04	[0.81, 1.32]	0.28	0.780	145	153	2,403
		Int. with Gender	-0.23	0.11	0.79	[0.64, 0.97]	-2.22	0.028	145	153	2,403
Study 2	DV: Hangout										
		Primary Relationship	0.52	0.20	1.68	[1.14, 2.46]	2.65	0.008	145	153	2,397
		Gender (Women = 1)	0.03	0.20	1.03	[0.70, 1.51]	0.14	0.888	145	153	2,397
		Int. with Gender	-0.09	0.19	0.91	[0.62, 1.33]	-0.49	0.624	145	153	2,397
Study 3	DV: Interaction with Match										
		Primary Relationship	0.55	0.17	1.74	[1.25, 2.41]	3.28	0.002	106	114	2,308
		Gender	-0.34	0.22	0.71	[0.47, 1.09]	-1.57	0.120	106	114	2,308

		Int. with Gender	0.27	0.17	1.31	[0.95, 1.81]	1.65	0.102	106	114	2,308
Meta	DV: Hangout or Correspond										
		Primary Relationship	0.38	0.06	1.46	[1.29, 1.65]	6.12	< .001	-	-	-
		Gender (Women = 1)	-0.01	0.08	0.99	[0.85, 1.16]	0.07	0.945	-	-	-
		Int. with Gender	-0.05	0.06	0.95	[0.84, 1.07]	0.88	0.38	-	-	-
**Later Romantic Interest (Binary)**											
Study 1	DV: Later Romantic Interest										
		Primary Partner	0.55	0.18	1.73	[1.21, 2.47]	3.01	0.004	122	130	1,442
		Gender (Women = 1)	-0.57	0.23	0.57	[0.36, 0.89]	-2.49	0.014	122	130	1,442
		Int. with Gender	0.25	0.18	1.28	[0.91, 1.81]	1.41	0.162	122	130	1,442
Study 2	DV: Later Romantic Interest										
		Primary Partner	0.56	0.14	1.75	[1.32, 2.31]	3.88	< .001	145	153	2,403
		Gender (Women = 1)	-0.47	0.22	0.62	[0.41, 0.96]	-2.16	0.032	145	153	2,403
		Int. with Gender	-0.02	0.14	0.98	[0.75, 1.29]	-0.12	0.904	145	153	2,403
Meta	DV: Later Romantic Interest										
		Primary Partner	0.55	0.11	1.74	[1.39, 2.17]	4.91	< .001	-	-	-
		Gender (Women = 1)	-0.52	0.16	0.60	[0.44, 0.81]	3.28	0.001	-	-	-
		Int. with Gender	0.08	0.11	1.09	[0.88, 1.34]	0.78	0.435	-	-	-

Supplementary Table S5D

Multilevel Logistic Regressions and Meta-Analyses for Secondary Partner Effects with Moderation by Gender

Grouping and Study	Model		Logit	SE	OR	95% CI	*Z*	*p*	DF	*n*	Rows
**Contact Initiation**											
Study 1	DV: Contact Initiation										
		Secondary Partner	-0.62	0.15	0.54	[0.40, 0.73]	-3.99	< .001	130	138	412
		Gender (Women = 1)	-1.08	0.19	0.34	[0.24, 0.49]	-5.75	< .001	130	138	412
		Int. with Gender	-0.14	0.15	0.87	[0.65, 1.16]	-0.95	0.344	130	138	412
Study 2	DV: Contact Initiation†										
		Secondary Partner	-0.33	0.24	0.72	[0.45, 1.17]	-1.33	0.186	166	172	504

	Gender (Women = 1)	-0.79	0.27	0.45	[0.27, 0.77]	-2.95	0.004	166	172	504
	Int. with Gender	0.08	0.26	1.08	[0.66, 1.78]	0.31	0.756	166	172	504
Study 3	DV: Contact Initiation									
	Secondary Partner	0.15	0.24	1.16	[0.73, 1.84]	0.61	0.542	132	140	282
	Gender (Women = 1)	-1.23	0.28	0.29	[0.17, 0.51]	-4.36	< .001	132	140	282
	Int. with Gender	-0.18	0.24	0.84	[0.53, 1.33]	-0.76	0.448	132	140	282
Meta	DV: Contact Initiation									
	Secondary Partner	-0.38	0.11	0.69	[0.55, 0.86]	3.28	0.001	-	-	-
	Gender (Women = 1)	-1.04	0.13	0.35	[0.27, 0.46]	7.71	< .001	-	-	-
	Int. with Gender	-0.11	0.11	0.90	[0.72, 1.12]	0.95	0.342	-	-	-
**Hangout or Correspond**										
Study 1	DV: Hangout or Correspond									
	Secondary Partner	0.21	0.14	1.23	[0.94, 1.61]	1.48	0.142	129	137	1,442
	Gender (Women = 1)	0.12	0.15	1.13	[0.85, 1.52]	0.84	0.402	129	137	1,442
	Int. with Gender	0.17	0.14	1.18	[0.90, 1.55]	1.22	0.224	129	137	1,442
Study 2	DV: Correspond									
	Secondary Partner	0.23	0.13	1.26	[0.98, 1.63]	1.80	0.074	156	164	2,403
	Gender (Women = 1)	-0.05	0.13	0.95	[0.75, 1.22]	-0.38	0.704	156	164	2,403
	Int. with Gender	0.31	0.13	1.37	[1.07, 1.75]	2.47	0.014	156	164	2,403
Study 2	DV: Hangout									
	Secondary Partner	0.08	0.22	1.08	[0.71, 1.66]	0.37	0.712	156	164	2,397
	Gender (Women = 1)	-0.05	0.19	0.96	[0.65, 1.40]	-0.23	0.818	156	164	2,397
	Int. with Gender	0.20	0.21	1.22	[0.81, 1.85]	0.96	0.338	156	164	2,397
Study 3	DV: Interaction with Match									
	Secondary Partner	0.51	0.19	1.67	[1.15, 2.44]	2.68	0.008	143	151	2,308
	Gender (Women = 1)	-0.23	0.21	0.80	[0.53, 1.20]	-1.08	0.282	143	151	2,308
	Int. with Gender	-0.02	0.19	0.98	[0.67, 1.43]	-0.11	0.912	143	151	2,308
Meta	DV: Hangout or Correspond									
	Secondary Partner	0.25	0.08	1.29	[1.10, 1.50]	3.18	0.001	-	-	-
	Gender (Women = 1)	-0.02	0.08	0.98	[0.84, 1.14]	0.30	0.766	-	-	-
	Int. with Gender	0.20	0.08	1.22	[1.05, 1.42]	2.53	0.011	-	-	-
**Later Romantic Interest (Binary)**										

Study 1	DV: Later Romantic Interest									
	Primary Partner	-0.12	0.21	0.88	[0.59, 1.32]	-0.60	0.550	129	137	1,442
	Gender (Women = 1)	-0.48	0.22	0.62	[0.40, 0.95]	-2.17	0.032	129	137	1,442
	Int. with Gender	0.31	0.20	1.37	[0.92, 2.02]	1.57	0.118	129	137	1,442
Study 2	DV: Later Romantic Interest									
	Primary Partner	0.41	0.19	1.51	[1.03, 2.21]	2.13	0.034	156	164	2,403
	Gender (Women = 1)	-0.52	0.20	0.59	[0.40, 0.88]	-2.57	0.012	156	164	2,403
	Int. with Gender	0.14	0.19	1.15	[0.80, 1.66]	0.75	0.454	156	164	2,403
Meta	DV: Later Romantic Interest									
	Primary Partner	0.16	0.14	1.17	[0.89, 1.55]	1.14	0.256	-	-	-
	Gender (Women = 1)	-0.50	0.15	0.61	[0.45, 0.81]	3.37	< .001	-	-	-
	Int. with Gender	0.22	0.14	1.25	[0.95, 1.63]	1.62	0.106	-	-	-

Supplementary Table S5E

Multilevel Logistic Regressions and Meta-Analyses for Secondary Actor Effects with Moderation by Gender

Grouping and Study	Model	Logit	SE	OR	95% CI	*Z*	*p*	DF	*n*	Rows
**Contact Initiation**										
Study 1	DV: Contact Initiation									
	Secondary Actor	-0.14	0.16	0.87	[0.64, 1.18]	-0.89	0.376	130	138	412
	Gender (Women = 1)	-1.04	0.19	0.35	[0.24, 0.51]	-5.41	< .001	130	138	412
	Int. with Gender	0.02	0.16	1.02	[0.75, 1.39]	0.15	0.880	130	138	412
Study 2	DV: Contact Initiation									
	Secondary Actor	0.17	0.18	1.18	[0.83, 1.69]	0.93	0.354	164	172	504
	Gender (Women = 1)	-0.88	0.28	0.42	[0.24, 0.73]	-3.09	0.002	164	172	504
	Int. with Gender	0.46	0.2	1.59	[1.08, 2.33]	2.34	0.020	164	172	504
Study 3	DV: Contact Initiation									
	Secondary Actor	0.21	0.25	1.23	[0.76, 2.00]	0.85	0.396	132	140	282
	Gender (Women = 1)	-1.21	0.28	0.30	[0.17, 0.51]	-4.39	< .001	132	140	282
	Int. with Gender	0.14	0.24	1.14	[0.72, 1.83]	0.56	0.576	132	140	282

Meta	DV: Contact Initiation										
		Secondary Actor	0.03	0.11	1.03	[0.84, 1.27]	0.31	0.760	-	-	-
		Gender (Women = 1)	-1.05	0.14	0.35	[0.27, 0.46]	7.58	< .001	-	-	-
		Int. with Gender	0.18	0.11	1.20	[0.97, 1.48]	1.66	0.097	-	-	-
**Hangout or Correspond**											
Study 1	DV: Hangout or Correspond										
		Secondary Actor	-0.14	0.11	0.87	[0.71, 1.07]	-1.29	0.200	129	137	1,442
		Gender (Women = 1)	0.09	0.15	1.10	[0.82, 1.47]	0.63	0.530	129	137	1,442
		Int. with Gender	-0.10	0.10	0.91	[0.74, 1.11]	-0.95	0.344	129	137	1,442
Study 2	DV: Correspond										
		Secondary Actor	-0.22	0.11	0.80	[0.65, 0.99]	-2.02	0.046	156	164	2,403
		Gender (Women = 1)	-0.05	0.12	0.95	[0.74, 1.21]	-0.44	0.660	156	164	2,403
		Int. with Gender	-0.08	0.11	0.93	[0.75, 1.14]	-0.71	0.478	156	164	2,403
Study 2	DV: Hangout										
		Secondary Actor	0.01	0.20	1.02	[0.69, 1.50]	0.08	0.936	156	164	2,397
		Gender (Women = 1)	-0.05	0.19	0.95	[0.65, 1.40]	-0.25	0.802	156	164	2,397
		Int. with Gender	-0.06	0.20	0.94	[0.64, 1.39]	-0.29	0.772	156	164	2,397
Study 3	DV: Interaction with Match										
		Secondary Actor	0.30	0.18	1.35	[0.94, 1.94]	1.62	0.108	143	151	2,308
		Gender (Women = 1)	-0.23	0.22	0.79	[0.52, 1.21]	-1.08	0.282	143	151	2,308
		Int. with Gender	0.05	0.17	1.05	[0.75, 1.47]	0.28	0.780	143	151	2,308
Meta	DV: Hangout or Correspond										
		Secondary Actor	-0.09	0.07	0.91	[0.80, 1.04]	1.42	0.154	-	-	-
		Gender (Women = 1)	-0.04	0.08	0.97	[0.83, 1.13]	0.44	0.659	-	-	-
		Int. with Gender	-0.07	0.06	0.94	[0.82, 1.06]	1.01	0.311	-	-	-
**Later Romantic Interest (Binary)**											
Study 1	DV: Later Romantic Interest										
		Primary Partner	-0.48	0.19	0.62	[0.42, 0.91]	-2.45	0.016	129	137	1,442
		Gender (Women = 1)	-0.56	0.21	0.57	[0.38, 0.87]	-2.64	0.010	129	137	1,442
		Int. with Gender	-0.14	0.19	0.87	[0.61, 1.26]	-0.73	0.466	129	137	1,442
Study 2	DV: Later Romantic Interest										
		Primary Partner	-0.26	0.18	0.77	[0.55, 1.10]	-1.44	0.152	156	164	2,403

	Gender (Women = 1)	-0.50	0.20	0.61	[0.41, 0.90]	-2.48	0.014	156	164	2,403
	Int. with Gender	-0.01	0.17	0.99	[0.71, 1.39]	-0.06	0.952	156	164	2,403
Meta	DV: Later Romantic Interest									
	Primary Partner	-0.36	0.13	0.70	[0.54, 0.91]	2.72	0.007	-	-	-
	Gender (Women = 1)	-0.53	0.15	0.59	[0.44, 0.78]	3.62	< .001	-	-	-
	Int. with Gender	-0.07	0.13	0.93	[0.73, 1.20]	0.54	0.591	-	-	-

Supplementary Table S5F

Multilevel Logistic Regressions and Meta-Analyses for Secondary Relationship Effects with Moderation by Gender

Grouping and Study	Model	Logit	SE	OR	95% CI	*Z*	*p*	DF	*n*	Rows
**Contact Initiation**										
Study 1	DV: Contact Initiation									
	Secondary Relationship	0.00	0.14	1.00	[0.76, 1.33]	0.01	0.992	130	138	412
	Gender (Women = 1)	-1.02	0.19	0.36	[0.25, 0.52]	-5.40	< .001	130	138	412
	Int. with Gender	-0.22	0.14	0.80	[0.61, 1.06]	-1.55	0.124	130	138	412
Study 2	DV: Contact Initiation									
	Secondary Relationship	0.03	0.16	1.03	[0.74, 1.42]	0.16	0.874	164	172	504
	Gender (Women = 1)	-0.79	0.27	0.45	[0.27, 0.77]	-2.94	0.004	164	172	504
	Int. with Gender	-0.14	0.17	0.87	[0.62, 1.20]	-0.85	0.396	164	172	504
Study 3	DV: Contact Initiation									
	Secondary Relationship	0.00	0.23	1.00	[0.64, 1.56]	0.00	0.999	132	140	282
	Gender (Women = 1)	-1.21	0.28	0.3	[0.17, 0.51]	-4.4	< .001	132	140	282
	Int. with Gender	-0.16	0.22	0.85	[0.55, 1.32]	-0.71	0.478	132	140	282
Meta	DV: Contact Initiation									
	Secondary Relationship	0.01	0.10	1.01	[0.83, 1.22]	0.10	0.920	-	-	-
	Gender (Women = 1)	-1.01	0.14	0.36	[0.28, 0.47]	7.48	< .001	-	-	-
	Int. with Gender	-0.18	0.10	0.83	[0.69, 1.01]	1.87	0.062	-	-	-
**Hangout or Correspond**										
Study 1	DV: Hangout or Correspond									

		Secondary Relationship	0.30	0.09	1.35	[1.12, 1.62]	3.19	0.002	129	137	1,442
		Gender (Women = 1)	0.14	0.15	1.15	[0.85, 1.54]	0.91	0.364	129	137	1,442
		Int. with Gender	-0.12	0.09	0.88	[0.74, 1.06]	-1.33	0.186	129	137	1,442
Study 2	DV: Correspond										
		Secondary Relationship	0.53	0.10	1.70	[1.38, 2.08]	5.10	< .001	156	164	2,403
		Gender (Women = 1)	-0.10	0.12	0.90	[0.71, 1.15]	-0.81	0.420	156	164	2,403
		Int. with Gender	0.20	0.10	1.22	[1.00, 1.48]	1.94	0.054	156	164	2,403
Study 2	DV: Hangout										
		Secondary Relationship	0.52	0.20	1.68	[1.13, 2.50]	2.57	0.012	156	164	2,397
		Gender (Women = 1)	-0.06	0.19	0.94	[0.65, 1.37]	-0.32	0.750	156	164	2,397
		Int. with Gender	0.24	0.20	1.27	[0.86, 1.87]	1.22	0.224	156	164	2,397
Study 3	DV: Interaction with Match										
		Secondary Relationship	0.29	0.18	1.34	[0.94, 1.92]	1.61	0.110	143	151	2,308
		Gender (Women = 1)	-0.24	0.22	0.79	[0.51, 1.21]	-1.10	0.274	143	151	2,308
		Int. with Gender	-0.22	0.17	0.80	[0.57, 1.12]	-1.28	0.202	143	151	2,308
Meta	DV: Contact Initiation										
		Secondary Relationship	0.40	0.06	1.49	[1.32, 1.68]	6.48	< .001	-	-	-
		Gender (Women = 1)	-0.05	0.08	0.96	[0.82, 1.12]	0.57	0.568	-	-	-
		Int. with Gender	0.01	0.06	1.01	[0.90, 1.14]	0.23	0.819	-	-	-
**Later Romantic Interest (Binary)**											
Study 1	DV: Later Romantic Interest										
		Primary Partner	0.28	0.17	1.32	[0.94, 1.86]	1.61	0.110	129	137	1,442
		Gender (Women = 1)	-0.52	0.22	0.60	[0.39, 0.91]	-2.39	0.018	129	137	1,442
		Int. with Gender	0.00	0.17	1.00	[0.72, 1.40]	0.02	0.984	129	137	1,442
Study 2	DV: Later Romantic Interest										
		Primary Partner	0.21	0.14	1.23	[0.93, 1.63]	1.42	0.158	156	164	2,403
		Gender (Women = 1)	-0.53	0.21	0.59	[0.39, 0.88]	-2.57	0.012	156	164	2,403
		Int. with Gender	0.13	0.14	1.14	[0.87, 1.51]	0.94	0.348	156	164	2,403
Meta	DV: Later Romantic Interest										
		Primary Partner	0.24	0.11	1.27	[1.02, 1.58]	2.13	0.033	-	-	-
		Gender (Women = 1)	-0.52	0.15	0.59	[0.44, 0.79]	3.51	< .001	-	-	-
		Int. with Gender	0.08	0.11	1.08	[0.88, 1.34]	0.74	0.461	-	-	-

Supplementary Tables S5A-S5F. The tables show a summary of each multilevel logistic regression analysis performed testing for moderation by gender, as well as the meta-analyzed effect sizes (for a description of the analytic methods used see Supplementary Note 1, and for commentary on the results of these analyses see Supplementary Note 2). The Grouping and Study column shows the which variables were grouped together when meta-analyzed (in bold), and indicate which study the coefficients come from. Study 1 indicates the NSDS I (college sample), Study 2 indicates NSDS II (college sample), Study 3 indicates the Anime North Study (community sample), and Meta indicates the meta-analyzed effect sizes across the category. Model indicates the analysis variables: the variable labeled "DV" is the dependent variable, and the variables listed underneath are the independent variables. "Int. with Gender" indicates the interaction between gender and the SRM variable in the analysis. Logit indicates the slope of the predictor, and SE indicates the standard error of prediction (in logit units). OR indicates the slope in Odds Ratio. 95% CI indicates the upper and lower 95% confidence intervals (in odds ratio units). Z indicates the test statistic. n indicates how many unique respondents were available for each analysis. DF indicates the degrees of freedom used to calculate the p value for each analysis and was calculated as n minus one for each predictor (i.e., the one SRM independent variable) minus one for each random effect minus one. Most models included four random effects: Participant ID, Partner ID, Participant ID nested within Partner ID, and Partner ID nested within Participant ID. In some cases, random effects were dropped from the analysis—see the note below†. Rows indicates how many rows of data were used in the analysis. The p values listed are not corrected for multiple comparisons because the primary purpose of each analysis was to combine them using meta-analysis, and we focus our interpretation on the meta-analyzed effect sizes and confidence intervals. However, if the results from individual analyses are interpreted, the appropriate Bonferroni correction to the alpha criterion for significance is $p < .008$ (calculated as .05 * 6 analyses per dependent variable per study).

† For the analyses marked with the dagger symbol above, some random effects were removed from the model because when they were included the models produced implausibly low standard error estimates (in turn producing implausibly large Z test statistics). The models marked with one dagger (†) indicate that the nested random effects were removed (Participant ID within Partner ID, and Partner ID within Participant ID) and Participant ID and Partner ID were kept in the model, and the models marked with two daggers (††) indicate that the nested random effects and Partner ID were removed from the model and Participant ID was kept in the model. Removing random effects from these models resulted in more plausible standard error estimates. The original models with the nested random effects are available upon request.

Supplementary Tables S6A-S6F

These tables show the multilevel continuous regressions testing each association between outcome variables (including romantic desire outcomes and perceived attraction from match) and SRM independent variables for moderation by gender (0 = men, 1 = women). The tables also report the meta-analyzed coefficients for each grouping of variables. See below for a combined table caption.

Supplementary Table S6A

Continuous Regression Analyses and Meta-Analyses with Primary Partner Effects and Moderation by Gender

Grouping and Study	Model	β	SE	95% CI	*t*	*p*	DF	*n*	Rows
**Later Interest (Continuous)**									
Study 3	DV: Later Interest								
	Primary Partner	0.17	0.05	[0.08, 0.27]	3.56	< .001	96	104	2,282
	Gender (Women = 1)	-0.36	0.08	[-0.51, -0.21]	-4.82	< .001	96	104	2,282
	Int. with Gender	-0.01	0.04	[-0.10, 0.07]	-0.30	.762	96	104	2,282
**Desire to Know Better**									
Study 1	DV: Desire to Know Better								
	Primary Partner	0.26	0.04	[0.18, 0.34]	6.18	< .001	122	130	1,439
	Gender (Women = 1)	-0.18	0.06	[-0.30, -0.07]	-3.09	.002	122	130	1,439
	Int. with Gender	-0.03	0.04	[-0.11, 0.05]	-0.69	.493	122	130	1,439
Study 2	DV: Desire to Know Better								
	Primary Partner	0.21	0.04	[0.14, 0.28]	5.89	< .001	145	153	2,402
	Gender (Women = 1)	-0.11	0.05	[-0.21, -0.01]	-2.15	.034	145	153	2,402
	Int. with Gender	-0.05	0.04	[-0.12, 0.02]	-1.38	.169	145	153	2,402
Meta	DV: Know Better								
	Primary Partner	0.23	0.03	[0.18, 0.28]	8.48	< .001	-	-	-
	Gender (Women = 1)	-0.14	0.04	[-0.22, -0.07]	3.65	< .001	-	-	-
	Int. with Gender	-0.04	0.03	[-0.09, 0.01]	1.49	.135	-	-	-
**Physical Attractiveness**									
Study 1	DV: Physically Attractive Rating								

	Primary Partner	0.47	0.04	[0.39, 0.55]	11.52	< .001	122	130	1,438
	Gender (Women = 1)	-0.26	0.06	[-0.38, -0.14]	-4.34	< .001	122	130	1,438
	Int. with Gender	-0.06	0.04	[-0.13, 0.02]	-1.39	.166	122	130	1,438
Study 2	DV: Physically Attractive Rating								
	Primary Partner	0.48	0.04	[0.40, 0.57]	10.80	< .001	145	153	2,400
	Gender (Women = 1)	-0.24	0.05	[-0.34, -0.13]	-4.56	< .001	145	153	2,400
	Int. with Gender	-0.03	0.05	[-0.11, 0.06]	-0.55	.580	145	153	2,400
Meta	DV: Physical Attractiveness								
	Primary Partner	0.47	0.03	[0.42, 0.53]	15.79	< .001	-	-	-
	Gender (Women = 1)	-0.25	0.04	[-0.33, -0.17]	6.29	< .001	-	-	-
	Int. with Gender	-0.04	0.03	[-0.10, 0.02]	1.41	.158	-	-	-
**Perceived Attraction from Match**									
Study 1	DV: Perceived Attraction from Match								
	Primary Partner	0.01	0.04	[-0.06, 0.08]	0.25	.806	122	130	1,439
	Gender (Women = 1)	-0.18	0.07	[-0.31, -0.05]	-2.65	.009	122	130	1,439
	Int. with Gender	-0.03	0.04	[-0.1, 0.04]	-0.79	.431	122	130	1,439
Study 2	DV: Perceived Attraction from Match								
	Primary Partner	0.04	0.03	[-0.03, 0.1]	1.08	.284	145	153	2,402
	Gender (Women = 1)	-0.26	0.05	[-0.37, -0.16]	-4.89	< .001	145	153	2,402
	Int. with Gender	-0.05	0.03	[-0.11, 0.02]	-1.43	.156	145	153	2,402
Study 3	DV: Perceived Attraction from Match								
	Primary Partner	0.08	0.05	[-0.02, 0.17]	1.64	.105	96	104	2,281
	Gender (Women = 1)	-0.23	0.07	[-0.37, -0.08]	-3.12	.002	96	104	2,281
	Int. with Gender	0.02	0.04	[-0.07, 0.1]	0.44	.664	96	104	2,281
Meta	DV: Perceived Attraction from Match								
	Primary Partner	0.04	0.02	[-0.01, 0.08]	1.62	.105	-	-	-
	Gender (Women = 1)	-0.23	0.04	[-0.3, -0.16]	6.30	< .001	-	-	-
	Int. with Gender	-0.03	0.02	[-0.07, 0.02]	1.16	.245	-	-	-

Supplementary Table S6B

Continuous Regression Analyses and Meta-Analyses with Primary Actor Effects and Moderation by Gender

Grouping and Study	Model	β	SE	95% CI	*t*	*p*	DF	*n*	Rows
**Later Interest (Continuous)**									
Study 3	DV: Later Interest								
	Primary Partner	0.38	0.07	[0.24, 0.52]	5.43	< .001	96	104	2,282
	Gender (Women = 1)	-0.36	0.07	[-0.49, -0.22]	-5.28	< .001	96	104	2,282
	Int. with Gender	-0.15	0.07	[-0.29, -0.01]	-2.12	.037	96	104	2,282
**Desire to Know Better**									
Study 1	DV: Desire to Know Better								
	Primary Actor	0.15	0.05	[0.04, 0.25]	2.67	.009	122	130	1,439
	Gender (Women = 1)	-0.18	0.06	[-0.3, -0.05]	-2.80	.006	122	130	1,439
	Int. with Gender	0.0	0.06	[-0.11, 0.11]	-0.08	.940	122	130	1,439
Study 2	DV: Desire to Know Better								
	Primary Actor	0.06	0.05	[-0.03, 0.16]	1.28	.204	145	153	2,402
	Gender (Women = 1)	-0.12	0.05	[-0.23, -0.01]	-2.17	.032	145	153	2,402
	Int. with Gender	-0.05	0.05	[-0.15, 0.05]	-1.05	.298	145	153	2,402
Meta	DV: Know Better								
	Primary Actor	0.10	0.04	[0.03, 0.17]	2.74	.006	-	-	-
	Gender (Women = 1)	-0.14	0.04	[-0.22, -0.06]	3.47	< .001	-	-	-
	Int. with Gender	-0.03	0.04	[-0.1, 0.04]	0.83	.404	-	-	-
**Physical Attractiveness**									
Study 1	DV: Physically Attractive Rating								
	Primary Actor	0.23	0.05	[0.12, 0.34]	4.24	< .001	122	130	1,438
	Gender (Women = 1)	-0.23	0.07	[-0.38, -0.08]	-3.09	.002	122	130	1,438
	Int. with Gender	-0.01	0.06	[-0.12, 0.1]	-0.25	.804	122	130	1,438
Study 2	DV: Physically Attractive Rating								
	Primary Actor	0.12	0.04	[0.03, 0.2]	2.75	.007	145	153	2,400
	Gender	-0.24	0.06	[-0.37, -0.11]	-3.67	< .001	145	153	2,400
	Int. with Gender	-0.03	0.04	[-0.12, 0.05]	-0.79	.433	145	153	2,400
Meta	DV: Physical Attractiveness								
	Primary Actor	0.16	0.03	[0.09, 0.23]	4.77	< .001	-	-	-
	Gender (Women = 1)	-0.23	0.05	[-0.33, -0.14]	4.80	< .001	-	-	-

	Int. with Gender	-0.03	0.03	[-0.09, 0.04]	0.78	.437	-	-	-
**Perceived Attraction from Match**									
Study 1	DV: Perceived Attraction from Match								
	Primary Actor	-0.03	0.07	[-0.16, 0.1]	-0.41	.681	122	130	1,439
	Gender (Women = 1)	-0.19	0.07	[-0.32, -0.05]	-2.72	.007	122	130	1,439
	Int. with Gender	-0.11	0.07	[-0.24, 0.02]	-1.62	.107	122	130	1,439
Study 2	DV: Perceived Attraction from Match								
	Primary Actor	-0.01	0.05	[-0.11, 0.1]	-0.16	.872	145	153	2,402
	Gender (Women = 1)	-0.26	0.06	[-0.37, -0.15]	-4.70	< .001	145	153	2,402
	Int. with Gender	0.02	0.05	[-0.08, 0.13]	0.42	.678	145	153	2,402
Study 3	DV: Perceived Attraction from Match								
	Primary Actor	0.29	0.07	[0.15, 0.43]	4.07	< .001	96	104	2,281
	Gender (Women = 1)	-0.23	0.07	[-0.36, -0.09]	-3.36	.001	96	104	2,281
	Int. with Gender	-0.05	0.07	[-0.2, 0.09]	-0.76	.450	96	104	2,281
Meta	DV: Perceived Attraction from Match								
	Primary Actor	0.06	0.04	[-0.01, 0.13]	1.72	.086	-	-	-
	Gender (Women = 1)	-0.23	0.04	[-0.3, -0.16]	6.33	< .001	-	-	-
	Int. with Gender	-0.03	0.04	[-0.11, 0.04]	0.97	.331	-	-	-

Supplementary Table S6C

Continuous Regression Analyses and Meta-Analyses with Primary Relationship Effects and Moderation by Gender

Grouping and Study	Model	β	SE	95% CI	*t*	*p*	DF	*n*	Rows
**Later Interest (Continuous)**									
Study 3	DV: Later Interest								
	Primary Partner	0.21	0.04	[0.12, 0.3]	4.84	< .001	96	104	2,282
	Gender (Women = 1)	-0.38	0.08	[-0.53, -0.23]	-4.98	< .001	96	104	2,282
	Int. with Gender	0.07	0.04	[-0.01, 0.15]	1.65	.102	96	104	2,282
**Desire to Know Better**									
Study 1	DV: Desire to Know Better								

	Primary Relationship	0.11	0.04	[0.03, 0.19]	2.80	.006	122	130	1,439
	Gender (Women = 1)	-0.17	0.06	[-0.3, -0.05]	-2.78	.006	122	130	1,439
	Int. with Gender	0.0	0.04	[-0.08, 0.08]	-0.02	.986	122	130	1,439
Study 2	DV: Desire to Know Better								
	Primary Relationship	0.10	0.03	[0.04, 0.16]	3.21	.002	145	153	2,402
	Gender (Women = 1)	-0.10	0.05	[-0.21, 0.01]	-1.83	.069	145	153	2,402
	Int. with Gender	-0.01	0.03	[-0.08, 0.05]	-0.39	.697	145	153	2,402
Meta	DV: Know Better								
	Primary Relationship	0.11	0.02	[0.06, 0.15]	4.25	< .001	-	-	-
	Gender (Women = 1)	-0.13	0.04	[-0.21, -0.05]	3.21	.001	-	-	-
	Int. with Gender	-0.01	0.03	[-0.06, 0.04]	0.32	.752	-	-	-
**Physical Attractiveness**									
Study 1	DV: Physically Attractive Rating								
	Primary Relationship	0.18	0.04	[0.11, 0.26]	5.05	< .001	122	130	1,438
	Gender (Women = 1)	-0.22	0.08	[-0.37, -0.07]	-2.94	.004	122	130	1,438
	Int. with Gender	0.08	0.04	[0, 0.15]	2.05	.043	122	130	1,438
Study 2	DV: Physically Attractive Rating								
	Primary Relationship	0.17	0.03	[0.11, 0.24]	5.17	< .001	145	153	2,400
	Gender (Women = 1)	-0.23	0.07	[-0.37, -0.1]	-3.43	< .001	145	153	2,400
	Int. with Gender	0.0	0.03	[-0.06, 0.07]	0.12	.904	145	153	2,400
Meta	DV: Physical Attractiveness								
	Primary Relationship	0.18	0.02	[0.13, 0.23]	7.22	< .001	-	-	-
	Gender (Women = 1)	-0.23	0.05	[-0.33, -0.13]	4.52	< .001	-	-	-
	Int. with Gender	0.04	0.02	[-0.01, 0.09]	1.47	.142	-	-	-
**Perceived Attraction from Match**									
Study 1	DV: Perceived Attraction from Match								
	Primary Relationship	0.02	0.03	[-0.05, 0.09]	0.56	.580	122	130	1,439
	Gender (Women = 1)	-0.18	0.07	[-0.31, -0.05]	-2.64	.009	122	130	1,439
	Int. with Gender	0.01	0.04	[-0.05, 0.08]	0.42	.677	122	130	1,439
Study 2	DV: Perceived Attraction from Match								
	Primary Relationship	-0.01	0.03	[-0.07, 0.05]	-0.28	.781	145	153	2,402
	Gender (Women = 1)	-0.27	0.05	[-0.37, -0.16]	-4.92	< .001	145	153	2,402

	Int. with Gender	0.0	0.03	[-0.06, 0.07]	0.09	.927	145	153	2,402
Study 3	DV: Perceived Attraction from Match								
	Primary Relationship	0.10	0.04	[0.02, 0.19]	2.39	.019	96	104	2,281
	Gender (Women = 1)	-0.23	0.07	[-0.38, -0.09]	-3.23	.002	96	104	2,281
	Int. with Gender	0.06	0.04	[-0.03, 0.14]	1.32	.190	96	104	2,281
Meta	DV: Perceived Attraction from Match								
	Primary Relationship	0.03	0.02	[-0.01, 0.07]	1.30	.194	-	-	-
	Gender (Women = 1)	-0.23	0.04	[-0.31, -0.16]	6.37	< .001	-	-	-
	Int. with Gender	0.02	0.02	[-0.02, 0.06]	0.96	.339	-	-	-

Supplementary Table S6D

Continuous Regression Analyses and Meta-Analyses with Secondary Partner Effects and Moderation by Gender

Grouping and Study	Model	β	SE	95% CI	*t*	*p*	DF	*n*	Rows
**Later Interest (Continuous)**									
Study 3	DV: Later Interest								
	Primary Partner	-0.02	0.07	[-0.16, 0.13]	-0.23	.816	143	151	2,282
	Gender (Women = 1)	-0.35	0.08	[-0.5, -0.19]	-4.44	< .001	143	151	2,282
	Int. with Gender	0.06	0.07	[-0.08, 0.21]	0.87	.387	143	151	2,282
**Desire to Know Better**									
Study 1	DV: Desire to Know Better								
	Secondary Partner	-0.10	0.06	[-0.21, 0.02]	-1.72	.088	129	137	1,439
	Gender (Women = 1)	-0.17	0.06	[-0.3, -0.05]	-2.68	.008	129	137	1,439
	Int. with Gender	-0.01	0.06	[-0.13, 0.1]	-0.21	.834	129	137	1,439
Study 2	DV: Desire to Know Better								
	Secondary Partner	0.02	0.06	[-0.08, 0.13]	0.44	.658	156	164	2,402
	Gender (Women = 1)	-0.11	0.06	[-0.22, 0]	-1.97	.051	156	164	2,402
	Int. with Gender	0.02	0.05	[-0.09, 0.13]	0.36	.716	156	164	2,402
Meta	DV: Know Better								
	Secondary Partner	-0.03	0.04	[-0.11, 0.04]	0.87	.386	-	-	-

	Gender (Women = 1)	-0.14	0.04	[-0.22, -0.05]	3.24	.001	-	-	-
	Int. with Gender	0.0	0.04	[-0.07, 0.08]	0.12	.902	-	-	-
**Physical Attractiveness**									
Study 1	DV: Physically Attractive Rating								
	Secondary Partner	-0.04	0.06	[-0.16, 0.08]	-0.72	.474	129	137	1,438
	Gender (Women = 1)	-0.22	0.08	[-0.37, -0.07]	-2.87	.005	129	137	1,438
	Int. with Gender	0.01	0.06	[-0.11, 0.13]	0.17	.862	129	137	1,438
Study 2	DV: Physically Attractive Rating								
	Secondary Partner	0.04	0.05	[-0.06, 0.14]	0.83	.408	156	164	2,400
	Gender	-0.23	0.06	[-0.36, -0.1]	-3.51	< .001	156	164	2,400
	Int. with Gender	0.09	0.05	[-0.01, 0.18]	1.80	.074	156	164	2,400
Meta	DV: Physical Attractiveness								
	Secondary Partner	0.01	0.04	[-0.07, 0.08]	0.19	.849	-	-	-
	Gender (Women = 1)	-0.22	0.05	[-0.32, -0.13]	4.53	< .001	-	-	-
	Int. with Gender	0.06	0.04	[-0.02, 0.13]	1.52	.129	-	-	-
**Perceived Attraction from Match**									
Study 1	DV: Perceived Attraction from Match								
	Secondary Partner	0.19	0.07	[0.06, 0.32]	2.85	.005	129	137	1,439
	Gender (Women = 1)	-0.14	0.07	[-0.28, -0.01]	-2.11	.037	129	137	1,439
	Int. with Gender	0.12	0.07	[-0.01, 0.25]	1.85	.067	129	137	1,439
Study 2	DV: Perceived Attraction from Match								
	Secondary Partner	0.28	0.06	[0.18, 0.39]	5.15	< .001	156	164	2,402
	Gender (Women = 1)	-0.27	0.05	[-0.37, -0.17]	-5.31	< .001	156	164	2,402
	Int. with Gender	0.03	0.05	[-0.07, 0.14]	0.61	.544	156	164	2,402
Study 3	DV: Perceived Attraction from Match								
	Secondary Partner	0.06	0.07	[-0.08, 0.2]	0.85	.398	143	151	2,281
	Gender (Women = 1)	-0.21	0.07	[-0.36, -0.07]	-2.87	.005	143	151	2,281
	Int. with Gender	0.06	0.07	[-0.08, 0.2]	0.87	.383	143	151	2,281
Meta	DV: Perceived Attraction from Match								
	Secondary Partner	0.19	0.04	[0.12, 0.27]	5.38	< .001	-	-	-
	Gender (Women = 1)	-0.22	0.04	[-0.29, -0.15]	6.20	< .001	-	-	-
	Int. with Gender	0.07	0.04	[0, 0.14]	1.86	.063	-	-	-

Supplementary Table S6E

Continuous Regression Analyses and Meta-Analyses with Secondary Actor Effects and Moderation by Gender

Grouping and Study	Model	β	SE	95% CI	*t*	*p*	DF	*n*	Rows
**Later Interest (Continuous)**									
Study 3	DV: Later Interest								
	Primary Partner	0.07	0.04	[-0.02, 0.15]	1.57	.118	143	151	2,282
	Gender (Women = 1)	-0.35	0.08	[-0.50, -0.20]	-4.57	< .001	143	151	2,282
	Int. with Gender	-0.02	0.04	[-0.11, 0.06]	-0.58	.560	143	151	2,282
**Desire to Know Better**									
Study 1	DV: Desire to Know Better								
	Secondary Actor	-0.14	0.05	[-0.23, -0.04]	-2.87	.005	129	137	1,439
	Gender (Women = 1)	-0.17	0.06	[-0.30, -0.05]	-2.81	.006	129	137	1,439
	Int. with Gender	-0.07	0.05	[-0.17, 0.02]	-1.62	.108	129	137	1,439
Study 2	DV: Desire to Know Better								
	Secondary Actor	-0.04	0.04	[-0.11, 0.03]	-1.05	.293	156	164	2,402
	Gender (Women = 1)	-0.11	0.05	[-0.21, 0.00]	-1.99	.048	156	164	2,402
	Int. with Gender	0.03	0.04	[-0.04, 0.11]	0.94	.350	156	164	2,402
Meta	DV: Know Better								
	Secondary Actor	-0.08	0.03	[-0.13, -0.02]	2.60	.009	-	-	-
	Gender (Women = 1)	-0.14	0.04	[-0.22, -0.06]	3.34	< .001	-	-	-
	Int. with Gender	-0.01	0.03	[-0.06, 0.05]	0.29	.774	-	-	-
**Physical Attractiveness**									
Study 1	DV: Physically Attractive Rating								
	Secondary Actor	-0.20	0.06	[-0.31, -0.08]	-3.41	< .001	129	137	1,438
	Gender (Women = 1)	-0.24	0.07	[-0.38, -0.09]	-3.22	.002	129	137	1,438
	Int. with Gender	0.01	0.06	[-0.10, 0.12]	0.15	.879	129	137	1,438
Study 2	DV: Physically Attractive Rating								
	Secondary Actor	-0.17	0.06	[-0.28, -0.06]	-3.0	.003	156	164	2,400

	Gender (Women = 1)	-0.23	0.06	[-0.35, -0.10]	-3.61	< .001	156	164	2,400
	Int. with Gender	-0.01	0.06	[-0.12, 0.11]	-0.10	.924	156	164	2,400
Meta	DV: Physical Attractiveness								
	Secondary Actor	-0.18	0.04	[-0.26, -0.11]	4.54	< .001	-	-	-
	Gender (Women = 1)	-0.23	0.05	[-0.33, -0.14]	4.84	< .001	-	-	-
	Int. with Gender	0.00	0.04	[-0.08, 0.08]	0.04	.967	-	-	-
**Perceived Attraction from Match**									
Study 1	DV: Perceived Attraction from Match								
	Secondary Actor	-0.01	0.04	[-0.08, 0.07]	-0.23	.820	129	137	1,439
	Gender (Women = 1)	-0.18	0.07	[-0.32, -0.05]	-2.65	.009	129	137	1,439
	Int. with Gender	0.02	0.04	[-0.05, 0.10]	0.64	.521	129	137	1,439
Study 2	DV: Perceived Attraction from Match								
	Secondary Actor	-0.01	0.03	[-0.07, 0.05]	-0.29	.769	156	164	2,402
	Gender (Women = 1)	-0.26	0.05	[-0.37, -0.16]	-4.90	< .001	156	164	2,402
	Int. with Gender	-0.03	0.03	[-0.09, 0.03]	-0.90	.367	156	164	2,402
Study 3	DV: Perceived Attraction from Match								
	Secondary Actor	0.07	0.04	[-0.01, 0.15]	1.69	.092	143	151	2,281
	Gender (Women = 1)	-0.22	0.07	[-0.36, -0.07]	-2.97	.003	143	151	2,281
	Int. with Gender	-0.01	0.04	[-0.09, 0.07]	-0.27	.788	143	151	2,281
Meta	DV: Perceived Attraction from Match								
	Secondary Actor	0.01	0.02	[-0.03, 0.05]	0.53	.594	-	-	-
	Gender (Women = 1)	-0.23	0.04	[-0.30, -0.16]	6.24	< .001	-	-	-
	Int. with Gender	-0.01	0.02	[-0.05, 0.03]	0.37	.711	-	-	-

Supplementary Table S6F

Continuous Regression Analyses and Meta-Analyses with Secondary Relationship Effects and Moderation by Gender

Grouping and Study	Model	β	SE	95% CI	*t*	*p*	DF	*n*	Rows
**Later Interest (Continuous)**									
Study 3	DV: Later Interest								

	Primary Partner	0.02	0.05	[-0.07, 0.11]	0.36	.717	143	151	2,282
	Gender (Women = 1)	-0.35	0.08	[-0.50, -0.20]	-4.59	< .001	143	151	2,282
	Int. with Gender	-0.02	0.04	[-0.10, 0.07]	-0.35	.729	143	151	2,282
**Desire to Know Better**									
Study 1	DV: Desire to Know Better								
	Secondary Relationship	0.07	0.04	[-0.01, 0.15]	1.67	.096	129	137	1,439
	Gender (Women = 1)	-0.17	0.06	[-0.30, -0.05]	-2.73	.007	129	137	1,439
	Int. with Gender	0.03	0.04	[-0.05, 0.11]	0.80	.428	129	137	1,439
Study 2	DV: Desire to Know Better								
	Secondary Relationship	0.02	0.03	[-0.05, 0.08]	0.58	.562	156	164	2,402
	Gender (Women = 1)	-0.11	0.05	[-0.22, 0.00]	-2.04	.043	156	164	2,402
	Int. with Gender	-0.01	0.03	[-0.07, 0.06]	-0.25	.802	156	164	2,402
Meta	DV: Know Better								
	Secondary Relationship	0.04	0.03	[-0.01, 0.09]	1.50	.133	-	-	-
	Gender (Women = 1)	-0.14	0.04	[-0.22, -0.06]	3.32	< .001	-	-	-
	Int. with Gender	0.01	0.03	[-0.04, 0.06]	0.30	.765	-	-	-
**Physical Attractiveness**									
Study 1	DV: Physically Attractive Rating								
	Secondary Relationship	-0.03	0.04	[-0.11, 0.05]	-0.72	.473	129	137	1,438
	Gender	-0.22	0.07	[-0.37, -0.07]	-2.92	.004	129	137	1,438
	Int. with Gender	-0.02	0.04	[-0.10, 0.06]	-0.51	.612	129	137	1,438
Study 2	DV: Physically Attractive Rating								
	Secondary Relationship	0.00	0.04	[-0.07, 0.07]	0.0	1.000	156	164	2,400
	Gender (Women = 1)	-0.24	0.06	[-0.36, -0.11]	-3.66	< .001	156	164	2,400
	Int. with Gender	-0.03	0.04	[-0.10, 0.04]	-0.76	.449	156	164	2,400
Meta	DV: Physical Attractiveness								
	Secondary Relationship	-0.01	0.03	[-0.07, 0.04]	0.49	.627	-	-	-
	Gender (Women = 1)	-0.23	0.05	[-0.32, -0.13]	4.67	< .001	-	-	-
	Int. with Gender	-0.02	0.03	[-0.08, 0.03]	0.90	.366	-	-	-
**Perceived Attraction from Match**									
Study 1	DV: Perceived Attraction from Match								
	Secondary Relationship	0.12	0.03	[0.05, 0.19]	3.60	< .001	129	137	1,439

	Gender (Women = 1)	-0.17	0.07	[-0.30, -0.04]	-2.55	.012	129	137	1,439
	Int. with Gender	-0.05	0.03	[-0.12, 0.01]	-1.57	.118	129	137	1,439
Study 2	DV: Perceived Attraction from Match								
	Secondary Relationship	0.06	0.03	[0.00, 0.13]	1.95	.053	156	164	2,402
	Gender (Women = 1)	-0.27	0.05	[-0.38, -0.16]	-4.91	< .001	156	164	2,402
	Int. with Gender	0.05	0.03	[-0.01, 0.11]	1.65	.101	156	164	2,402
Study 3	DV: Perceived Attraction from Match								
	Secondary Relationship	0.04	0.04	[-0.04, 0.13]	0.97	.334	143	151	2,281
	Gender (Women = 1)	-0.22	0.07	[-0.36, -0.08]	-3.03	.003	143	151	2,281
	Int. with Gender	-0.08	0.04	[-0.16, 0.00]	-1.97	.051	143	151	2,281
Meta	DV: Perceived Attraction from Match								
	Secondary Relationship	0.08	0.02	[0.04, 0.12]	3.90	< .001	-	-	-
	Gender (Women = 1)	-0.23	0.04	[-0.30, -0.15]	6.20	< .001	-	-	-
	Int. with Gender	-0.02	0.02	[-0.06, 0.02]	0.85	.395	-	-	-

Supplementary Tables S6A-S6F. The tables show a summary of each multilevel continuous regression analysis performed testing for moderation by gender, as well as the meta-analyzed effect sizes (for a description of the analytic methods used see Supplementary Note 1, and for commentary on the results of these analyses see Supplementary Note 2). The Grouping and Study column shows the which variables were grouped together when meta-analyzed (in bold), and indicate which study the coefficients come from. Study 1 indicates the NSDS I (college sample), Study 2 indicates NSDS II (college sample), Study 3 indicates the Anime North Study (community sample), and Meta indicates the meta-analyzed effect sizes across the category. Model indicates the analysis variables: the variable labeled "DV" is the dependent variable, and the variables listed underneath are the independent variables. "Int. with Gender" indicates the interaction between gender and the SRM variable in the analysis. β indicates the slope of the predictor (the standardized beta-weight), and SE indicates the standard error of prediction. 95% CI indicates the upper and lower 95% confidence intervals (on the beta scale). *t* indicates the test statistic. *n* indicates how many unique respondents were available for each analysis. DF indicates the degrees of freedom used to calculate the *p* value for each analysis and was calculated as *n* minus one for each predictor (i.e., the one SRM independent variable) minus one for each of the four random effects (i.e., Participant ID, Partner ID, Participant ID nested within Partner ID, and Partner ID nested within Participant ID; see the Data Analysis section in the main manuscript) minus one. Rows indicates how many rows of data were used in the analysis. The *p* values listed are not corrected for multiple comparisons because the primary purpose of each analysis was to combine them using meta-analysis, and we focus our interpretation on the meta-analyzed effect sizes and confidence intervals. However, if the results from individual analyses are

interpreted, the appropriate Bonferroni correction to the alpha criterion for significance is $p < .008$ (calculated as .05 * 6 analyses per dependent variable per study).

Supplementary Table S7

Follow Up Multilevel Logistic Regressions and Meta-Analyses for the Simple Effects of Gender

Grouping and Study	DV	Gen.	Logit	SE	OR	95% CI	*Z*	*p*	DF	*n*	Rows
**Contact Initiation (Primary Actor)**											
Study 1	Contact Initiation	M	-0.14	0.18	0.87	[0.61, 1.24]	-0.78	0.438	67	73	206
Study 2	Contact Initiation††	M	-0.46	0.26	0.63	[0.38, 1.05]	-1.78	0.078	85	88	268
Study 3	Contact Initiation	M	0.21	0.24	1.24	[0.77, 1.97]	0.88	0.384	47	53	137
Meta	Contact Initiation	M	-0.12	0.13	0.89	[0.69, 1.13]	0.97	0.334	-	-	-
Study 1	Contact Initiation	W	0.38	1.10	1.47	[0.17, 12.63]	0.35	0.728	59	65	206
Study 2	Contact Initiation	W	-0.04	1.04	0.96	[0.13, 7.38]	-0.04	0.968	78	84	236
Study 3	Contact Initiation†	W	0.68	0.38	1.98	[0.94, 4.16]	1.78	0.082	44	48	145
Meta	Contact Initiation	W	0.57	0.34	1.78	[0.91, 3.47]	1.68	0.093	-	-	-
**Hangout or Correspond (Primary Partner)**											
Study 1	Hangout or Correspond	M	0.53	0.20	1.70	[1.15, 2.51]	2.70	0.008	60	66	623
Study 2	Correspond	M	0.35	0.16	1.42	[1.04, 1.94]	2.19	0.032	71	77	1,104
Study 2	Hangout	M	0.47	0.27	1.6	[0.94, 2.72]	1.76	0.082	71	77	1,100
Study 3	Interaction with Match	M	0.34	0.27	1.40	[0.83, 2.39]	1.24	0.220	54	60	935
Meta	Hangout or Correspond	M	0.42	0.10	1.52	[1.24, 1.86]	4.01	< .001	-	-	-
Study 1	Hangout or Correspond	W	-0.16	0.12	0.85	[0.67, 1.08]	-1.36	0.180	58	64	819
Study 2	Correspond	W	-0.15	0.16	0.86	[0.63, 1.18]	-0.91	0.366	70	76	1,299
Study 2	Hangout	W	-0.38	0.27	0.68	[0.40, 1.16]	-1.39	0.168	70	76	1,297
Study 3	Interaction with Match	W	0.87	0.25	2.39	[1.46, 3.90]	3.46	0.002	48	54	1,373
Meta	Hangout or Correspond	W	-0.06	0.08	0.94	[0.80, 1.11]	0.72	0.470	-	-	-
**Hangout or Correspond (Secondary Partner)**											
Study 1	Hangout or Correspond†	M	-0.02	0.21	0.98	[0.65, 1.48]	-0.11	0.912	60	64	623

Study 2	Correspond	M	-0.13	0.19	0.88	[0.61, 1.27]	-0.66	0.512	83	89	1,104
Study 2	Hangout†	M	-0.14	0.27	0.87	[0.51, 1.48]	-0.51	0.612	85	89	1,100
Study 3	Interaction with Match	M	0.47	0.30	1.60	[0.89, 2.88]	1.57	0.122	64	70	935
Meta	Hangout or Correspond	M	-0.01	0.12	0.99	[0.79, 1.24]	0.07	0.946	-	-	-
Study 1	Hangout or Correspond	W	0.36	0.18	1.43	[1.01, 2.04]	1.95	0.056	67	73	819
Study 2	Correspond	W	0.38	0.14	1.46	[1.11, 1.92]	2.71	0.008	69	75	1,299
Study 2	Hangout	W	0.22	0.27	1.24	[0.73, 2.12]	0.79	0.432	69	75	1,297
Study 3	Interaction with Match	W	0.55	0.24	1.74	[1.08, 2.77]	2.27	0.026	75	81	1,373
Meta	Hangout or Correspond	W	0.38	0.09	1.46	[1.21, 1.76]	4.01	< .001	-	-	-
**Later Romantic Interest (Binary) (Primary Partner)**											
Study 1	Later Romantic Interest	M	0.94	0.24	2.57	[1.60, 4.10]	3.87	< .001	60	66	623
Study 2	Later Romantic Interest	M	1.18	0.26	3.26	[1.96, 5.42]	4.56	< .001	71	77	1,104
Meta	Later Romantic Interest	M	1.05	0.18	2.87	[2.03, 4.07]	5.94	< .001	-	-	-
Study 1	Later Romantic Interest	W	0.37	0.33	1.45	[0.76, 2.76]	1.11	0.272	58	64	819
Study 2	Later Romantic Interest	W	0.91	0.22	2.49	[1.61, 3.82]	4.20	< .001	70	76	1,299
Meta	Later Romantic Interest	W	0.75	0.18	2.12	[1.48, 3.02]	4.12	< .001	-	-	-

Supplementary Tables S7. The tables show the simple effect analyses of each multilevel logistic regression that showed significant moderation by sex (see Supplementary Tables S5A-S5F; there were no multilevel continuous regressions that showed significant moderation by gender, see Supplementary Tables S6A-S6F) (for a description of the analytic methods used see Supplementary Note 1, and for commentary on the results of these analyses see Supplementary Note 2). The Grouping and Study column shows the which variables were grouped together when meta-analyzed (in bold), and indicate which study the coefficients come from. Study 1 indicates the NSDS I (college sample), Study 2 indicates NSDS II (college sample), Study 3 indicates the Anime North Study (community sample), and Meta indicates meta-analysis. The independent variable in each analysis is indicated in parentheses after the grouping variable. The dependent variable in each analysis is indicated in the DV column. "Gen." indicates the gender of the subjects used in each analysis ("M" indicates Men, "W" indicates Women). Logit indicates the slope of the predictor, and SE indicates the standard error of prediction (in logit units). OR indicates the slope in Odds Ratio. 95% CI indicates the upper and lower 95% confidence intervals (in odds ratio units). *Z* indicates the test statistic. *n* indicates how many unique respondents were available for each analysis. DF indicates the degrees of freedom used to calculate the *p* value for each analysis and was calculated as *n* minus one for each predictor (i.e., the one SRM independent variable) minus one for each of the four random effects (i.e., Participant ID, Partner ID,

Participant ID nested within Partner ID, and Partner ID nested within Participant ID; however, in two cases only Participant ID and Partner ID were included as random effects, see note below†) minus one. Rows indicates how many rows of data were used in the analysis. The *p* values listed are not corrected for multiple comparisons because the primary purpose of each analysis was to combine them using meta-analysis, and we focus our interpretation on the meta-analyzed effect sizes and confidence intervals. However, if any individual result is interpreted, the appropriate Bonferroni correction to the alpha criterion for significance is $p < .008$ (calculated as .05 * 6 analyses per dependent variable per study).

† For the analyses marked with the dagger symbol above, some random effects were removed from the model because when they were included the models produced implausibly low standard error estimates (in turn producing implausibly large *Z* test statistics). The models marked with one dagger (†) indicate that the nested random effects were removed (Participant ID within Partner ID, and Partner ID within Participant ID) and Participant ID and Partner ID were kept in the model, and the models marked with two daggers (††) indicate that the nested random effects and Partner ID were removed from the model and Participant ID was kept in the model. Removing random effects from these models resulted in more plausible standard error estimates. The original models with the nested random effects are available upon request.

Supplementary Table S8

Correlations Between Primary and Secondary SRM Effects

	Primary Partner	Primary Actor	Primary Relationship	Secondary Partner	Secondary Actor	Secondary Relationship
**Study 1†**						
Primary Partner	-					
Primary Actor	0.00	-				
Primary Relationship	0.00	0.00	-			
Secondary Partner	0.00	**-0.28**	0.00	-		
Secondary Actor	**-0.28**	0.00	0.00	0.00	-	
Secondary Relationship	0.00	0.00	**0.14**	0.00	0.00	-
**Study 2††**						
Primary Partner	-					
Primary Actor	0.00	-				
Primary Relationship	0.00	0.00	-			
Secondary Partner	-0.01	**-0.18**	0.00	-		
Secondary Actor	**-0.18**	0.00	0.00	0.00	-	
Secondary Relationship	0.00	0.00	**0.15**	0.00	0.00	-
**Study 3**						
Primary Partner	-					
Primary Actor	0.00	-				
Primary Relationship	0.00	0.00	-			
Secondary Partner	0.00	**-0.10**	0.00	-		
Secondary Actor	**-0.10**	0.00	0.00	0.00	-	
Secondary Relationship	0.00	0.00	**0.08**	0.00	0.00	-

Supplementary Table S8. The table shows the Pearson correlations between the primary and secondary SRM effects in initial desire (determined from the speed-dating event) in each study. Bolded values indicate the association is significant ($p < .001$). Note, in each study, the correlation between Primary Partner and Secondary Actor is the same as the correlation between Primary Actor and Secondary Partner, as these correlations are reciprocal.

† For Study 1, these correlations were assessed and reported previously in Eastwick, Finkel, Mochon and Ariely (14). There are minor differences in the correlations reported here due to the previous study using a slightly smaller subset of the available data, but the associations are in the same direction.

†† The SRM calculations yield perfectly uncorrelated estimates for each participant's partner, actor, and relationship effects (i.e., within the primary SRM effects and within the secondary SRM effects, Pearson r is 0.00). This also means that primary partner effects and secondary partner effects should be completely uncorrelated (as secondary partner effects is the reciprocal of primary actor effects, and primary actor effects is not correlated with primary partner effects). Although this is the case for Study 1 and Study 3, in Study 3, primary partner and secondary partner effects had a non-0 correlation (r = -0.01). The reason this correlation was not completely 0 in Study 2 was because the correlations reported in the table represent the overall correlation

across the entire sample, which included one all-men speed-dating sample which used a modified version of the SRM to calculate partner, actor, and relationship effects in a round-robin design (with a slight correction to account for the fact that each participant rated their desire for all other participants but did not rate their own desire for themselves). For more information, see (15)

Supplementary Tables S9A-S9F

These tables show the sensitivity analyses in which each original multilevel logistic regression was repeated with random slopes (within participant ID and partner ID) included in the model. The tables also report the meta-analyzed coefficients for each grouping of variables. See below for a combined table caption.

Supplementary Table S9A

Multilevel Logistic Regressions and Meta-Analyses for Primary Partner Effects with Random Slopes Included in the Model

		Random Slopes Model									Original Model	
Grouping and Study	DV	Logit	SE	OR	95% CI	*Z*	*p*	DF	*n*	Rows	OR	*p*
**Contact Initiation**												
Study 1	Contact Initiation	0.58	0.20	1.79	[1.20, 2.68]	2.86	.005	128	138	412	1.98	< .001
Study 2	Contact Initiation	0.62	0.31	1.86	[1.02, 3.4]	2.03	.044	162	172	504	1.90	< .001
Study 3	Contact Initiation†	0.34	0.74	1.41	[0.33, 6.07]	0.47	.642	95	101	282	1.41	.642
Meta	Contact Initiation	0.58	0.17	1.79	[1.29, 2.47]	3.52	< .001	-	-	-	1.92	< .001
**Hangout or Correspond**												
Study 1	Hangout or Correspond	0.22	0.12	1.24	[0.98, 1.57]	1.85	.067	120	130	1,442	1.15	.204
Study 2	Correspond	0.10	0.14	1.10	[0.83, 1.47]	0.69	.492	143	153	2,403	1.06	.596
Study 2	Hangout	0.0	0.22	1.0	[0.65, 1.55]	0.01	.992	143	153	2,397	0.99	.955
Study 3	Interaction with Match	0.59	0.20	1.80	[1.22, 2.66]	3.01	.003	104	114	2,308	1.73	.005
Meta	Hangout or Correspond	0.22	0.08	1.24	[1.07, 1.44]	2.78	.005	-	-	-	1.15	.032
**Later Romantic Interest (Binary)**												
Study 1	Later Romantic Interest	1.11	0.30	3.04	[1.69, 5.49]	3.74	< .001	120	130	1,442	2.06	< .001
Study 2	Later Romantic Interest	1.08	0.17	2.94	[2.09, 4.13]	6.26	< .001	143	153	2,403	2.85	< .001
Meta	Later Romantic Interest	1.09	0.15	2.96	[2.21, 3.97]	7.29	< .001	-	-	-	2.45	< .001

Supplementary Table S9B

Multilevel Logistic Regressions and Meta-Analyses for Primary Actor Effects with Random Slopes Included in the Model

Grouping and Study	DV	Random Slopes Model									Original Model	
		Logit	SE	OR	95% CI	*Z*	*p*	DF	*n*	Rows	OR	*p*
**Contact Initiation**												
Study 1	Contact Initiation	0.03	0.25	1.03	[0.62, 1.70]	0.12	.908	128	138	412	1.05	.790
Study 2	Contact Initiation	-0.10	0.40	0.91	[0.41, 2.02]	-0.24	.813	162	172	504	0.83	.443
Study 3	Contact Initiation	-0.44	1.24	0.64	[0.05, 7.54]	-0.36	.723	91	101	282	1.46	.176
Meta	Contact Initiation	-0.02	0.21	0.98	[0.65, 1.49]	0.09	.930	-	-	-	1.05	.683
**Hangout or Correspond**												
Study 1	Hangout or Correspond	-0.19	0.14	0.83	[0.63, 1.09]	-1.37	.174	120	130	1,442	0.86	.263
Study 2	Correspond	-0.24	0.12	0.79	[0.63, 1.00]	-2.01	.046	143	153	2,403	0.78	.024
Study 2	Hangout	-0.04	0.23	0.96	[0.61, 1.50]	-0.17	.862	143	153	2,397	0.86	.426
Study 3	Interaction with Match	0.08	0.26	1.08	[0.64, 1.82]	0.29	.770	104	114	2,308	1.05	.818
Meta	Hangout or Correspond	-0.17	0.08	0.85	[0.72, 0.99]	2.12	.034	-	-	-	0.84	.019
**Later Romantic Interest (Binary)**												
Study 1	Later Romantic Interest	0.20	0.25	1.22	[0.75, 1.99]	0.83	.411	120	130	1,442	1.16	.471
Study 2	Later Romantic Interest	0.06	0.20	1.06	[0.72, 1.56]	0.28	.777	143	153	2,403	1.11	.537
Meta	Later Romantic Interest	0.11	0.15	1.12	[0.83, 1.51]	0.74	.461	-	-	-	1.13	.347

Supplementary Table S9C

Multilevel Logistic Regressions and Meta-Analyses for Primary Relationship Effects with Random Slopes Included in the Model

Grouping and Study	DV	Random Slopes Model									Original Model	
		Logit	SE	OR	95% CI	*Z*	*p*	DF	*n*	Rows	OR	*p*
**Contact Initiation**												
Study 1	Contact Initiation	0.61	0.23	1.84	[1.16, 2.93]	2.61	.010	128	138	412	1.44	.025
Study 2	Contact Initiation	0.96	0.41	2.62	[1.17, 5.86]	2.35	.020	162	172	504	1.83	.007
Study 3	Contact Initiation†	0.58	0.91	1.78	[0.29, 10.89]	0.63	.529	92	101	282	1.42	.650
Meta	Contact Initiation	0.69	0.20	2.0	[1.35, 2.95]	3.49	< .001	-	-	-	1.56	< .001
**Hangout or Correspond**												

Study 1	Hangout or Correspond	0.29	0.11	1.33	[1.06, 1.67]	2.49	.014	120	130	1,442	1.27	.014
Study 2	Correspond	0.35	0.12	1.42	[1.12, 1.81]	2.86	.005	143	153	2,403	1.49	< .001
Study 2	Hangout	0.66	0.26	1.93	[1.16, 3.22]	2.54	.012	143	153	2,397	1.66	.010
Study 3	Interaction with Match	0.40	0.20	1.49	[1.01, 2.20]	2.03	.045	104	114	2,308	1.69	.002
Meta	Hangout or Correspond	0.36	0.07	1.43	[1.23, 1.65]	4.81	< .001	-	-	-	1.43	< .001
**Later Romantic Interest (Binary)**												
Study 1	Later Romantic Interest	0.68	0.23	1.98	[1.27, 3.10]	3.02	.003	120	130	1,442	1.69	.004
Study 2	Later Romantic Interest	0.70	0.18	2.01	[1.40, 2.89]	3.84	< .001	143	153	2,403	1.78	< .001
Meta	Later Romantic Interest	0.69	0.14	2.0	[1.51, 2.64]	4.89	< .001	-	-	-	1.75	< .001

Supplementary Table S9D

Multilevel Logistic Regressions and Meta-Analyses for Secondary Partner Effects with Random Slopes Included in the Model

		Random Slopes Model									Original Model	
Grouping and Study	DV	Logit	SE	OR	95% CI	*Z*	*p*	DF	*n*	Rows	OR	*p*
**Contact Initiation**												
Study 1	Contact Initiation	-0.92	0.30	0.40	[0.22, 0.72]	-3.10	.002	128	138	412	0.54	< .001
Study 2	Contact Initiation	-0.57	0.58	0.57	[0.18, 1.78]	-0.98	.328	162	172	504	0.72	.154
Study 3	Contact Initiation	-0.14	0.83	0.87	[0.17, 4.46]	-0.17	.862	130	140	282	0.97	.973
Meta	Contact Initiation	-0.78	0.25	0.46	[0.28, 0.75]	3.11	.002	-	-	-	0.61	< .001
**Hangout or Correspond**												
Study 1	Hangout or Correspond	0.19	0.14	1.21	[0.91, 1.6]	1.33	.186	127	137	1,442	1.22	.156
Study 2	Correspond	0.22	0.16	1.25	[0.92, 1.69]	1.42	.158	154	164	2,403	1.11	.399
Study 2	Hangout	0.19	0.24	1.21	[0.75, 1.95]	0.80	.428	154	164	2,397	0.99	.974
Study 3	Interaction with Match	0.59	0.23	1.80	[1.15, 2.83]	2.57	.011	141	151	2,308	1.69	.007
Meta	Hangout or Correspond	0.26	0.09	1.30	[1.09, 1.54]	2.93	.003	-	-	-	1.20	.018
**Later Romantic Interest (Binary)**												
Study 1	Later Romantic Interest	-0.45	0.27	0.63	[0.37, 1.08]	-1.70	.091	127	137	1,442	0.83	.366
Study 2	Later Romantic Interest	0.24	0.25	1.27	[0.78, 2.07]	0.96	.340	154	164	2,403	1.39	.063
Meta	Later Romantic Interest	-0.08	0.18	0.92	[0.65, 1.31]	0.46	.649	-	-	-	1.12	.398

Supplementary Table S9E

Multilevel Logistic Regressions and Meta-Analyses for Secondary Actor Effects with Random Slopes Included in the Model

		Random Slopes Model									Original Model	
Grouping and Study	DV	Logit	SE	OR	95% CI	*Z*	*p*	DF	*n*	Rows	OR	*p*
**Contact Initiation**												
Study 1	Contact Initiation	0.11	0.27	1.12	[0.66, 1.92]	0.42	.674	128	138	412	0.89	.506
Study 2	Contact Initiation††	0.38	0.30	1.46	[0.80, 2.64]	1.24	.215	166	172	504	1.05	.782
Study 3	Contact Initiation†	0.20	0.22	1.22	[0.79, 1.88]	0.89	.375	134	140	282	1.22	.375
Meta	Contact Initiation	0.22	0.15	1.24	[0.93, 1.66]	1.45	.148	-	-	-	1.02	.849
**Hangout or Correspond**												
Study 1	Hangout or Correspond	-0.07	0.11	0.94	[0.75, 1.17]	-0.58	.563	127	137	1,442	0.89	.259
Study 2	Correspond	-0.21	0.11	0.81	[0.65, 1.01]	-1.85	.066	154	164	2,403	0.81	.050
Study 2	Hangout	0.02	0.24	1.02	[0.64, 1.64]	0.09	.925	154	164	2,397	1.01	.941
Study 3	Interaction with Match	0.48	0.27	1.62	[0.95, 2.76]	1.80	.074	141	151	2,308	1.35	.094
Meta	Hangout or Correspond	-0.08	0.07	0.93	[0.80, 1.07]	1.07	.285	-	-	-	0.92	.208
**Later Romantic Interest (Binary)**												
Study 1	Later Romantic Interest	-0.14	0.27	0.87	[0.51, 1.49]	-0.51	.609	127	137	1,442	0.67	.039
Study 2	Later Romantic Interest	-0.30	0.20	0.74	[0.50, 1.09]	-1.54	.125	154	164	2,403	0.76	.122
Meta	Later Romantic Interest	-0.25	0.16	0.78	[0.57, 1.07]	1.55	.121	-	-	-	0.72	.011

Supplementary Table S9F

Multilevel Logistic Regressions and Meta-Analyses for Secondary Relationship Effects with Random Slopes Included in the Model

		Random Slopes Model									Original Model	
Grouping and Study	DV	Logit	SE	OR	95% CI	*Z*	*p*	DF	*n*	Rows	OR	*p*
**Contact Initiation**												
Study 1	Contact Initiation	0.07	0.25	1.07	[0.65, 1.74]	0.27	.788	128	138	412	1.08	.592

Study 2	Contact Initiation	0.02	0.77	1.02	[0.22, 4.68]	0.02	.984	162	172	504	1.06	.716
Study 3	Contact Initiation†	0.09	0.21	1.09	[0.72, 1.65]	0.41	.680	134	140	282	1.09	.680
Meta	Contact Initiation	0.08	0.16	1.08	[0.79, 1.46]	0.48	.629	-	-	-	1.08	.448
**Hangout or Correspond**												
Study 1	Hangout or Correspond	0.36	0.11	1.43	[1.15, 1.78]	3.21	.002	127	137	1,442	1.34	.002
Study 2	Correspond	0.51	0.13	1.67	[1.29, 2.15]	3.92	< .001	154	164	2,403	1.64	< .001
Study 2	Hangout	0.53	0.27	1.70	[1.00, 2.89]	1.99	.048	154	164	2,397	1.60	.019
Study 3	Interaction with Match	0.39	0.21	1.48	[0.98, 2.24]	1.87	.063	141	151	2,308	1.42	.049
Meta	Hangout or Correspond	0.43	0.08	1.53	[1.32, 1.77]	5.67	< .001	-	-	-	1.47	< .001
**Later Romantic Interest (Binary)**												
Study 1	Later Romantic Interest	0.14	0.27	1.15	[0.67, 1.97]	0.51	.610	127	137	1,442	1.33	.103
Study 2	Later Romantic Interest	0.37	0.19	1.45	[0.99, 2.11]	1.95	.053	154	164	2,403	1.19	.217
Meta	Later Romantic Interest	0.30	0.16	1.34	[0.99, 1.82]	1.89	.059	-	-	-	1.24	.046

Supplementary Tables S9A-S9F. The tables show a summary of each follow up multilevel logistic regression analysis performed with random slopes for the SRM variable included in the model (within Participant ID and Partner ID), as well as the meta-analyzed effect size within each grouping of variables. The Grouping and Study column shows the which variables were grouped together when meta-analyzed (in bold) and indicate which study the coefficients come from. Study 1 indicates the NSDS I (college sample), Study 2 indicates NSDS II (college sample), and Study 3 indicates the Anime North Study (community sample). The IV in each analysis is indicated in the title of each table, and the dependent variable in each analysis is indicated in the DV column. Logit indicates the slope of the predictor, and SE indicates the standard error of prediction (in logit units). OR indicates the slope in Odds Ratio. 95% CI indicates the upper and lower 95% confidence intervals (in odds ratio units). *Z* indicates the test statistic. *n* indicates how many unique respondents were available for each analysis. DF indicates the degrees of freedom used to calculate the *p* value for each analysis and was calculated as *n* minus one for each predictor (i.e., the one SRM independent variable) minus one for each of the four random effects (i.e., Participant ID, Partner ID, Participant ID nested within Partner ID, and Partner ID nested within Participant ID) minus four for the two random slopes included (SRM Variable within Participant ID, SRM Variable within Partner ID, and the correlation between the SRM Variable and both Participant ID and Partner ID) minus one (see footnotes below for a few exceptions). Rows indicates how many rows of data were used in the analysis. The *p* values listed are not corrected for multiple comparisons because the primary purpose of each analysis was to combine them using meta-analysis, and we focus our interpretation on the meta-analyzed effect sizes and confidence intervals. However, if any individual result is interpreted, the appropriate Bonferroni correction to the alpha criterion for significance is $p < .008$ (calculated as .05 * 6 analyses per dependent variable per study). For convenience, the OR and *p* values for the original analysis (i.e., the models without random slopes for the SRM variables included in the models) are also

reported in the table (for more information about the original analyses, see Supplementary Tables S2A-S2F and Table 1 in the main manuscript).

† For the Study 3 Contact Initiation analyses involving Primary Partner effects, Primary Relationship effects, Secondary Actor effects, and Secondary Relationship effects, the random slopes were dropped from the model because including them in the model yielded improbably large OR, SE, and 95% CI ranges. These models included the following random effects: Participant ID, Partner ID, Participant ID nested within Partner ID, and Partner ID nested within Participant ID.

†† For the Study 2 Contact Initiation analysis involving Secondary Actor effects, the full suite of random effects produced improbably extreme OR, SE, and 95% CI estimates. For this model, we included Participant ID and Partner ID as random effects and Secondary Actor effects as a random slope (within Participant ID only).

Supplementary Tables S10A-S10F

These tables show the sensitivity analyses in which each original multilevel continuous regression was repeated with random slopes (within participant ID and partner ID) included in the model. The tables also report the meta-analyzed coefficients for each grouping of variables. See below for a combined table caption.

Supplementary Table S10A

Multilevel Continuous Regressions and Meta-Analyses for Primary Partner Effects with Random Slopes Included in the Model

		Random Slopes Model								Original Model	
Grouping and Study	DV	b	SE	95% CI	*t*	*p*	DF	*n*	Rows	OR	*p*
**Later Interest (Continuous)**											
Study 3	Later Interest	0.19	0.05	[0.08, 0.29]	3.61	< .001	94	104	2,282	0.17	< .001
**Know Better**											
Study 1	Desire to Know Better	0.26	0.05	[0.17, 0.35]	5.60	< .001	120	130	1,439	0.26	< .001
Study 2	Desire to Know Better	0.20	0.03	[0.13, 0.27]	5.72	< .001	143	153	2,402	0.20	< .001
Meta	Desire to Know Better	0.22	0.03	[0.17, 0.28]	7.94	< .001	-	-	-	0.22	< .001
**Physical Attractiveness**											
Study 1	Physically Attractive Rating	0.47	0.04	[0.38, 0.55]	10.80	< .001	120	130	1,438	0.47	< .001
Study 2	Physically Attractive Rating	0.45	0.05	[0.35, 0.55]	9.13	< .001	143	153	2,400	0.48	< .001
Meta	Physical Attractiveness	0.46	0.03	[0.40, 0.52]	14.14	< .001	-	-	-	0.48	< .001
**Perceived Attraction from Match**											
Study 1	Perceived Attraction	0.02	0.04	[-0.06, 0.10]	0.47	.642	120	130	1,439	0.01	.767
Study 2	Perceived Attraction	0.03	0.04	[-0.05, 0.11]	0.80	.422	143	153	2,402	0.02	.593
Study 3	Perceived Attraction	0.08	0.05	[-0.02, 0.18]	1.68	.097	94	104	2,281	0.06	.145
Meta	Perceived Attraction	0.04	0.02	[-0.01, 0.09]	1.61	.107	-	-	-	0.03	.210

Supplementary Table S10B

Multilevel Continuous Regressions and Meta-Analyses for Primary Actor Effects with Random Slopes Included in the Model

Grouping and Study	DV	Random Slopes Model b	SE	95% CI	*t*	*p*	DF	*n*	Rows	Original Model b	*p*
**Later Interest (Continuous)**											
Study 3	Later Interest	0.43	0.07	[0.28, 0.57]	5.75	< .001	94	104	2,282	0.37	< .001
**Know Better**											
Study 1	Desire to Know Better	0.15	0.05	[0.05, 0.26]	2.83	.005	120	130	1,439	0.14	.011
Study 2	Desire to Know Better	0.08	0.05	[-0.02, 0.18]	1.50	.136	143	153	2,402	0.07	.158
Meta	Desire to Know Better	0.11	0.04	[0.04, 0.18]	3.02	.002	-	-	-	0.10	.005
**Physical Attractiveness**											
Study 1	Physically Attractive Rating	0.25	0.06	[0.14, 0.36]	4.49	< .001	120	130	1,438	0.23	< .001
Study 2	Physically Attractive Rating	0.13	0.05	[0.04, 0.22]	2.92	.004	143	153	2,400	0.12	.004
Meta	Physical Attractiveness	0.18	0.04	[0.11, 0.25]	5.10	< .001	-	-	-	0.16	< .001
**Perceived Attraction from Match**											
Study 1	Perceived Attraction	-0.07	0.07	[-0.21, 0.06]	-1.14	.257	120	130	1,439	-0.05	.463
Study 2	Perceived Attraction	0.01	0.06	[-0.11, 0.12]	0.11	.911	143	153	2,402	-0.01	.903
Study 3	Perceived Attraction	0.33	0.07	[0.19, 0.48]	4.54	< .001	94	104	2,281	0.28	< .001
Meta	Perceived Attraction	0.07	0.04	[-0.01, 0.14]	1.77	.076	-	-	-	0.05	.147

Supplementary Table S10C

Multilevel Continuous Regressions and Meta-Analyses for Primary Relationship Effects with Random Slopes Included in the Model

Grouping and Study	DV	Random Slopes Model b	SE	95% CI	*t*	*p*	DF	*n*	Rows	Original Model b	*p*
**Later Interest (Continuous)**											
Study 3	Later Interest	0.16	0.06	[0.05, 0.27]	2.88	.005	94	104	2,282	0.19	< .001
**Know Better**											
Study 1	Desire to Know Better	0.11	0.04	[0.02, 0.19]	2.43	.017	120	130	1,439	0.11	.007
Study 2	Desire to Know Better	0.10	0.03	[0.03, 0.16]	2.82	.005	143	153	2,402	0.10	.001
Meta	Desire to Know Better	0.10	0.03	[0.05, 0.15]	3.72	< .001	-	-	-	0.11	< .001

**Physical Attractiveness**											
Study 1	Physically Attractive Rating	0.19	0.04	[0.11, 0.26]	4.98	< .001	120	130	1,438	0.19	< .001
Study 2	Physically Attractive Rating	0.18	0.04	[0.11, 0.25]	4.86	< .001	143	153	2,400	0.18	< .001
Meta	Physical Attractiveness	0.18	0.03	[0.13, 0.23]	6.95	< .001	-	-	-	0.18	< .001
**Perceived Attraction from Match**											
Study 1	Perceived Attraction	0.02	0.03	[-0.04, 0.09]	0.70	.483	120	130	1,439	0.02	.589
Study 2	Perceived Attraction	0.01	0.03	[-0.06, 0.08]	0.19	.850	143	153	2,402	0.0	.961
Study 3	Perceived Attraction	0.10	0.04	[0.01, 0.18]	2.16	.033	94	104	2,281	0.09	.042
Meta	Perceived Attraction	0.03	0.02	[-0.01, 0.08]	1.59	.111	-	-	-	0.03	.181

Supplementary Table S10D

Multilevel Continuous Regressions and Meta-Analyses for Secondary Partner Effects with Random Slopes Included in the Model

		Random Slopes Model								Original Model	
Grouping and Study	DV	b	SE	95% CI	*t*	*p*	DF	*n*	Rows	b	*p*
**Later Interest (Continuous)**											
Study 3	Later Interest	-0.04	0.09	[-0.23, 0.14]	-0.46	.645	141	151	2,282	-0.01	.900
**Know Better**											
Study 1	Desire to Know Better	-0.10	0.06	[-0.22, 0.01]	-1.72	.087	127	137	1,439	-0.10	.092
Study 2	Desire to Know Better	0.00	0.05	[-0.11, 0.11]	-0.01	.994	154	164	2,402	0.01	.822
Meta	Desire to Know Better	-0.05	0.04	[-0.13, 0.03]	1.18	.236	-	-	-	-0.04	.340
**Physical Attractiveness**											
Study 1	Physically Attractive Rating	-0.05	0.06	[-0.17, 0.07]	-0.81	.418	127	137	1,438	-0.04	.481
Study 2	Physically Attractive Rating	-0.01	0.05	[-0.11, 0.08]	-0.27	.787	154	164	2,400	0.0	.972
Meta	Physical Attractiveness	-0.03	0.04	[-0.10, 0.05]	0.72	.470	-	-	-	-0.01	.691
**Perceived Attraction from Match**											
Study 1	Perceived Attraction	0.19	0.07	[0.05, 0.32]	2.76	.007	127	137	1,439	0.19	.006
Study 2	Perceived Attraction	0.27	0.06	[0.16, 0.38]	4.72	< .001	154	164	2,402	0.26	< .001
Study 3	Perceived Attraction	0.04	0.08	[-0.13, 0.21]	0.47	.638	141	151	2,281	0.07	.331
Meta	Perceived Attraction	0.19	0.04	[0.12, 0.27]	5.0	< .001	-	-	-	0.19	< .001

Supplementary Table S10E

Multilevel Continuous Regressions and Meta-Analyses for Secondary Actor Effects with Random Slopes Included in the Model

		Random Slopes Model								Original Model	
Grouping and Study	DV	b	SE	95% CI	*t*	*p*	DF	*n*	Rows	b	*p*
**Later Interest (Continuous)**											
Study 3	Later Interest	0.08	0.07	[-0.05, 0.21]	1.21	.226	141	151	2,282	0.08	.080
**Know Better**											
Study 1	Desire to Know Better	-0.10	0.05	[-0.20, -0.01]	-2.09	.038	127	137	1,439	-0.11	.021
Study 2	Desire to Know Better	-0.04	0.04	[-0.12, 0.03]	-1.17	.242	154	164	2,402	-0.05	.198
Meta	Desire to Know Better	-0.07	0.03	[-0.12, -0.01]	2.20	.028	-	-	-	-0.07	.014
**Physical Attractiveness**											
Study 1	Physically Attractive Rating	-0.20	0.06	[-0.32, -0.09]	-3.55	< .001	127	137	1,438	-0.19	.001
Study 2	Physically Attractive Rating	-0.18	0.06	[-0.29, -0.06]	-3.09	.002	154	164	2,400	-0.18	.002
Meta	Physical Attractiveness	-0.19	0.04	[-0.27, -0.11]	4.69	< .001	-	-	-	-0.19	< .001
**Perceived Attraction from Match**											
Study 1	Perceived Attraction	-0.01	0.04	[-0.09, 0.07]	-0.32	.750	127	137	1,439	-0.01	.772
Study 2	Perceived Attraction	0.01	0.03	[-0.06, 0.08]	0.23	.815	154	164	2,402	-0.01	.838
Study 3	Perceived Attraction	0.09	0.05	[-0.01, 0.19]	1.80	.074	141	151	2,281	0.08	.064
Meta	Perceived Attraction	0.02	0.02	[-0.03, 0.06]	0.80	.424	-	-	-	0.01	.511

Supplementary Table S10F

Multilevel Continuous Regressions and Meta-Analyses for Secondary Relationship Effects with Random Slopes Included in the Model

		Random Slopes Model								Original Model	
Grouping and Study	DV	b	SE	95% CI	*t*	*p*	DF	*n*	Rows	b	*p*
**Later Interest (Continuous)**											
Study 3	Later Interest	0.03	0.05	[-0.07, 0.13]	0.62	.538	141	151	2,282	0.03	.578

**Know Better**											
Study 1	Desire to Know Better	0.08	0.04	[-0.01, 0.16]	1.74	.085	127	137	1,439	0.07	.112
Study 2	Desire to Know Better	0.02	0.03	[-0.04, 0.09]	0.72	.472	154	164	2,402	0.02	.481
Meta	Desire to Know Better	0.04	0.03	[-0.01, 0.09]	1.62	.105	-	-	-	0.04	.124
**Physical Attractiveness**											
Study 1	Physically Attractive Rating	-0.02	0.04	[-0.10, 0.06]	-0.46	.644	127	137	1,438	-0.02	.590
Study 2	Physically Attractive Rating	0.01	0.04	[-0.06, 0.09]	0.34	.734	154	164	2,400	0.01	.839
Meta	Physical Attractiveness	0.0	0.03	[-0.06, 0.05]	0.06	.953	-	-	-	-0.01	.838
**Perceived Attraction from Match**											
Study 1	Perceived Attraction	0.12	0.04	[0.05, 0.20]	3.16	.002	127	137	1,439	0.12	< .001
Study 2	Perceived Attraction	0.06	0.03	[0.00, 0.13]	1.94	.054	154	164	2,402	0.06	.080
Study 3	Perceived Attraction	0.07	0.05	[-0.02, 0.16]	1.61	.110	141	151	2,281	0.06	.142
Meta	Perceived Attraction	0.08	0.02	[0.04, 0.13]	3.85	< .001	-	-	-	0.08	< .001

Supplementary Tables S10A-S10F. The tables show a summary of each follow up multilevel continuous regression analysis performed with random slopes for the SRM variable included in the model (within Participant ID and Partner ID), as well as the meta-analyzed effect size within each grouping of variables. The Grouping and Study column shows the which variables were grouped together when meta-analyzed (in bold) and indicate which study the coefficients come from. Study 1 indicates the NSDS I (college sample), Study 2 indicates NSDS II (college sample), and Study 3 indicates the Anime North Study (community sample). The IV in each analysis is indicated in the title of each table, and the dependent variable in each analysis is indicated in the DV column. β indicates the slope of the standardized beta estimate, and SE indicates the standard error of prediction. 95% CI indicates the upper and lower 95% confidence intervals (on the beta scale). *t* indicates the test statistic. *n* indicates how many unique respondents were available for each analysis. DF indicates the degrees of freedom used to calculate the *p* value for each analysis and was calculated as *n* minus one for each predictor (i.e., the one SRM independent variable) minus one for each of the four random effects (i.e., Participant ID, Partner ID, Participant ID nested within Partner ID, and Partner ID nested within Participant ID) minus four for the two random slopes included (SRM Variable within Participant ID, SRM Variable within Partner ID, and the correlation between the SRM Variable and both Participant ID and Partner ID) minus one. Rows indicates how many rows of data were used in the analysis. The *p* values listed are not corrected for multiple comparisons because the primary purpose of each analysis was to combine them using meta-analysis, and we focus our interpretation on the meta-analyzed effect sizes and confidence intervals. However, if any individual result is interpreted, the appropriate Bonferroni correction to the alpha criterion for significance is $p < .008$ (calculated as .05 * 6 analyses per dependent variable per study). For convenience, the Beta and *p* values for the original analysis (i.e., the models without random

slopes for the SRM variables included in the models) are also reported in the table (for more information about the original analyses, see Supplementary Tables S3A-S3F and Tables 2-3 in the main manuscript).

Supplementary Tables S11A-S11C

These tables show the sensitivity analyses in which the original multilevel logistic regression analyses were repeated with all six Primary and Secondary SRM effect included in each model as simultaneous predictors of the dichotomous outcome variable (i.e., maximal models). The tables also report the meta-analyzed coefficients for each grouping of variables. See below for a combined table caption. Note, because six predictors were included in each model at a time, these supplementary tables are organized by outcome variables (i.e., each table shows the results for a different grouping of outcome variables) rather than by SRM effect (as all the other results tables are).

Supplementary Table S11A

Multilevel Logistic Regressions and Meta-Analyses for Maximal Models Predicting Contact Initiation

	Maximal Model									Original Model	
Study and IV	Logit	SE	OR	95% CI	Z	*p*	DF	*n*	Rows	OR	*p*
**Study 1**											
Primary Partner	0.97	0.24	2.63	[1.65, 4.19]	4.10	< .001	127	138	412	1.98	< .001
Primary Actor	0.09	0.20	1.10	[0.73, 1.64]	0.46	.649	127	138	412	1.05	.790
Primary Relationship	0.56	0.18	1.74	[1.22, 2.50]	3.06	.003	127	138	412	1.44	.025
Secondary Partner	-0.75	0.21	0.47	[0.31, 0.72]	-3.57	< .001	127	138	412	0.54	< .001
Secondary Actor	0.01	0.19	1.01	[0.69, 1.48]	0.05	.964	127	138	412	0.89	.506
Secondary Relationship	-0.02	0.16	0.98	[0.71, 1.35]	-0.13	.894	127	138	412	1.08	.592
**Study 2**											
Primary Partner	1.04	0.27	2.83	[1.65, 4.85]	3.81	< .001	161	172	504	1.90	< .001
Primary Actor	0.01	0.30	1.01	[0.56, 1.82]	0.02	.982	161	172	504	0.83	.443
Primary Relationship	0.89	0.26	2.42	[1.45, 4.05]	3.40	< .001	161	172	504	1.83	.007
Secondary Partner	-0.58	0.30	0.56	[0.31, 1.03]	-1.89	.061	161	172	504	0.72	.154
Secondary Actor	0.19	0.19	1.20	[0.83, 1.75]	0.98	.328	161	172	504	1.05	.782
Secondary Relationship	-0.07	0.18	0.93	[0.66, 1.33]	-0.38	.701	161	172	504	1.06	.716
**Study 3**											
Primary Partner	0.34	0.78	1.41	[0.3, 6.61]	0.44	.663	90	101	282	1.41	.642

Primary Actor	0.29	0.82	1.33	[0.26, 6.82]	0.35	.728	90	101	282	1.46	.176
Primary Relationship	0.27	0.71	1.31	[0.32, 5.37]	0.38	.705	90	101	282	1.42	.650
Secondary Partner	-0.11	0.93	0.89	[0.14, 5.71]	-0.12	.905	90	101	282	0.97	.973
Secondary Actor	0.17	0.80	1.18	[0.24, 5.77]	0.21	.835	90	101	282	1.22	.375
Secondary Relationship	0.04	0.71	1.04	[0.26, 4.23]	0.06	.954	90	101	282	1.09	.680
**Meta-Analysis**											
Primary Partner	0.96	0.17	2.62	[1.87, 3.69]	5.55	< .001	-	-	-	1.92	< .001
Primary Actor	0.07	0.16	1.08	[0.78, 1.49]	0.45	.651	-	-	-	1.05	.683
Primary Relationship	0.65	0.15	1.91	[1.44, 2.54]	4.44	< .001	-	-	-	1.56	< .001
Secondary Partner	-0.67	0.17	0.51	[0.36, 0.71]	3.96	< .001	-	-	-	0.61	< .001
Secondary Actor	0.10	0.13	1.11	[0.85, 1.44]	0.76	.449	-	-	-	1.02	.849
Secondary Relationship	-0.04	0.12	0.96	[0.76, 1.21]	0.34	.732	-	-	-	1.08	.448

Supplementary Table S11B

Multilevel Logistic Regressions and Meta-Analyses for Maximal Models Predicting Hanging Out or Corresponding

	Maximal Model									Original Model	
Study (DV) and IV	Logit	SE	OR	95% CI	Z	*p*	DF	*n*	Rows	OR	*p*
**Study 1 (Hangout / Corr.)**											
Primary Partner	0.18	0.12	1.20	[0.95, 1.51]	1.56	.122	119	130	1,442	1.15	.204
Primary Actor	0.03	0.14	1.03	[0.78, 1.36]	0.21	.833	119	130	1,442	0.86	.263
Primary Relationship	0.26	0.10	1.29	[1.06, 1.58]	2.55	.012	119	130	1,442	1.27	.014
Secondary Partner	0.26	0.15	1.30	[0.96, 1.74]	1.73	.087	119	130	1,442	1.22	.156
Secondary Actor	0.01	0.11	1.01	[0.81, 1.25]	0.05	.960	119	130	1,442	0.89	.259
Secondary Relationship	0.29	0.10	1.34	[1.10, 1.64]	2.96	.004	119	130	1,442	1.34	.002
**Study 2 (Correspond)**											
Primary Partner	0.10	0.11	1.11	[0.89, 1.38]	0.89	.374	142	153	2,403	1.06	.596
Primary Actor	-0.15	0.11	0.86	[0.69, 1.08]	-1.31	.193	142	153	2,403	0.78	.024
Primary Relationship	0.36	0.10	1.43	[1.17, 1.76]	3.45	< .001	142	153	2,403	1.49	< .001
Secondary Partner	0.18	0.12	1.19	[0.95, 1.51]	1.51	.132	142	153	2,403	1.11	.399

Secondary Actor	-0.09	0.11	0.91	[0.74, 1.13]	-0.86	.391	142	153	2,403	0.81	.050
Secondary Relationship	0.45	0.10	1.58	[1.28, 1.94]	4.37	< .001	142	153	2,403	1.64	< .001
**Study 2 (Hangout)**											
Primary Partner	0.08	0.21	1.09	[0.72, 1.64]	0.39	.694	142	153	2,397	0.99	.955
Primary Actor	-0.05	0.19	0.95	[0.66, 1.38]	-0.27	.788	142	153	2,397	0.86	.426
Primary Relationship	0.48	0.20	1.62	[1.09, 2.40]	2.40	.018	142	153	2,397	1.66	.010
Secondary Partner	0.12	0.20	1.13	[0.76, 1.68]	0.62	.536	142	153	2,397	0.99	.974
Secondary Actor	0.10	0.20	1.11	[0.75, 1.65]	0.52	.601	142	153	2,397	1.01	.941
Secondary Relationship	0.43	0.20	1.53	[1.04, 2.27]	2.15	.033	142	153	2,397	1.60	.019
**Study 3 (Interaction)**											
Primary Partner	0.43	0.18	1.54	[1.07, 2.21]	2.36	.020	103	114	2,308	1.73	.005
Primary Actor	0.09	0.20	1.09	[0.74, 1.62]	0.46	.648	103	114	2,308	1.05	.818
Primary Relationship	0.50	0.16	1.65	[1.20, 2.28]	3.12	.002	103	114	2,308	1.69	.002
Secondary Partner	0.44	0.19	1.55	[1.07, 2.25]	2.34	.021	103	114	2,308	1.69	.007
Secondary Actor	0.29	0.17	1.33	[0.95, 1.87]	1.68	.096	103	114	2,308	1.35	.094
Secondary Relationship	0.30	0.17	1.35	[0.97, 1.87]	1.79	.076	103	114	2,308	1.42	.049
**Meta-Analysis**											
Primary Partner	0.18	0.07	1.19	[1.04, 1.37]	2.52	.012	-	-	-	1.15	.032
Primary Actor	-0.05	0.07	0.95	[0.82, 1.10]	0.69	.492	-	-	-	0.84	.019
Primary Relationship	0.35	0.06	1.42	[1.26, 1.61]	5.63	< .001	-	-	-	1.43	< .001
Secondary Partner	0.23	0.08	1.26	[1.09, 1.47]	3.06	.002	-	-	-	1.20	.018
Secondary Actor	0.02	0.07	1.02	[0.90, 1.16]	0.32	.745	-	-	-	0.92	.208
Secondary Relationship	0.37	0.06	1.44	[1.28, 1.63]	5.84	< .001	-	-	-	1.47	< .001

Supplementary Table S11C

Multilevel Logistic Regressions and Meta-Analyses for Maximal Models Predicting Later Romantic Interest (Binary)

	Maximal Model									Original Model	
Study and IV	Logit	SE	OR	95% CI	Z	*p*	DF	*n*	Rows	OR	*p*
**Study 1**											

Primary Partner	1.02	0.22	2.77	[1.78, 4.31]	4.55	< .001	119	130	1,442	2.06	< .001
Primary Actor	0.45	0.22	1.56	[1.02, 2.40]	2.07	.041	119	130	1,442	1.16	.471
Primary Relationship	0.78	0.19	2.18	[1.48, 3.19]	4.02	< .001	119	130	1,442	1.69	.004
Secondary Partner	-0.19	0.22	0.82	[0.53, 1.28]	-0.87	.387	119	130	1,442	0.83	.366
Secondary Actor	-0.21	0.20	0.81	[0.55, 1.20]	-1.07	.285	119	130	1,442	0.67	.039
Secondary Relationship	0.20	0.17	1.22	[0.87, 1.70]	1.16	.250	119	130	1,442	1.33	.103
**Study 2**											
Primary Partner	1.38	0.20	3.97	[2.69, 5.85]	7.0	< .001	142	153	2,403	2.85	< .001
Primary Actor	0.52	0.19	1.68	[1.16, 2.43]	2.77	.006	142	153	2,403	1.11	.537
Primary Relationship	0.86	0.15	2.37	[1.76, 3.19]	5.76	< .001	142	153	2,403	1.78	< .001
Secondary Partner	0.37	0.19	1.45	[1.00, 2.12]	1.96	.052	142	153	2,403	1.39	.063
Secondary Actor	0.02	0.16	1.02	[0.74, 1.40]	0.12	.908	142	153	2,403	0.76	.122
Secondary Relationship	0.14	0.13	1.15	[0.89, 1.49]	1.08	.283	142	153	2,403	1.19	.217
**Meta-Analysis**											
Primary Partner	1.22	0.15	3.39	[2.54, 4.53]	8.26	< .001	-	-	-	2.45	< .001
Primary Actor	0.49	0.14	1.63	[1.23, 2.15]	3.44	< .001	-	-	-	1.13	.347
Primary Relationship	0.83	0.12	2.30	[1.82, 2.89]	7.02	< .001	-	-	-	1.75	< .001
Secondary Partner	0.13	0.14	1.14	[0.86, 1.52]	0.92	.355	-	-	-	1.12	.398
Secondary Actor	-0.07	0.12	0.93	[0.73, 1.19]	0.58	.559	-	-	-	0.72	.011
Secondary Relationship	0.16	0.10	1.18	[0.96, 1.44]	1.56	.119	-	-	-	1.24	.046

Supplementary Tables S11A-S11C. The tables show a summary of each follow up maximal model multilevel logistic regression analysis (i.e., models that included all six Primary and Secondary SRM Effects as simultaneous predictors of the outcome), as well as the meta-analyzed effect size within each grouping of variables. The Study and IV column shows which study the analysis came from and the independent variables that were included in each model (i.e., the six SRM effects). In the case of Hanging Out or Correspond (Supplementary Table S11B), this column also indicates the specific dependent variable (DV) that was analyzed in each model (for the other tables, the DV that was analyzed was the same variable indicated in the table title). Study 1 indicates the NSDS I (college sample), Study 2 indicates NSDS II (college sample), and Study 3 indicates the Anime North Study (community sample). Logit indicates the slope of the predictor, and SE indicates the standard error of prediction (in logit units). OR indicates the slope in Odds Ratio. 95% CI indicates the upper and lower 95% confidence intervals (in odds ratio units). *Z* indicates the test statistic. *n* indicates how many unique respondents were available for each analysis. DF indicates the degrees of freedom used to calculate the *p* value for

each analysis and was calculated as *n* minus one for each predictor (i.e., the six SRM independent variables) minus one for each of the four random effects (i.e., Participant ID, Partner ID, Participant ID nested within Partner ID, and Partner ID nested within Participant ID) minus one. Rows indicates how many rows of data were used in the analysis. The *p* values listed are not corrected for multiple comparisons because the primary purpose of each analysis was to combine them using meta-analysis, and we focus our interpretation on the meta-analyzed effect sizes and confidence intervals. For convenience, the OR and *p* values for the original analyses (i.e., the models in which each SRM effect was used to predict each outcome variable on its own, with no other predictors included in the model) are also reported in the table (for more information about the original analyses, see Supplementary Tables S2A-S2F and Table 1 in the main manuscript).

Supplementary Tables S12A-S12D

These tables show the sensitivity analyses in which the original multilevel continuous regression analyses were repeated with all six Primary and Secondary SRM effect included in each model as simultaneous predictors of the continuous outcome variable (i.e., maximal models). The tables also report the meta-analyzed coefficients for each grouping of variables. See below for a combined table caption. Note, because six predictors were included in each model at a time, these supplementary tables are organized by outcome variables (i.e., each table shows the results for a different grouping of outcome variables) rather than by SRM effect (as all the other results tables are).

Supplementary Table S12A

Multilevel Continuous Regressions for Maximal Models Predicting Later Interest (Continuous)

	Maximal Model								Original Model	
Study and IV	β	SE	95% CI	*t*	*p*	DF	*n*	Rows	β	*p*
**Study 3**										
Primary Partner	0.19	0.04	[0.10, 0.28]	4.34	< .001	93	104	2,282	0.17	< .001
Primary Actor	0.38	0.08	[0.22, 0.54]	4.75	< .001	93	104	2,282	0.37	< .001
Primary Relationship	0.20	0.04	[0.11, 0.28]	4.69	< .001	93	104	2,282	0.19	< .001
Secondary Partner	-0.03	0.07	[-0.17, 0.12]	-0.40	.690	93	104	2,282	-0.01	.900
Secondary Actor	0.08	0.04	[-0.01, 0.16]	1.84	.070	93	104	2,282	0.08	.080
Secondary Relationship	0.02	0.04	[-0.06, 0.10]	0.44	.662	93	104	2,282	0.03	.578

Supplementary Table S12B

Multilevel Continuous Regressions and Meta-Analyses for Maximal Models Predicting Desire to Know Better

	Maximal Model								Original Model	
Study and IV	β	SE	95% CI	*t*	*p*	DF	*n*	Rows	β	*p*
**Study 1**										
Primary Partner	0.34	0.05	[0.25, 0.43]	7.34	< .001	119	130	1,439	0.26	< .001
Primary Actor	0.20	0.06	[0.08, 0.31]	3.47	< .001	119	130	1,439	0.14	.011
Primary Relationship	0.19	0.04	[0.11, 0.27]	4.84	< .001	119	130	1,439	0.11	.007

Secondary Partner	-0.07	0.06	[-0.18, 0.05]	-1.13	.261	119	130	1,439	-0.10	.092
Secondary Actor	-0.03	0.04	[-0.12, 0.05]	-0.78	.439	119	130	1,439	-0.11	.021
Secondary Relationship	0.04	0.04	[-0.04, 0.12]	1.06	.290	119	130	1,439	0.07	.112
**Study 2**										
Primary Partner	0.26	0.04	[0.19, 0.33]	7.40	< .001	142	153	2,402	0.20	< .001
Primary Actor	0.13	0.05	[0.03, 0.23]	2.55	.012	142	153	2,402	0.07	.158
Primary Relationship	0.17	0.03	[0.11, 0.23]	5.39	< .001	142	153	2,402	0.10	.001
Secondary Partner	0.01	0.05	[-0.09, 0.12]	0.27	.791	142	153	2,402	0.01	.822
Secondary Actor	0.00	0.03	[-0.06, 0.07]	0.10	.921	142	153	2,402	-0.05	.198
Secondary Relationship	0.01	0.03	[-0.05, 0.07]	0.37	.710	142	153	2,402	0.02	.481
**Meta-Analysis**										
Primary Partner	0.29	0.03	[0.24, 0.35]	10.35	< .001	-	-	-	0.22	< .001
Primary Actor	0.16	0.04	[0.09, 0.23]	4.22	< .001	-	-	-	0.10	.005
Primary Relationship	0.18	0.02	[0.13, 0.23]	7.23	< .001	-	-	-	0.11	< .001
Secondary Partner	-0.02	0.04	[-0.10, 0.06]	0.56	.578	-	-	-	-0.04	.340
Secondary Actor	-0.01	0.03	[-0.06, 0.04]	0.38	.703	-	-	-	-0.07	.014
Secondary Relationship	0.02	0.02	[-0.02, 0.07]	0.94	.346	-	-	-	0.04	.124

Supplementary Table S12C

Multilevel Continuous Regressions and Meta-Analyses for Maximal Models Predicting Physical Attractiveness

	Maximal Model								Original Model	
Study and IV	β	SE	95% CI	*t*	*p*	DF	*n*	Rows	β	*p*
**Study 1**										
Primary Partner	0.57	0.04	[0.48, 0.65]	12.95	< .001	119	130	1,438	0.47	< .001
Primary Actor	0.31	0.05	[0.20, 0.41]	5.76	< .001	119	130	1,438	0.23	< .001
Primary Relationship	0.28	0.03	[0.22, 0.35]	8.52	< .001	119	130	1,438	0.19	< .001
Secondary Partner	0.00	0.06	[-0.12, 0.11]	-0.07	.945	119	130	1,438	-0.04	.481
Secondary Actor	-0.05	0.04	[-0.14, 0.03]	-1.25	.213	119	130	1,438	-0.19	.001
Secondary Relationship	-0.07	0.03	[-0.13, 0.00]	-1.92	.057	119	130	1,438	-0.02	.590

**Study 2**										
Primary Partner	0.57	0.04	[0.48, 0.65]	12.82	< .001	142	153	2,400	0.48	< .001
Primary Actor	0.22	0.05	[0.13, 0.31]	4.86	< .001	142	153	2,400	0.12	.004
Primary Relationship	0.25	0.03	[0.19, 0.32]	8.14	< .001	142	153	2,400	0.18	< .001
Secondary Partner	0.01	0.05	[-0.09, 0.10]	0.15	.881	142	153	2,400	0.0	.972
Secondary Actor	-0.08	0.04	[-0.16, 0.00]	-1.93	.056	142	153	2,400	-0.18	.002
Secondary Relationship	-0.01	0.03	[-0.08, 0.05]	-0.47	.639	142	153	2,400	0.01	.839
**Meta-Analysis**										
Primary Partner	0.57	0.03	[0.51, 0.63]	18.22	< .001	-	-	-	0.48	< .001
Primary Actor	0.26	0.03	[0.19, 0.32]	7.43	< .001	-	-	-	0.16	< .001
Primary Relationship	0.27	0.02	[0.22, 0.31]	11.76	< .001	-	-	-	0.18	< .001
Secondary Partner	0.00	0.04	[-0.07, 0.07]	0.07	.944	-	-	-	-0.01	.691
Secondary Actor	-0.07	0.03	[-0.13, -0.01]	2.26	.024	-	-	-	-0.19	< .001
Secondary Relationship	-0.04	0.02	[-0.08, 0.01]	1.65	.100	-	-	-	-0.01	.838

Supplementary Table S12D

Multilevel Continuous Regressions and Meta-Analyses for Maximal Models Predicting Perceived Attraction from Match

	Maximal Model								Original Model	
Study and IV	β	SE	95% CI	*t*	*p*	DF	*n*	Rows	β	*p*
**Study 1**										
Primary Partner	0.02	0.04	[-0.06, 0.10]	0.42	.677	119	130	1,439	0.01	.767
Primary Actor	0.03	0.07	[-0.11, 0.16]	0.42	.674	119	130	1,439	-0.05	.463
Primary Relationship	0.01	0.03	[-0.06, 0.08]	0.33	.745	119	130	1,439	0.02	.589
Secondary Partner	0.24	0.07	[0.10, 0.38]	3.32	.001	119	130	1,439	0.19	.006
Secondary Actor	0.03	0.04	[-0.04, 0.11]	0.86	.393	119	130	1,439	-0.01	.772
Secondary Relationship	0.14	0.03	[0.07, 0.21]	4.11	< .001	119	130	1,439	0.12	< .001
**Study 2**										
Primary Partner	0.01	0.04	[-0.06, 0.08]	0.32	.750	142	153	2,402	0.02	.593
Primary Actor	0.05	0.06	[-0.06, 0.16]	0.84	.403	142	153	2,402	-0.01	.903

Primary Relationship	0.00	0.03	[-0.06, 0.07]	0.13	.896	142	153	2,402	0.0	.961
Secondary Partner	0.29	0.06	[0.17, 0.40]	4.95	< .001	142	153	2,402	0.26	< .001
Secondary Actor	0.02	0.03	[-0.05, 0.08]	0.52	.606	142	153	2,402	-0.01	.838
Secondary Relationship	0.07	0.03	[0.01, 0.13]	2.22	.028	142	153	2,402	0.06	.080
**Study 3**										
Primary Partner	0.07	0.04	[-0.02, 0.15]	1.53	.131	93	104	2,281	0.06	.145
Primary Actor	0.30	0.07	[0.15, 0.45]	4.07	< .001	93	104	2,281	0.28	< .001
Primary Relationship	0.08	0.04	[0.00, 0.17]	2.0	.049	93	104	2,281	0.09	.042
Secondary Partner	0.08	0.07	[-0.05, 0.21]	1.17	.246	93	104	2,281	0.07	.331
Secondary Actor	0.08	0.04	[0.00, 0.16]	1.95	.054	93	104	2,281	0.08	.064
Secondary Relationship	0.07	0.04	[-0.02, 0.15]	1.61	.110	93	104	2,281	0.06	.142
**Meta-Analysis**										
Primary Partner	0.03	0.02	[-0.02, 0.07]	1.25	.213	-	-	-	0.03	.210
Primary Actor	0.11	0.04	[0.03, 0.18]	2.84	.004	-	-	-	0.05	.147
Primary Relationship	0.03	0.02	[-0.01, 0.07]	1.27	.205	-	-	-	0.03	.181
Secondary Partner	0.21	0.04	[0.14, 0.28]	5.58	< .001	-	-	-	0.19	< .001
Secondary Actor	0.04	0.02	[0.00, 0.08]	1.84	.065	-	-	-	0.01	.511
Secondary Relationship	0.10	0.02	[0.06, 0.14]	4.64	< .001	-	-	-	0.08	< .001

Supplementary Tables S12A-S12D. The tables show a summary of each follow up maximal model multilevel continuous regression analysis (i.e., models that included all six Primary and Secondary SRM Effects as simultaneous predictors of the outcome), as well as the meta-analyzed effect size within each grouping of variables. The Study and IV column shows which study the analysis came from and the independent variables that were included in each model (i.e., the six SRM effects). Study 1 indicates the NSDS I (college sample), Study 2 indicates NSDS II (college sample), and Study 3 indicates the Anime North Study (community sample). The dependent variable in each model is indicated in the name of the table title. β indicates the slope of the predictor (the standardized beta weight), and SE indicates the standard error of prediction. 95% CI indicates the upper and lower 95% confidence intervals (on the beta scale). *t* indicates the test statistic. *n* indicates how many unique respondents were available for each analysis. DF indicates the degrees of freedom used to calculate the *p* value for each analysis and was calculated as *n* minus one for each predictor (i.e., the six SRM independent variables) minus one for each of the four random effects (i.e., Participant ID, Partner ID, Participant ID nested within Partner ID, and Partner ID nested within Participant ID) minus one. Rows indicates how many rows of data were used in the analysis. The *p* values listed are not corrected for multiple comparisons because the primary purpose of each analysis was to combine them

using meta-analysis, and we focus our interpretation on the meta-analyzed effect sizes and confidence intervals. For convenience, the β and *p* values for the original analyses (i.e., the models in which each SRM effect was used to predict each outcome variable on its own, with no other predictors included in the model) are also reported in the table (for more information about the original analyses, see Supplementary Tables S3A-S3F and Tables 2-3 in the main manuscript).

Supplementary Tables S13A-S13F

These tables show the sensitivity analyses in which each original multilevel logistic regression was repeated with separately for the SRM effects in initial romantic liking and for the SRM effects in initial sexual desire. The tables also report the meta-analyzed coefficients for each grouping of variables. See below for a combined table caption.

Supplementary Table S13A

Multilevel Logistic Regressions and Meta-Analyses for Primary Partner Effects in Initial Romantic vs. Initial Sexual Desire

		Model with Only Initial Romantic Liking or Initial Sexual Desire									Original Model	
Grouping and Study	Model	Logit	SE	OR	95% CI	*Z*	*p*	DF	*n*	Rows	OR	*p*
**Contact Initiation**												
Study 1	DV: Contact Initiation											
	Initial Romantic Liking	0.33	0.18	1.39	[0.97, 1.99]	1.82	.070	132	138	412	1.98	< .001
	Initial Sexual Desire	0.81	0.19	2.24	[1.54, 3.25]	4.29	< .001	132	138	412	1.98	< .001
Study 2	DV: Contact Initiation											
	Initial Romantic Liking	0.52	0.19	1.68	[1.16, 2.42]	2.77	.006	166	172	504	1.90	< .001
	Initial Sexual Desire	0.62	0.18	1.85	[1.29, 2.66]	3.34	.001	166	172	504	1.90	< .001
Study 3	DV: Contact Initiation											
	Initial Romantic Liking	0.04	0.79	1.04	[0.22, 5.06]	0.05	.958	91	97	245	1.41	.642
	Initial Sexual Desire†	0.48	0.29	1.61	[0.90, 2.87]	1.64	.105	86	89	218	1.41	.642
Meta	DV: Contact Initiation											
	Initial Romantic Liking	0.41	0.13	1.51	[1.17, 1.94]	3.20	.001	-	-	-	1.92	< .001
	Initial Sexual Desire	0.67	0.12	1.95	[1.54, 2.47]	5.59	< .001	-	-	-	1.92	< .001
**Hangout or Correspond**												
Study 1	DV: Hangout or Correspond											
	Initial Romantic Liking	0.07	0.11	1.07	[0.87, 1.32]	0.67	.504	124	130	1,442	1.15	.204
	Initial Sexual Desire	0.15	0.11	1.16	[0.94, 1.43]	1.39	.167	124	130	1,442	1.15	.204
Study 2	DV: Correspond											

	Initial Romantic Liking	-0.03	0.11	0.97	[0.78, 1.21]	-0.26	.798	147	153	2,403	1.06	.596
	Initial Sexual Desire	0.07	0.11	1.08	[0.86, 1.34]	0.66	.510	147	153	2,403	1.06	.596
Study 2	DV: Hangout											
	Initial Romantic Liking	-0.02	0.20	0.98	[0.66, 1.45]	-0.09	.927	147	153	2,397	0.99	.955
	Initial Sexual Desire	-0.09	0.20	0.92	[0.62, 1.36]	-0.44	.661	147	153	2,397	0.99	.955
Study 3	DV: Interaction with Match											
	Initial Romantic Liking	0.59	0.20	1.81	[1.21, 2.71]	2.93	.004	103	109	2,090	1.73	.005
	Initial Sexual Desire	0.52	0.21	1.68	[1.12, 2.54]	2.53	.013	92	98	1,684	1.73	.005
Meta	DV: Hangout or Correspond											
	Initial Romantic Liking	0.08	0.07	1.09	[0.95, 1.24]	1.22	.223	-	-	-	1.15	.032
	Initial Sexual Desire	0.13	0.07	1.14	[1.00, 1.31]	1.97	.049	-	-	-	1.15	.032
**Later Romantic Interest (Binary)**												
Study 1	DV: Later Romantic Interest											
	Initial Romantic Liking	0.34	0.20	1.41	[0.95, 2.10]	1.72	.087	124	130	1,442	2.06	< .001
	Initial Sexual Desire	0.86	0.19	2.37	[1.62, 3.47]	4.51	< .001	124	130	1,442	2.06	< .001
Study 2	DV: Later Romantic Interest											
	Initial Romantic Liking	0.73	0.18	2.08	[1.45, 2.99]	3.99	< .001	147	153	2,403	2.85	< .001
	Initial Sexual Desire	1.16	0.17	3.19	[2.26, 4.48]	6.70	< .001	147	153	2,403	2.85	< .001
Meta	DV: Later Romantic Interest											
	Initial Romantic Liking	0.56	0.14	1.74	[1.34, 2.27]	4.10	< .001	-	-	-	2.45	< .001
	Initial Sexual Desire	1.03	0.13	2.79	[2.17, 3.59]	7.99	< .001	-	-	-	2.45	< .001

Supplementary Table S13B

Multilevel Logistic Regressions and Meta-Analyses for Primary Actor Effects in Initial Romantic vs. Initial Sexual Desire

		Model with Only Initial Romantic Liking or Initial Sexual Desire									Original Model	
Grouping and Study	Model	Logit	SE	OR	95% CI	*Z*	*p*	DF	*n*	Rows	OR	*p*
**Contact Initiation**												

Study 1	DV: Contact Initiation												
		Initial Romantic Liking	-0.05	0.18	0.95	[0.67, 1.35]	-0.30	.768	132	138	412	1.05	.790
		Initial Sexual Desire	0.17	0.18	1.19	[0.83, 1.71]	0.95	.345	132	138	412	1.05	.790
Study 2	DV: Contact Initiation												
		Initial Romantic Liking	-0.30	0.24	0.74	[0.46, 1.21]	-1.21	.229	166	172	504	0.83	.443
		Initial Sexual Desire	-0.14	0.23	0.87	[0.55, 1.36]	-0.63	.528	166	172	504	0.83	.443
Study 3	DV: Contact Initiation												
		Initial Romantic Liking	0.46	0.28	1.59	[0.90, 2.79]	1.62	.109	91	97	245	1.46	.176
		Initial Sexual Desire†	0.32	0.39	1.37	[0.64, 2.96]	0.82	.415	86	89	218	1.46	.176
Meta	DV: Contact Initiation												
		Initial Romantic Liking	-0.01	0.13	0.99	[0.77, 1.27]	0.12	.907	-	-	-	1.05	.683
		Initial Sexual Desire	0.08	0.13	1.08	[0.83, 1.41]	0.61	.543	-	-	-	1.05	.683
**Hangout or Correspond**													
Study 1	DV: Hangout or Correspond												
		Initial Romantic Liking	-0.10	0.13	0.91	[0.71, 1.17]	-0.77	.443	124	130	1,442	0.86	.263
		Initial Sexual Desire	-0.13	0.13	0.88	[0.68, 1.14]	-0.99	.324	124	130	1,442	0.86	.263
Study 2	DV: Correspond												
		Initial Romantic Liking	-0.22	0.11	0.80	[0.65, 1.00]	-2.01	.046	147	153	2,403	0.78	.024
		Initial Sexual Desire	-0.15	0.11	0.86	[0.69, 1.07]	-1.39	.167	147	153	2,403	0.78	.024
Study 2	DV: Hangout												
		Initial Romantic Liking	-0.04	0.19	0.96	[0.67, 1.38]	-0.22	.826	147	153	2,397	0.86	.426
		Initial Sexual Desire	-0.17	0.18	0.85	[0.59, 1.22]	-0.91	.364	147	153	2,397	0.86	.426
Study 3	DV: Interaction with Match												
		Initial Romantic Liking	0.07	0.22	1.07	[0.70, 1.65]	0.32	.746	103	109	2,090	1.05	.818
		Initial Sexual Desire	-0.15	0.22	0.86	[0.56, 1.33]	-0.68	.497	92	98	1,684	1.05	.818
Meta	DV: Hangout or Correspond												
		Initial Romantic Liking	-0.12	0.07	0.88	[0.77, 1.02]	1.73	.084	-	-	-	0.84	.019
		Initial Sexual Desire	-0.15	0.07	0.86	[0.75, 0.99]	2.04	.041	-	-	-	0.84	.019
**Later Romantic Interest (Binary)**													
Study 1	DV: Later Romantic Interest												

	Initial Romantic Liking	-0.05	0.19	0.95	[0.66, 1.38]	-0.26	.794	124	130	1,442	1.16	.471
	Initial Sexual Desire	0.33	0.20	1.39	[0.94, 2.06]	1.67	.098	124	130	1,442	1.16	.471
Study 2	DV: Later Romantic Interest											
	Initial Romantic Liking	-0.01	0.17	0.99	[0.71, 1.39]	-0.05	.964	147	153	2,403	1.11	.537
	Initial Sexual Desire	0.28	0.16	1.33	[0.96, 1.84]	1.73	.086	147	153	2,403	1.11	.537
Meta	DV: Later Romantic Interest											
	Initial Romantic Liking	-0.03	0.13	0.97	[0.76, 1.25]	0.21	.834	-	-	-	1.13	.347
	Initial Sexual Desire	0.30	0.13	1.35	[1.06, 1.73]	2.40	.017	-	-	-	1.13	.347

Supplementary Table S13C

Multilevel Logistic Regressions and Meta-Analyses for Primary Relationship Effects in Initial Romantic vs. Initial Sexual Desire

		Model with Only Initial Romantic Liking or Initial Sexual Desire									Original Model	
Grouping and Study	Model	Logit	SE	OR	95% CI	*Z*	*p*	DF	*n*	Rows	OR	*p*
**Contact Initiation**												
Study 1	DV: Contact Initiation											
	Initial Romantic Liking	0.20	0.15	1.23	[0.90, 1.66]	1.32	.189	132	138	412	1.44	.025
	Initial Sexual Desire	0.33	0.15	1.39	[1.03, 1.89]	2.17	.032	132	138	412	1.44	.025
Study 2	DV: Contact Initiation											
	Initial Romantic Liking	0.59	0.21	1.80	[1.18, 2.74]	2.77	.006	166	172	504	1.83	.007
	Initial Sexual Desire	0.33	0.17	1.39	[1.00, 1.95]	1.96	.051	166	172	504	1.83	.007
Study 3	DV: Contact Initiation											
	Initial Romantic Liking	0.22	0.81	1.25	[0.25, 6.27]	0.27	.786	91	97	245	1.42	.650
	Initial Sexual Desire†	0.15	0.28	1.16	[0.66, 2.04]	0.54	.594	86	89	218	1.42	.650
Meta	DV: Contact Initiation											
	Initial Romantic Liking	0.33	0.12	1.40	[1.10, 1.78]	2.70	.007	-	-	-	1.56	< .001
	Initial Sexual Desire	0.31	0.11	1.36	[1.11, 1.67]	2.91	.004	-	-	-	1.56	< .001
**Hangout or Correspond**												

Study 1	DV: Hangout or Correspond											
	Initial Romantic Liking	0.13	0.09	1.13	[0.94, 1.36]	1.35	.179	124	130	1,442	1.27	.014
	Initial Sexual Desire	0.29	0.09	1.34	[1.11, 1.61]	3.11	.002	124	130	1,442	1.27	.014
Study 2	DV: Correspond											
	Initial Romantic Liking	0.39	0.10	1.47	[1.21, 1.79]	3.90	< .001	147	153	2,403	1.49	< .001
	Initial Sexual Desire	0.20	0.10	1.22	[1.00, 1.48]	1.98	.049	147	153	2,403	1.49	< .001
Study 2	DV: Hangout											
	Initial Romantic Liking	0.59	0.19	1.81	[1.24, 2.64]	3.11	.002	147	153	2,397	1.66	.010
	Initial Sexual Desire	0.20	0.20	1.23	[0.83, 1.81]	1.05	.297	147	153	2,397	1.66	.010
Study 3	DV: Interaction with Match											
	Initial Romantic Liking	0.43	0.19	1.54	[1.07, 2.22]	2.32	.022	103	109	2,090	1.69	.002
	Initial Sexual Desire	0.39	0.17	1.47	[1.04, 2.08]	2.22	.029	92	98	1,684	1.69	.002
Meta	DV: Hangout or Correspond											
	Initial Romantic Liking	0.30	0.06	1.35	[1.20, 1.52]	4.99	< .001	-	-	-	1.43	< .001
	Initial Sexual Desire	0.26	0.06	1.30	[1.15, 1.46]	4.30	< .001	-	-	-	1.43	< .001
**Later Romantic Interest (Binary)**												
Study 1	DV: Later Romantic Interest											
	Initial Romantic Liking	0.13	0.17	1.13	[0.81, 1.59]	0.73	.467	124	130	1,442	1.69	.004
	Initial Sexual Desire	0.80	0.18	2.23	[1.56, 3.18]	4.46	< .001	124	130	1,442	1.69	.004
Study 2	DV: Later Romantic Interest											
	Initial Romantic Liking	0.29	0.14	1.33	[1.01, 1.76]	2.06	.041	147	153	2,403	1.78	< .001
	Initial Sexual Desire	0.65	0.13	1.92	[1.48, 2.49]	4.95	< .001	147	153	2,403	1.78	< .001
Meta	DV: Later Romantic Interest											
	Initial Romantic Liking	0.22	0.11	1.25	[1.01, 1.55]	2.06	.039	-	-	-	1.75	< .001
	Initial Sexual Desire	0.70	0.11	2.02	[1.64, 2.49]	6.63	< .001	-	-	-	1.75	< .001

Supplementary Table S13D

Multilevel Logistic Regressions and Meta-Analyses for Secondary Partner Effects in Initial Romantic vs. Initial Sexual Desire

	Model with Only Initial Romantic Liking or Initial Sexual Desire	Original Model

Grouping and Study	Model	Logit	SE	OR	95% CI	*Z*	*p*	DF	*n*	Rows	OR	*p*
**Contact Initiation**												
Study 1	DV: Contact Initiation											
	Initial Romantic Liking	-0.37	0.18	0.69	[0.49, 0.98]	-2.09	.039	132	138	412	0.54	< .001
	Initial Sexual Desire	-0.71	0.18	0.49	[0.34, 0.70]	-3.90	< .001	132	138	412	0.54	< .001
Study 2	DV: Contact Initiation											
	Initial Romantic Liking	-0.31	0.23	0.73	[0.47, 1.14]	-1.38	.170	166	172	504	0.72	.154
	Initial Sexual Desire	-0.36	0.23	0.70	[0.44, 1.10]	-1.55	.123	166	172	504	0.72	.154
Study 3	DV: Contact Initiation											
	Initial Romantic Liking	0.26	0.30	1.30	[0.71, 2.38]	0.86	.389	124	130	245	0.97	.973
	Initial Sexual Desire†	0.36	0.23	1.43	[0.91, 2.26]	1.56	.122	116	119	218	0.97	.973
Meta	DV: Contact Initiation											
	Initial Romantic Liking	-0.24	0.13	0.79	[0.61, 1.01]	1.91	.056	-	-	-	0.61	< .001
	Initial Sexual Desire	-0.32	0.12	0.73	[0.57, 0.93]	2.60	.009	-	-	-	0.61	< .001
**Hangout or Correspond**												
Study 1	DV: Hangout or Correspond											
	Initial Romantic Liking	0.11	0.14	1.11	[0.85, 1.46]	0.77	.443	131	137	1,442	1.22	.156
	Initial Sexual Desire	0.23	0.14	1.26	[0.96, 1.67]	1.66	.099	131	137	1,442	1.22	.156
Study 2	DV: Correspond											
	Initial Romantic Liking	0.02	0.12	1.02	[0.81, 1.28]	0.15	.883	158	164	2,403	1.11	.399
	Initial Sexual Desire	0.11	0.12	1.12	[0.88, 1.41]	0.93	.355	158	164	2,403	1.11	.399
Study 2	DV: Hangout											
	Initial Romantic Liking	-0.02	0.20	0.98	[0.66, 1.46]	-0.08	.934	158	164	2,397	0.99	.974
	Initial Sexual Desire	-0.06	0.20	0.94	[0.64, 1.40]	-0.29	.770	158	164	2,397	0.99	.974
Study 3	DV: Interaction with Match											
	Initial Romantic Liking	0.58	0.20	1.79	[1.19, 2.68]	2.83	.005	133	139	2,090	1.69	.007
	Initial Sexual Desire	0.45	0.19	1.57	[1.07, 2.31]	2.33	.021	119	125	1,684	1.69	.007
Meta	DV: Hangout or Correspond											
	Initial Romantic Liking	0.12	0.08	1.12	[0.97, 1.30]	1.53	.125	-	-	-	1.20	.018

	Initial Sexual Desire	0.17	0.08	1.19	[1.03, 1.38]	2.29	.022	-	-	-	1.20	.018
**Later Romantic Interest (Binary)**												
Study 1	DV: Later Romantic Interest											
	Initial Romantic Liking	-0.20	0.20	0.82	[0.55, 1.22]	-0.99	.323	131	137	1,442	0.83	.366
	Initial Sexual Desire	-0.16	0.21	0.85	[0.56, 1.29]	-0.76	.448	131	137	1,442	0.83	.366
Study 2	DV: Later Romantic Interest											
	Initial Romantic Liking	0.30	0.17	1.35	[0.96, 1.9]	1.74	.084	158	164	2,403	1.39	.063
	Initial Sexual Desire	0.35	0.18	1.42	[1.00, 2.01]	1.96	.052	158	164	2,403	1.39	.063
Meta	DV: Later Romantic Interest											
	Initial Romantic Liking	0.09	0.13	1.09	[0.85, 1.42]	0.69	.493	-	-	-	1.12	.398
	Initial Sexual Desire	0.14	0.14	1.15	[0.88, 1.50]	1.01	.311	-	-	-	1.12	.398

Supplementary Table S13E

Multilevel Logistic Regressions and Meta-Analyses for Secondary Actor Effects in Initial Romantic vs. Initial Sexual Desire

		Model with Only Initial Romantic Liking or Initial Sexual Desire									Original Model	
Grouping and Study	Model	Logit	SE	OR	95% CI	*Z*	*p*	DF	*n*	Rows	OR	*p*
**Contact Initiation**												
Study 1	DV: Contact Initiation											
	Initial Romantic Liking	0.07	0.17	1.07	[0.76, 1.51]	0.39	.695	132	138	412	0.89	.506
	Initial Sexual Desire	-0.28	0.17	0.75	[0.54, 1.06]	-1.65	.101	132	138	412	0.89	.506
Study 2	DV: Contact Initiation											
	Initial Romantic Liking	0.23	0.17	1.25	[0.90, 1.74]	1.35	.180	166	172	504	1.05	.782
	Initial Sexual Desire	-0.09	0.17	0.91	[0.65, 1.28]	-0.54	.593	166	172	504	1.05	.782
Study 3	DV: Contact Initiation											
	Initial Romantic Liking	-0.02	0.94	0.98	[0.15, 6.36]	-0.02	.986	124	130	245	1.22	.375
	Initial Sexual Desire†	-0.02	0.24	0.98	[0.60, 1.58]	-0.10	.923	116	119	218	1.22	.375
Meta	DV: Contact Initiation											

	Initial Romantic Liking	0.15	0.12	1.16	[0.92, 1.47]	1.23	.218	-	-	-	1.02	.849
	Initial Sexual Desire	-0.16	0.11	0.86	[0.69, 1.06]	1.43	.153	-	-	-	1.02	.849
**Hangout or Correspond**												
Study 1	DV: Hangout or Correspond											
	Initial Romantic Liking	-0.09	0.11	0.92	[0.74, 1.13]	-0.83	.407	131	137	1,442	0.89	.259
	Initial Sexual Desire	-0.10	0.10	0.91	[0.74, 1.12]	-0.92	.361	131	137	1,442	0.89	.259
Study 2	DV: Correspond											
	Initial Romantic Liking	-0.19	0.11	0.83	[0.67, 1.02]	-1.78	.077	158	164	2,403	0.81	.050
	Initial Sexual Desire	-0.18	0.11	0.83	[0.67, 1.04]	-1.64	.103	158	164	2,403	0.81	.050
Study 2	DV: Hangout											
	Initial Romantic Liking	-0.05	0.19	0.95	[0.65, 1.39]	-0.27	.788	158	164	2,397	1.01	.941
	Initial Sexual Desire	0.03	0.20	1.03	[0.69, 1.54]	0.13	.895	158	164	2,397	1.01	.941
Study 3	DV: Interaction with Match											
	Initial Romantic Liking	0.34	0.19	1.41	[0.97, 2.04]	1.81	.072	133	139	2,090	1.35	.094
	Initial Sexual Desire	0.08	0.19	1.09	[0.74, 1.60]	0.44	.661	119	125	1,684	1.35	.094
Meta	DV: Hangout or Correspond											
	Initial Romantic Liking	-0.07	0.07	0.93	[0.82, 1.06]	1.06	.288	-	-	-	0.92	.208
	Initial Sexual Desire	-0.09	0.07	0.91	[0.80, 1.04]	1.38	.168	-	-	-	0.92	.208
**Later Romantic Interest (Binary)**												
Study 1	DV: Later Romantic Interest											
	Initial Romantic Liking	-0.38	0.20	0.68	[0.46, 1.01]	-1.94	.055	131	137	1,442	0.67	.039
	Initial Sexual Desire	-0.46	0.20	0.63	[0.43, 0.93]	-2.36	.020	131	137	1,442	0.67	.039
Study 2	DV: Later Romantic Interest											
	Initial Romantic Liking	-0.18	0.18	0.84	[0.59, 1.18]	-1.01	.313	158	164	2,403	0.76	.122
	Initial Sexual Desire	-0.16	0.18	0.85	[0.60, 1.22]	-0.87	.383	158	164	2,403	0.76	.122
Meta	DV: Later Romantic Interest											
	Initial Romantic Liking	-0.27	0.13	0.76	[0.59, 0.99]	2.04	.041	-	-	-	0.72	.011
	Initial Sexual Desire	-0.30	0.13	0.74	[0.57, 0.96]	2.24	.025	-	-	-	0.72	.011

Supplementary Table S13F

Multilevel Logistic Regressions and Meta-Analyses for Secondary Relationship Effects in Initial Romantic vs. Initial Sexual Desire

		Model with Only Initial Romantic Liking or Initial Sexual Desire									Original Model	
Grouping and Study	Model	Logit	SE	OR	95% CI	*Z*	*p*	DF	*n*	Rows	OR	*p*
**Contact Initiation**												
Study 1	DV: Contact Initiation											
	Initial Romantic Liking	0.02	0.15	1.02	[0.76, 1.36]	0.11	.913	132	138	412	1.08	.592
	Initial Sexual Desire	0.04	0.15	1.04	[0.78, 1.39]	0.26	.793	132	138	412	1.08	.592
Study 2	DV: Contact Initiation											
	Initial Romantic Liking	-0.03	0.16	0.97	[0.71, 1.33]	-0.17	.863	166	172	504	1.06	.716
	Initial Sexual Desire	0.11	0.15	1.12	[0.82, 1.52]	0.72	.474	166	172	504	1.06	.716
Study 3	DV: Contact Initiation											
	Initial Romantic Liking	0.25	0.77	1.29	[0.28, 5.90]	0.33	.742	124	130	245	1.09	.680
	Initial Sexual Desire†	0.03	0.20	1.03	[0.69, 1.54]	0.17	.866	116	119	218	1.09	.680
Meta	DV: Contact Initiation											
	Initial Romantic Liking	0.0	0.11	1.0	[0.81, 1.23]	0.01	.993	-	-	-	1.08	.448
	Initial Sexual Desire	0.06	0.09	1.07	[0.89, 1.28]	0.68	.494	-	-	-	1.08	.448
**Hangout or Correspond**												
Study 1	DV: Hangout or Correspond											
	Initial Romantic Liking	0.12	0.09	1.13	[0.94, 1.36]	1.29	.199	131	137	1,442	1.34	.002
	Initial Sexual Desire	0.35	0.09	1.42	[1.19, 1.69]	3.90	< .001	131	137	1,442	1.34	.002
Study 2	DV: Correspond											
	Initial Romantic Liking	0.46	0.10	1.58	[1.29, 1.93]	4.51	< .001	158	164	2,403	1.64	< .001
	Initial Sexual Desire	0.22	0.10	1.25	[1.02, 1.53]	2.22	.028	158	164	2,403	1.64	< .001
Study 2	DV: Hangout											
	Initial Romantic Liking	0.53	0.19	1.70	[1.17, 2.47]	2.78	.006	158	164	2,397	1.60	.019
	Initial Sexual Desire	0.23	0.20	1.25	[0.84, 1.86]	1.13	.262	158	164	2,397	1.60	.019
Study 3	DV: Interaction with Match											

		Initial Romantic Liking	0.25	0.19	1.28	[0.88, 1.87]	1.32	.189	133	139	2,090	1.42	.049
		Initial Sexual Desire	0.26	0.19	1.30	[0.90, 1.89]	1.40	.164	119	125	1,684	1.42	.049
Meta	DV: Hangout or Correspond												
		Initial Romantic Liking	0.30	0.06	1.35	[1.20, 1.52]	4.89	< .001	-	-	-	1.47	< .001
		Initial Sexual Desire	0.29	0.06	1.33	[1.18, 1.50]	4.73	< .001	-	-	-	1.47	< .001
**Later Romantic Interest (Binary)**													
Study 1	DV: Later Romantic Interest												
		Initial Romantic Liking	0.15	0.17	1.17	[0.84, 1.62]	0.92	.358	131	137	1,442	1.33	.103
		Initial Sexual Desire	0.28	0.17	1.32	[0.94, 1.85]	1.61	.109	131	137	1,442	1.33	.103
Study 2	DV: Later Romantic Interest												
		Initial Romantic Liking	-0.01	0.14	0.99	[0.75, 1.29]	-0.10	.919	158	164	2,403	1.19	.217
		Initial Sexual Desire	0.30	0.14	1.35	[1.03, 1.76]	2.20	.029	158	164	2,403	1.19	.217
Meta	DV: Later Romantic Interest												
		Initial Romantic Liking	0.05	0.11	1.06	[0.86, 1.30]	0.51	.610	-	-	-	1.24	.046
		Initial Sexual Desire	0.29	0.11	1.33	[1.08, 1.64]	2.73	.006	-	-	-	1.24	.046

Supplementary Tables S13A-S13F. The tables show a summary of the follow up multilevel logistic regression analyses that considered the Primary and Secondary SRM effects in initial romantic liking and initial sexual desire as separate predictors of the dichotomous outcomes in the study, as well as the meta-analyzed effect size within each grouping of variables. The Grouping and Study column shows the which variables were grouped together when meta-analyzed (in bold) and indicate which study the coefficients come from. Study 1 indicates the NSDS I (college sample), Study 2 indicates NSDS II (college sample), and Study 3 indicates the Anime North Study (community sample). The IV in each analysis is indicated in the title of each table, and the dependent variable in each analysis is indicated in the Model column. The indented rows underneath each DV in the Model column indicate whether the model is for initial romantic liking or initial sexual desire; only one variable was included as a predictor at a time. Logit indicates the slope of the predictor, and SE indicates the standard error of prediction (in logit units). OR indicates the slope in Odds Ratio. 95% CI indicates the upper and lower 95% confidence intervals (in odds ratio units). *Z* indicates the test statistic. *n* indicates how many unique respondents were available for each analysis. DF indicates the degrees of freedom used to calculate the *p* value for each analysis and was calculated as *n* minus one for each predictor (i.e., the one SRM independent variable) minus one for each of the four random effects (i.e., Participant ID, Partner ID, Participant ID nested within Partner ID, and Partner ID nested within Participant ID) minus one (see footnotes below for a few exceptions). Rows indicates how many rows of data were used in the analysis (note, due some participants failing to report on either initial romantic liking or initial sexual desire, the number of rows and n in each model is

not always the same for initial romantic liking and initial sexual desire). The *p* values listed are not corrected for multiple comparisons because the primary purpose of each analysis was to combine them using meta-analysis, and we focus our interpretation on the meta-analyzed effect sizes and confidence intervals. For convenience, the OR and *p* values for the original analysis (i.e., the models that used the average of initial romantic and initial sexual desire, as well as the average of romantic and sexual desire for the relevant outcome variables) are also reported in the table (for more information about the original analyses, see Supplementary Tables S2A-S2F and Table 1 in the main manuscript).

† For the Study 3 Contact Initiation analyses involving the six SRM effects in Initial Sexual Desire, only one random effect was included in the model (Participant ID for the Primary SRM effects, Partner ID for the Secondary SRM effects). This was done because when the full suite of random effects were included in these models, it produced improbably large OR, SE, Z, and 95% CI ranges.

Supplementary Tables S14A-S14F

These tables show the sensitivity analyses in which each original multilevel continuous regression was repeated with random slopes (within participant ID and partner ID) included in the model. The tables also report the meta-analyzed coefficients for each grouping of variables. See below for a combined table caption.

Supplementary Table S14A

Multilevel Continuous Regressions and Meta-Analyses for Primary Partner Effects in Initial Romantic vs. Initial Sexual Desire

		Model with Only Initial Romantic Liking or Initial Sexual Desire								Original Model	
Grouping and Study	Model	β	SE	95% CI	*t*	*p*	DF	*n*	Rows	β	*p*
**Later Romantic Interest (Continuous)**											
Study 3	DV: Later Romantic Interest										
	Initial Romantic Liking	0.10	0.05	[0.00, 0.20]	2.02	.047	93	99	2,062	0.17	< .001
	Initial Sexual Desire	0.15	0.06	[0.04, 0.26]	2.65	.010	85	91	1,661	0.17	< .001
**Later Sexual Interest (Continuous)**											
Study 3	DV: Later Sexual Interest										
	Initial Romantic Liking	0.13	0.05	[0.03, 0.23]	2.70	.008	89	95	1,878	0.17	< .001
	Initial Sexual Desire	0.18	0.05	[0.08, 0.29]	3.45	< .001	82	88	1,506	0.17	< .001
**Desire to Know Better**											
Study 1	DV: Desire to Know Better										
	Initial Romantic Liking	0.22	0.04	[0.13, 0.30]	4.84	< .001	124	130	1,439	0.26	< .001
	Initial Sexual Desire	0.26	0.04	[0.18, 0.35]	5.98	< .001	124	130	1,439	0.26	< .001
Study 2	DV: Desire to Know Better										
	Initial Romantic Liking	0.17	0.04	[0.10, 0.24]	4.72	< .001	147	153	2,402	0.20	< .001
	Initial Sexual Desire	0.19	0.03	[0.12, 0.26]	5.55	< .001	147	153	2,402	0.20	< .001
Meta	DV: Know Better										
	Initial Romantic Liking	0.19	0.03	[0.13, 0.24]	6.71	< .001	-	-	-	0.22	< .001
	Initial Sexual Desire	0.22	0.03	[0.17, 0.27]	8.06	< .001	-	-	-	0.22	< .001
**Physical Attractiveness**											

Study 1	DV: Physically Attractive Rating										
	Initial Romantic Liking	0.30	0.05	[0.20, 0.41]	5.68	< .001	124	130	1,438	0.47	< .001
	Initial Sexual Desire	0.54	0.04	[0.47, 0.61]	14.80	< .001	124	130	1,438	0.47	< .001
Study 2	DV: Physically Attractive Rating										
	Initial Romantic Liking	0.35	0.05	[0.24, 0.46]	6.43	< .001	147	153	2,400	0.48	< .001
	Initial Sexual Desire	0.54	0.04	[0.46, 0.62]	13.57	< .001	147	153	2,400	0.48	< .001
Meta	DV: Physical Attractiveness										
	Initial Romantic Liking	0.33	0.04	[0.25, 0.40]	8.56	< .001	-	-	-	0.48	< .001
	Initial Sexual Desire	0.54	0.03	[0.49, 0.59]	20.08	< .001	-	-	-	0.48	< .001
**Perceived Romantic Attraction from Match**											
Study 1	DV: Perceived Romantic Attraction										
	Initial Romantic Liking	-0.03	0.04	[-0.11, 0.05]	-0.67	.506	124	130	1,439	0.01	.767
	Initial Sexual Desire	0.0	0.04	[-0.08, 0.09]	0.11	.913	124	130	1,439	0.01	.767
Study 2	DV: Perceived Romantic Attraction										
	Initial Romantic Liking	-0.02	0.03	[-0.09, 0.05]	-0.60	.549	147	153	2,401	0.02	.593
	Initial Sexual Desire	0.02	0.03	[-0.05, 0.09]	0.58	.564	147	153	2,401	0.02	.593
Study 3	DV: Perceived Romantic Attraction										
	Initial Romantic Liking	0.05	0.05	[-0.05, 0.15]	1.03	.307	93	99	2,063	0.06	.145
	Initial Sexual Desire	0.03	0.06	[-0.09, 0.14]	0.47	.643	85	91	1,663	0.06	.145
Meta	DV: Perceived Romantic Attraction										
	Initial Romantic Liking	-0.01	0.02	[-0.05, 0.04]	0.31	.760	-	-	-	0.03	.210
	Initial Sexual Desire	0.02	0.02	[-0.03, 0.06]	0.66	.506	-	-	-	0.03	.210
**Perceived Sexual Attraction from Match**											
Study 1	DV: Perceived Sexual Attraction										
	Initial Romantic Liking	0.02	0.03	[-0.05, 0.08]	0.47	.639	124	130	1,439	0.01	.767
	Initial Sexual Desire	0.04	0.03	[-0.03, 0.11]	1.22	.224	124	130	1,439	0.01	.767
Study 2	DV: Perceived Sexual Attraction										
	Initial Romantic Liking	0.01	0.03	[-0.05, 0.07]	0.34	.733	147	153	2,402	0.02	.593
	Initial Sexual Desire	0.05	0.03	[-0.01, 0.11]	1.66	.099	147	153	2,402	0.02	.593
Study 3	DV: Perceived Sexual Attraction										
	Initial Romantic Liking	0.07	0.05	[-0.02, 0.17]	1.58	.118	89	95	1,873	0.06	.145
	Initial Sexual Desire	0.05	0.06	[-0.06, 0.16]	0.88	.384	82	88	1,501	0.06	.145

Meta	DV: Perceived Sexual Attraction										
	Initial Romantic Liking	0.03	0.02	[-0.02, 0.07]	1.21	.227	-	-	-	0.03	.210
	Initial Sexual Desire	0.05	0.02	[0.01, 0.09]	2.23	.026	-	-	-	0.03	.210

Supplementary Table S14B

Multilevel Continuous Regressions and Meta-Analyses for Primary Actor Effects in Initial Romantic vs. Initial Sexual Desire

		Model with Only Initial Romantic Liking or Initial Sexual Desire								Original Model	
Grouping and Study	Model	β	SE	95% CI	*t*	*p*	DF	*n*	Rows	β	*p*
**Later Romantic Interest (Continuous)**											
Study 3	DV: Later Romantic Interest										
	Initial Romantic Liking	0.34	0.08	[0.19, 0.49]	4.53	< .001	93	99	2,062	0.37	< .001
	Initial Sexual Desire	0.29	0.09	[0.11, 0.46]	3.27	.002	85	91	1,661	0.37	< .001
**Later Sexual Interest (Continuous)**											
Study 3	DV: Later Sexual Interest										
	Initial Romantic Liking	0.39	0.08	[0.22, 0.55]	4.68	< .001	89	95	1,878	0.37	< .001
	Initial Sexual Desire	0.35	0.09	[0.16, 0.53]	3.71	< .001	82	88	1,506	0.37	< .001
**Desire to Know Better**											
Study 1	DV: Desire to Know Better										
	Initial Romantic Liking	0.09	0.05	[-0.01, 0.20]	1.78	.077	124	130	1,439	0.14	.011
	Initial Sexual Desire	0.17	0.05	[0.06, 0.27]	3.08	.003	124	130	1,439	0.14	.011
Study 2	DV: Desire to Know Better										
	Initial Romantic Liking	0.07	0.05	[-0.03, 0.17]	1.45	.148	147	153	2,402	0.07	.158
	Initial Sexual Desire	0.08	0.05	[-0.01, 0.18]	1.80	.073	147	153	2,402	0.07	.158
Meta	DV: Know Better										
	Initial Romantic Liking	0.08	0.04	[0.01, 0.15]	2.28	.023	-	-	-	0.10	.005
	Initial Sexual Desire	0.12	0.04	[0.05, 0.19]	3.38	< .001	-	-	-	0.10	.005
**Physical Attractiveness**											
Study 1	DV: Physically Attractive Rating										
	Initial Romantic Liking	0.17	0.05	[0.06, 0.27]	3.18	.002	124	130	1,438	0.23	< .001

	Initial Sexual Desire	0.26	0.05	[0.16, 0.36]	4.94	< .001	124	130	1,438	0.23	< .001
Study 2	DV: Physically Attractive Rating										
	Initial Romantic Liking	0.05	0.04	[-0.03, 0.14]	1.19	.238	147	153	2,400	0.12	.004
	Initial Sexual Desire	0.19	0.04	[0.12, 0.27]	5.07	< .001	147	153	2,400	0.12	.004
Meta	DV: Physical Attractiveness										
	Initial Romantic Liking	0.10	0.03	[0.03, 0.16]	2.93	.003	-	-	-	0.16	< .001
	Initial Sexual Desire	0.22	0.03	[0.16, 0.28]	7.0	< .001	-	-	-	0.16	< .001
**Perceived Romantic Attraction from Match**											
Study 1	DV: Perceived Romantic Attraction										
	Initial Romantic Liking	-0.06	0.06	[-0.17, 0.06]	-0.94	.349	124	130	1,439	-0.05	.463
	Initial Sexual Desire	-0.03	0.06	[-0.16, 0.09]	-0.55	.585	124	130	1,439	-0.05	.463
Study 2	DV: Perceived Romantic Attraction										
	Initial Romantic Liking	0.01	0.05	[-0.09, 0.12]	0.21	.833	147	153	2,401	-0.01	.903
	Initial Sexual Desire	0.03	0.05	[-0.07, 0.13]	0.63	.532	147	153	2,401	-0.01	.903
Study 3	DV: Perceived Romantic Attraction										
	Initial Romantic Liking	0.25	0.07	[0.12, 0.39]	3.81	< .001	93	99	2,063	0.28	< .001
	Initial Sexual Desire	0.21	0.08	[0.06, 0.37]	2.75	.007	85	91	1,663	0.28	< .001
Meta	DV: Perceived Romantic Attraction										
	Initial Romantic Liking	0.05	0.03	[-0.01, 0.12]	1.54	.123	-	-	-	0.05	.147
	Initial Sexual Desire	0.05	0.04	[-0.02, 0.12]	1.36	.173	-	-	-	0.05	.147
**Perceived Sexual Attraction from Match**											
Study 1	DV: Perceived Sexual Attraction										
	Initial Romantic Liking	-0.02	0.07	[-0.15, 0.11]	-0.28	.778	124	130	1,439	-0.05	.463
	Initial Sexual Desire	-0.06	0.07	[-0.20, 0.08]	-0.87	.387	124	130	1,439	-0.05	.463
Study 2	DV: Perceived Sexual Attraction										
	Initial Romantic Liking	0.04	0.06	[-0.08, 0.15]	0.63	.532	147	153	2,402	-0.01	.903
	Initial Sexual Desire	0.07	0.06	[-0.04, 0.18]	1.30	.195	147	153	2,402	-0.01	.903
Study 3	DV: Perceived Sexual Attraction										
	Initial Romantic Liking	0.32	0.08	[0.16, 0.48]	4.01	< .001	89	95	1,873	0.28	< .001
	Initial Sexual Desire	0.31	0.09	[0.13, 0.48]	3.38	.001	82	88	1,501	0.28	< .001
Meta	DV: Perceived Sexual Attraction										
	Initial Romantic Liking	0.08	0.04	[0.01, 0.16]	2.17	.030	-	-	-	0.05	.147

	Initial Sexual Desire	0.07	0.04	[0.00, 0.15]	1.88	.060	-	-	-	0.05	.147

Supplementary Table S14C

Multilevel Continuous Regressions and Meta-Analyses for Primary Relationship Effects in Initial Romantic vs. Initial Sexual Desire

		Model with Only Initial Romantic Liking or Initial Sexual Desire								Original Model	
Grouping and Study	Model	β	SE	95% CI	*t*	*p*	DF	*n*	Rows	β	*p*
**Later Romantic Interest (Continuous)**											
Study 3	DV: Later Romantic Interest										
	Initial Romantic Liking	0.14	0.05	[0.04, 0.24]	2.73	.008	93	99	2,062	0.19	< .001
	Initial Sexual Desire	0.15	0.05	[0.05, 0.26]	2.97	.004	85	91	1,661	0.19	< .001
**Later Sexual Interest (Continuous)**											
Study 3	DV: Later Sexual Interest										
	Initial Romantic Liking	0.13	0.05	[0.03, 0.23]	2.70	.008	89	95	1,878	0.19	< .001
	Initial Sexual Desire	0.16	0.05	[0.07, 0.26]	3.33	.001	82	88	1,506	0.19	< .001
**Desire to Know Better**											
Study 1	DV: Desire to Know Better										
	Initial Romantic Liking	0.08	0.04	[0.00, 0.15]	1.98	.050	124	130	1,439	0.11	.007
	Initial Sexual Desire	0.09	0.04	[0.01, 0.17]	2.34	.021	124	130	1,439	0.11	.007
Study 2	DV: Desire to Know Better										
	Initial Romantic Liking	0.08	0.03	[0.02, 0.14]	2.66	.009	147	153	2,402	0.10	.001
	Initial Sexual Desire	0.06	0.03	[0.00, 0.12]	1.95	.053	147	153	2,402	0.10	.001
Meta	DV: Know Better										
	Initial Romantic Liking	0.08	0.02	[0.03, 0.13]	3.31	< .001	-	-	-	0.11	< .001
	Initial Sexual Desire	0.07	0.02	[0.02, 0.12]	2.97	.003	-	-	-	0.11	< .001
**Physical Attractiveness**											
Study 1	DV: Physically Attractive Rating										
	Initial Romantic Liking	0.05	0.04	[-0.02, 0.13]	1.45	.149	124	130	1,438	0.19	< .001
	Initial Sexual Desire	0.31	0.03	[0.24, 0.37]	9.24	< .001	124	130	1,438	0.19	< .001

Study 2	DV: Physically Attractive Rating										
	Initial Romantic Liking	0.08	0.03	[0.01, 0.15]	2.26	.025	147	153	2,400	0.18	< .001
	Initial Sexual Desire	0.24	0.03	[0.18, 0.30]	8.09	< .001	147	153	2,400	0.18	< .001
Meta	DV: Physical Attractiveness										
	Initial Romantic Liking	0.07	0.03	[0.02, 0.12]	2.65	.008	-	-	-	0.18	< .001
	Initial Sexual Desire	0.27	0.02	[0.23, 0.31]	12.18	< .001	-	-	-	0.18	< .001
**Perceived Romantic Attraction from Match**											
Study 1	DV: Perceived Romantic Attraction										
	Initial Romantic Liking	-0.02	0.04	[-0.09, 0.05]	-0.64	.525	124	130	1,439	0.02	.589
	Initial Sexual Desire	0.07	0.04	[0.00, 0.14]	1.96	.052	124	130	1,439	0.02	.589
Study 2	DV: Perceived Romantic Attraction										
	Initial Romantic Liking	-0.03	0.03	[-0.09, 0.03]	-0.99	.322	147	153	2,401	0.0	.961
	Initial Sexual Desire	0.04	0.03	[-0.03, 0.10]	1.15	.253	147	153	2,401	0.0	.961
Study 3	DV: Perceived Romantic Attraction										
	Initial Romantic Liking	0.07	0.05	[-0.03, 0.16]	1.31	.193	93	99	2,063	0.09	.042
	Initial Sexual Desire	0.10	0.05	[0.00, 0.21]	2.0	.049	85	91	1,663	0.09	.042
Meta	DV: Perceived Romantic Attraction										
	Initial Romantic Liking	-0.01	0.02	[-0.05, 0.03]	0.49	.623	-	-	-	0.03	.181
	Initial Sexual Desire	0.06	0.02	[0.02, 0.10]	2.81	.005	-	-	-	0.03	.181
**Perceived Sexual Attraction from Match**											
Study 1	DV: Perceived Sexual Attraction										
	Initial Romantic Liking	-0.01	0.03	[-0.07, 0.05]	-0.30	.764	124	130	1,439	0.02	.589
	Initial Sexual Desire	0.06	0.03	[0.00, 0.13]	1.99	.049	124	130	1,439	0.02	.589
Study 2	DV: Perceived Sexual Attraction										
	Initial Romantic Liking	-0.04	0.03	[-0.10, 0.02]	-1.27	.206	147	153	2,402	0.0	.961
	Initial Sexual Desire	0.05	0.03	[-0.01, 0.11]	1.54	.125	147	153	2,402	0.0	.961
Study 3	DV: Perceived Sexual Attraction										
	Initial Romantic Liking	0.01	0.05	[-0.09, 0.10]	0.18	.858	89	95	1,873	0.09	.042
	Initial Sexual Desire	0.07	0.05	[-0.03, 0.16]	1.36	.179	82	88	1,501	0.09	.042
Meta	DV: Perceived Sexual Attraction										
	Initial Romantic Liking	-0.02	0.02	[-0.06, 0.02]	0.96	.337	-	-	-	0.03	.181

	Initial Sexual Desire	0.06	0.02	[0.02, 0.10]	2.83	.005	-	-	-	0.03	.181

Supplementary Table S14D

Multilevel Continuous Regressions and Meta-Analyses for Secondary Partner Effects in Initial Romantic vs. Initial Sexual Desire

		Model with Only Initial Romantic Liking or Initial Sexual Desire								Original Model	
Grouping and Study	Model	β	SE	95% CI	*t*	*p*	DF	*n*	Rows	β	*p*
**Later Romantic Interest (Continuous)**											
Study 3	DV: Later Romantic Interest										
	Initial Romantic Liking	-0.07	0.08	[-0.23, 0.08]	-0.93	.353	133	139	2,062	-0.01	.900
	Initial Sexual Desire	-0.05	0.09	[-0.23, 0.12]	-0.59	.555	118	124	1,661	-0.01	.900
**Later Sexual Interest (Continuous)**											
Study 3	DV: Later Sexual Interest										
	Initial Romantic Liking	-0.03	0.09	[-0.20, 0.15]	-0.31	.757	128	134	1,878	-0.01	.900
	Initial Sexual Desire	-0.02	0.10	[-0.22, 0.17]	-0.24	.812	114	120	1,506	-0.01	.900
**Desire to Know Better**											
Study 1	DV: Desire to Know Better										
	Initial Romantic Liking	-0.09	0.06	[-0.20, 0.03]	-1.51	.135	131	137	1,439	-0.10	.092
	Initial Sexual Desire	-0.10	0.06	[-0.21, 0.02]	-1.63	.106	131	137	1,439	-0.10	.092
Study 2	DV: Desire to Know Better										
	Initial Romantic Liking	0.06	0.05	[-0.04, 0.16]	1.11	.271	158	164	2,402	0.01	.822
	Initial Sexual Desire	-0.01	0.05	[-0.11, 0.09]	-0.19	.850	158	164	2,402	0.01	.822
Meta	DV: Know Better										
	Initial Romantic Liking	-0.01	0.04	[-0.08, 0.07]	0.16	.870	-	-	-	-0.04	.340
	Initial Sexual Desire	-0.05	0.04	[-0.12, 0.03]	1.22	.223	-	-	-	-0.04	.340
**Physical Attractiveness**											
Study 1	DV: Physically Attractive Rating										
	Initial Romantic Liking	0.02	0.06	[-0.10, 0.14]	0.40	.691	131	137	1,438	-0.04	.481
	Initial Sexual Desire	-0.09	0.06	[-0.21, 0.03]	-1.47	.144	131	137	1,438	-0.04	.481
Study 2	DV: Physically Attractive Rating										

	Initial Romantic Liking	0.03	0.05	[-0.06, 0.12]	0.67	.501	158	164	2,400	0.0	.972
	Initial Sexual Desire	-0.01	0.05	[-0.10, 0.08]	-0.14	.890	158	164	2,400	0.0	.972
Meta	DV: Physical Attractiveness										
	Initial Romantic Liking	0.03	0.04	[-0.04, 0.10]	0.78	.436	-	-	-	-0.01	.691
	Initial Sexual Desire	-0.04	0.04	[-0.11, 0.04]	0.99	.320	-	-	-	-0.01	.691
**Perceived Romantic Attraction from Match**											
Study 1	DV: Perceived Romantic Attraction										
	Initial Romantic Liking	0.07	0.06	[-0.06, 0.19]	1.04	.301	131	137	1,439	0.19	.006
	Initial Sexual Desire	0.21	0.06	[0.08, 0.33]	3.32	.001	131	137	1,439	0.19	.006
Study 2	DV: Perceived Romantic Attraction										
	Initial Romantic Liking	0.20	0.05	[0.09, 0.3]	3.73	< .001	158	164	2,401	0.26	< .001
	Initial Sexual Desire	0.21	0.05	[0.10, 0.31]	3.84	< .001	158	164	2,401	0.26	< .001
Study 3	DV: Perceived Romantic Attraction										
	Initial Romantic Liking	0.01	0.07	[-0.12, 0.14]	0.16	.871	133	139	2,063	0.07	.331
	Initial Sexual Desire	0.02	0.08	[-0.13, 0.17]	0.23	.820	118	124	1,663	0.07	.331
Meta	DV: Perceived Romantic Attraction										
	Initial Romantic Liking	0.11	0.03	[0.04, 0.18]	3.11	.002	-	-	-	0.19	< .001
	Initial Sexual Desire	0.16	0.04	[0.09, 0.23]	4.57	< .001	-	-	-	0.19	< .001
**Perceived Sexual Attraction from Match**											
Study 1	DV: Perceived Sexual Attraction										
	Initial Romantic Liking	0.08	0.07	[-0.06, 0.22]	1.18	.240	131	137	1,439	0.19	.006
	Initial Sexual Desire	0.25	0.07	[0.11, 0.38]	3.56	< .001	131	137	1,439	0.19	.006
Study 2	DV: Perceived Sexual Attraction										
	Initial Romantic Liking	0.27	0.06	[0.16, 0.38]	4.69	< .001	158	164	2,402	0.26	< .001
	Initial Sexual Desire	0.29	0.06	[0.18, 0.40]	5.03	< .001	158	164	2,402	0.26	< .001
Study 3	DV: Perceived Sexual Attraction										
	Initial Romantic Liking	0.04	0.08	[-0.13, 0.20]	0.43	.667	128	134	1,873	0.07	.331
	Initial Sexual Desire	0.02	0.09	[-0.16, 0.20]	0.22	.823	114	120	1,501	0.07	.331
Meta	DV: Perceived Sexual Attraction										
	Initial Romantic Liking	0.16	0.04	[0.08, 0.24]	4.08	< .001	-	-	-	0.19	< .001
	Initial Sexual Desire	0.22	0.04	[0.15, 0.30]	5.62	< .001	-	-	-	0.19	< .001

Supplementary Table S14E

Multilevel Continuous Regressions and Meta-Analyses for Secondary Actor Effects in Initial Romantic vs. Initial Sexual Desire

		Model with Only Initial Romantic Liking or Initial Sexual Desire								Original Model	
Grouping and Study	Model	β	SE	95% CI	*t*	*p*	DF	*n*	Rows	β	*p*
**Later Romantic Interest (Continuous)**											
Study 3	DV: Later Romantic Interest										
	Initial Romantic Liking	0.05	0.05	[-0.05, 0.14]	0.99	.322	133	139	2,062	0.08	.080
	Initial Sexual Desire	0.06	0.05	[-0.04, 0.17]	1.16	.250	118	124	1,661	0.08	.080
**Later Sexual Interest (Continuous)**											
Study 3	DV: Later Sexual Interest										
	Initial Romantic Liking	0.03	0.04	[-0.06, 0.12]	0.71	.479	128	134	1,878	0.08	.080
	Initial Sexual Desire	0.04	0.05	[-0.07, 0.14]	0.70	.486	114	120	1,506	0.08	.080
**Desire to Know Better**											
Study 1	DV: Desire to Know Better										
	Initial Romantic Liking	-0.04	0.05	[-0.14, 0.06]	-0.83	.411	131	137	1,439	-0.11	.021
	Initial Sexual Desire	-0.14	0.05	[-0.23, -0.04]	-2.88	.005	131	137	1,439	-0.11	.021
Study 2	DV: Desire to Know Better										
	Initial Romantic Liking	0.01	0.04	[-0.06, 0.08]	0.29	.770	158	164	2,402	-0.05	.198
	Initial Sexual Desire	-0.07	0.04	[-0.14, 0.01]	-1.82	.071	158	164	2,402	-0.05	.198
Meta	DV: Know Better										
	Initial Romantic Liking	-0.01	0.03	[-0.07, 0.05]	0.25	.803	-	-	-	-0.07	.014
	Initial Sexual Desire	-0.09	0.03	[-0.15, -0.04]	3.20	.001	-	-	-	-0.07	.014
**Physical Attractiveness**											
Study 1	DV: Physically Attractive Rating										
	Initial Romantic Liking	-0.09	0.06	[-0.21, 0.03]	-1.45	.150	131	137	1,438	-0.19	.001
	Initial Sexual Desire	-0.22	0.06	[-0.34, -0.11]	-3.95	< .001	131	137	1,438	-0.19	.001
Study 2	DV: Physically Attractive Rating										
	Initial Romantic Liking	-0.09	0.06	[-0.20, 0.03]	-1.48	.140	158	164	2,400	-0.18	.002
	Initial Sexual Desire	-0.16	0.06	[-0.28, -0.05]	-2.82	.005	158	164	2,400	-0.18	.002
Meta	DV: Physical Attractiveness										

	Initial Romantic Liking	-0.09	0.04	[-0.17, 0.00]	2.07	.038	-	-	-	-0.19	< .001
	Initial Sexual Desire	-0.20	0.04	[-0.28, -0.12]	4.80	< .001	-	-	-	-0.19	< .001
**Perceived Romantic Attraction from Match**											
Study 1	DV: Perceived Romantic Attraction										
	Initial Romantic Liking	-0.04	0.04	[-0.11, 0.04]	-0.89	.376	131	137	1,439	-0.01	.772
	Initial Sexual Desire	0.04	0.04	[-0.04, 0.11]	0.91	.363	131	137	1,439	-0.01	.772
Study 2	DV: Perceived Romantic Attraction										
	Initial Romantic Liking	-0.03	0.03	[-0.09, 0.04]	-0.86	.391	158	164	2,401	-0.01	.838
	Initial Sexual Desire	0.01	0.03	[-0.05, 0.08]	0.43	.670	158	164	2,401	-0.01	.838
Study 3	DV: Perceived Romantic Attraction										
	Initial Romantic Liking	0.05	0.05	[-0.04, 0.14]	1.15	.251	133	139	2,063	0.08	.064
	Initial Sexual Desire	0.02	0.05	[-0.08, 0.13]	0.47	.642	118	124	1,663	0.08	.064
Meta	DV: Perceived Romantic Attraction										
	Initial Romantic Liking	-0.01	0.02	[-0.05, 0.03]	0.52	.602	-	-	-	0.01	.511
	Initial Sexual Desire	0.02	0.02	[-0.02, 0.07]	1.03	.304	-	-	-	0.01	.511
**Perceived Sexual Attraction from Match**											
Study 1	DV: Perceived Sexual Attraction										
	Initial Romantic Liking	-0.04	0.03	[-0.10, 0.03]	-1.09	.278	131	137	1,439	-0.01	.772
	Initial Sexual Desire	0.01	0.03	[-0.06, 0.07]	0.18	.858	131	137	1,439	-0.01	.772
Study 2	DV: Perceived Sexual Attraction										
	Initial Romantic Liking	-0.01	0.03	[-0.07, 0.05]	-0.41	.685	158	164	2,402	-0.01	.838
	Initial Sexual Desire	0.02	0.03	[-0.04, 0.08]	0.57	.571	158	164	2,402	-0.01	.838
Study 3	DV: Perceived Sexual Attraction										
	Initial Romantic Liking	0.06	0.04	[-0.03, 0.15]	1.40	.165	128	134	1,873	0.08	.064
	Initial Sexual Desire	0.03	0.05	[-0.07, 0.13]	0.64	.524	114	120	1,501	0.08	.064
Meta	DV: Perceived Sexual Attraction										
	Initial Romantic Liking	-0.01	0.02	[-0.04, 0.03]	0.27	.790	-	-	-	0.01	.511
	Initial Sexual Desire	0.02	0.02	[-0.03, 0.06]	0.75	.452	-	-	-	0.01	.511

Supplementary Table S14F

Multilevel Continuous Regressions and Meta-Analyses for Secondary Relationship Effects in Initial Romantic vs. Initial Sexual Desire

Grouping and Study	Model	Model with Only Initial Romantic Liking or Initial Sexual Desire								Original Model	
		β	SE	95% CI	*t*	*p*	DF	*n*	Rows	β	*p*
**Later Romantic Interest (Continuous)**											
Study 3	DV: Later Romantic Interest										
	Initial Romantic Liking	0.00	0.05	[-0.09, 0.10]	0.07	.943	133	139	2,062	0.03	.578
	Initial Sexual Desire	0.05	0.05	[-0.06, 0.16]	0.85	.397	118	124	1,661	0.03	.578
**Later Sexual Interest (Continuous)**											
Study 3	DV: Later Sexual Interest										
	Initial Romantic Liking	0.02	0.05	[-0.08, 0.11]	0.34	.732	128	134	1,878	0.03	.578
	Initial Sexual Desire	0.04	0.05	[-0.06, 0.14]	0.76	.451	114	120	1,506	0.03	.578
**Desire to Know Better**											
Study 1	DV: Desire to Know Better										
	Initial Romantic Liking	0.04	0.04	[-0.04, 0.12]	1.03	.304	131	137	1,439	0.07	.112
	Initial Sexual Desire	0.06	0.04	[-0.02, 0.14]	1.39	.168	131	137	1,439	0.07	.112
Study 2	DV: Desire to Know Better										
	Initial Romantic Liking	0.07	0.03	[0.01, 0.14]	2.27	.024	158	164	2,402	0.02	.481
	Initial Sexual Desire	-0.05	0.03	[-0.11, 0.01]	-1.57	.119	158	164	2,402	0.02	.481
Meta	DV: Know Better										
	Initial Romantic Liking	0.06	0.03	[0.01, 0.11]	2.42	.016	-	-	-	0.04	.124
	Initial Sexual Desire	-0.01	0.02	[-0.06, 0.04]	0.41	.680	-	-	-	0.04	.124
**Physical Attractiveness**											
Study 1	DV: Physically Attractive Rating										
	Initial Romantic Liking	-0.03	0.04	[-0.11, 0.05]	-0.80	.427	131	137	1,438	-0.02	.590
	Initial Sexual Desire	-0.03	0.04	[-0.11, 0.04]	-0.84	.405	131	137	1,438	-0.02	.590
Study 2	DV: Physically Attractive Rating										
	Initial Romantic Liking	0.05	0.03	[-0.02, 0.11]	1.32	.190	158	164	2,400	0.01	.839
	Initial Sexual Desire	-0.02	0.03	[-0.09, 0.05]	-0.65	.518	158	164	2,400	0.01	.839
Meta	DV: Physical Attractiveness										
	Initial Romantic Liking	0.01	0.03	[-0.04, 0.06]	0.44	.660	-	-	-	-0.01	.838
	Initial Sexual Desire	-0.03	0.03	[-0.08, 0.02]	1.04	.298	-	-	-	-0.01	.838
**Perceived Romantic Attraction from Match**											

Study 1	DV: Perceived Romantic Attraction										
	Initial Romantic Liking	0.08	0.04	[0.01, 0.15]	2.15	.033	131	137	1,439	0.12	< .001
	Initial Sexual Desire	0.15	0.03	[0.08, 0.22]	4.39	< .001	131	137	1,439	0.12	< .001
Study 2	DV: Perceived Romantic Attraction										
	Initial Romantic Liking	-0.04	0.03	[-0.1, 0.03]	-1.19	.235	158	164	2,401	0.06	.080
	Initial Sexual Desire	0.13	0.03	[0.07, 0.19]	4.11	< .001	158	164	2,401	0.06	.080
Study 3	DV: Perceived Romantic Attraction										
	Initial Romantic Liking	0.05	0.05	[-0.04, 0.14]	1.07	.288	133	139	2,063	0.06	.142
	Initial Sexual Desire	0.08	0.05	[-0.03, 0.19]	1.51	.133	118	124	1,663	0.06	.142
Meta	DV: Perceived Romantic Attraction										
	Initial Romantic Liking	0.02	0.02	[-0.02, 0.06]	1.02	.308	-	-	-	0.08	< .001
	Initial Sexual Desire	0.13	0.02	[0.09, 0.17]	6.10	< .001	-	-	-	0.08	< .001
**Perceived Sexual Attraction from Match**											
Study 1	DV: Perceived Sexual Attraction										
	Initial Romantic Liking	0.06	0.03	[0.00, 0.12]	1.86	.065	131	137	1,439	0.12	< .001
	Initial Sexual Desire	0.11	0.03	[0.05, 0.17]	3.51	< .001	131	137	1,439	0.12	< .001
Study 2	DV: Perceived Sexual Attraction										
	Initial Romantic Liking	-0.03	0.03	[-0.09, 0.03]	-0.88	.382	158	164	2,402	0.06	.080
	Initial Sexual Desire	0.12	0.03	[0.06, 0.18]	4.21	< .001	158	164	2,402	0.06	.080
Study 3	DV: Perceived Sexual Attraction										
	Initial Romantic Liking	0.04	0.04	[-0.05, 0.13]	0.85	.397	128	134	1,873	0.06	.142
	Initial Sexual Desire	0.03	0.05	[-0.07, 0.13]	0.66	.511	114	120	1,501	0.06	.142
Meta	DV: Perceived Sexual Attraction										
	Initial Romantic Liking	0.02	0.02	[-0.02, 0.06]	0.99	.323	-	-	-	0.08	< .001
	Initial Sexual Desire	0.10	0.02	[0.07, 0.14]	5.31	< .001	-	-	-	0.08	< .001

Supplementary Tables S14A-S14F. The tables show a summary of the follow up multilevel continuous regression analyses that considered the Primary and Secondary SRM effects in initial romantic liking and initial sexual desire as separate predictors of the continuous outcomes in the study, as well as the meta-analyzed effect size within each grouping of variables. These analyses also considered later romantic attraction and later sexual attraction (continuous) as separate outcome variables (Study 3 only), as well as perceived romantic and perceived sexual attraction from match (Studies 1-3) as separate outcomes. The Grouping and Study column shows the which variables were grouped together when meta-analyzed (in bold) and indicate which study the coefficients come from.

Study 1 indicates the NSDS I (college sample), Study 2 indicates NSDS II (college sample), and Study 3 indicates the Anime North Study (community sample). The IV in each analysis is indicated in the title of each table, and the dependent variable in each analysis is indicated in the Model column. The indented rows underneath each DV in the Model column indicate whether the model is for initial romantic liking or initial sexual desire; only one variable was included as a predictor at a time. β indicates the slope of the predictor (the standardized beta weight), and SE indicates the standard error of prediction. 95% CI indicates the upper and lower 95% confidence intervals (on the beta scale). *t* indicates the test statistic. *n* indicates how many unique respondents were available for each analysis. DF indicates the degrees of freedom used to calculate the *p* value for each analysis and was calculated as *n* minus one for each predictor (i.e., the one SRM independent variable) minus one for each of the four random effects (i.e., Participant ID, Partner ID, Participant ID nested within Partner ID, and Partner ID nested within Participant ID) minus one (see footnotes below for a few exceptions). Rows indicates how many rows of data were used in the analysis (note, due some participants failing to report on either initial romantic liking or initial sexual desire, the number of rows and n in each model is not always the same for initial romantic liking and initial sexual desire). The *p* values listed are not corrected for multiple comparisons because the primary purpose of each analysis was to combine them using meta-analysis, and we focus our interpretation on the meta-analyzed effect sizes and confidence intervals. For convenience, the β and *p* values for the original analysis (i.e., the models that used the average of initial romantic and initial sexual desire, as well as the average of romantic and sexual desire for the relevant outcome variables) are also reported in the table (for more information about the original analyses, see Supplementary Tables S3A-S3F and Tables 2-3 in the main manuscript).

Supplementary Table S15

Counts of Dichotomous Outcome Variables

Measure	Study 1 NSDS I		Study 2 NSDS II		Study 3 Anime North	
	Yes	No	Yes	No	Yes	No
First Contact Initiation	133	279*	120	384*	60	222*
Hanging Out / Corresponding†	579	863	243<br>556	2,154<br>1,847	363	1,945
Later Romantic Interest	348	1,094	516	1,887	-	-

Supplementary Table S15. The table shows the numbers of Yes and No answers for the dichotomous outcome variables collected in the three studies. See the primary text for a full description of how these variables were determined. Note: Later Romantic Interest was a continuous variable in Study 3 and the cells marked with a dash (-) indicate the cells are intentionally left blank.

* In Study 1-2, participants were classified as No if their match initiated the first contact (Study 1: n = 133; Study 2: n = 120) or if neither they nor their matched contacted each other (Study 1: n = 146; Study 2: n = 264). In Study 3, participants were classified as No if they reported that their match initiated contact that week (n = 64) or if they reported that they did not have contact with their match that week (n = 158).

† In Study 2, there were separate items pertaining to Hanging Out (first row) and Corresponding (second row). In Study 1 and Study 3, only one item was asked pertaining to Hanging Out or Corresponding.

Reference Cited in Supplementary Information

1. M. S. Clark, L. A. Beck, O. R. Aragón, "Relationship initiation: Bridging the gap between initial attraction and well-functioning communal relationships" in APA handbook of contemporary family psychology: Foundations, methods, and contemporary issues across the lifespan, Vol. 1. (American Psychological Association, Washington, DC, US, 2019), 10.1037/0000099-023, pp. 409-425.
2. C. W. Backman, P. F. Secord, The effect of perceived liking on interpersonal attraction. *Human Relations* **12**, 379-384 (1959).
3. A. J. Lee, M. J. Sidari, S. C. Murphy, J. M. Sherlock, B. P. Zietsch, Sex Differences in Misperceptions of Sexual Interest Can Be Explained by Sociosexual Orientation and Men Projecting Their Own Interest Onto Women. *Psychological Science* **31**, 184-192 (2020).
4. E. P. Lemay, M. S. Clark, How the head liberates the heart: projection of communal responsiveness guides relationship promotion. *Journal of Personality and Social Psychology* **94**, 647 (2008).
5. E. P. Lemay, M. S. Clark, A. Greenberg, What is beautiful is good because what is beautiful is desired: Physical attractiveness stereotyping as projection of interpersonal goals. *Personality and Social Psychology Bulletin* **36**, 339-353 (2010).
6. E. P. Lemay, N. R. Wolf, Projection of Romantic and Sexual Desire in Opposite-Sex Friendships: How Wishful Thinking Creates a Self-Fulfilling Prophecy. *Personality and Social Psychology Bulletin* **42**, 864-878 (2016).
7. S. Sprecher, The role of expectations for liking and other positive Affiliative outcomes in the get-acquainted process that occurs over Computer-mediated video communication. *Current Psychology* 10.1007/s12144-021-01466-4 (2021).
8. T. M. Newcomb, The prediction of interpersonal attraction. *American Psychologist* **11**, 575-586 (1956).
9. M. Rossignac-Milon, N. Bolger, K. S. Zee, E. J. Boothby, E. T. Higgins, Merged minds: Generalized shared reality in dyadic relationships. *Journal of Personality and Social Psychology* **120**, 882 (2021).
10. L. E. Park, A. F. Young, P. W. Eastwick, J. D. Troisi, L. Streamer, Desirable but not smart: Preference for smarter romantic partners impairs women's STEM outcomes. *Journal of Applied Social Psychology* **46**, 158-179 (2016).
11. M. S. Ben-Shachar, D. Lüdecke, D. Makowski, effectsize: Estimation of effect size indices and standardized parameters. *Journal of Open Source Software* **5**, 2815 (2020).
12. A. Signorell *et al.* (2022) DescTools: Tools for Descriptive Statistics.
13. D. Bates, M. Mächler, B. Bolker, S. Walker, Fitting linear mixed-effects models using lme4. *arXiv preprint arXiv:1406.5823* (2014).
14. P. W. Eastwick, E. J. Finkel, D. Mochon, D. Ariely, Selective versus unselective romantic desire: Not all reciprocity is created equal. *Psychological Science* **18**, 317-319 (2007).
15. D. A. Kenny, "Appendix A. Details of the Social Relations Model" in Interpersonal Perception: The Foundation of Social Relationships. (The Guilford Press, 2019).
